# Supplementary figures and images for: Sex chromosome turnover and structural genome divergence shape meiotic outcomes in hybridizing Cobitis
Source: Gigascience. 2026 Mar 24;15:giag031. doi: 10.1093/gigascience/giag031 (PMC13175044; doi:10.1093/gigascience/giag031)

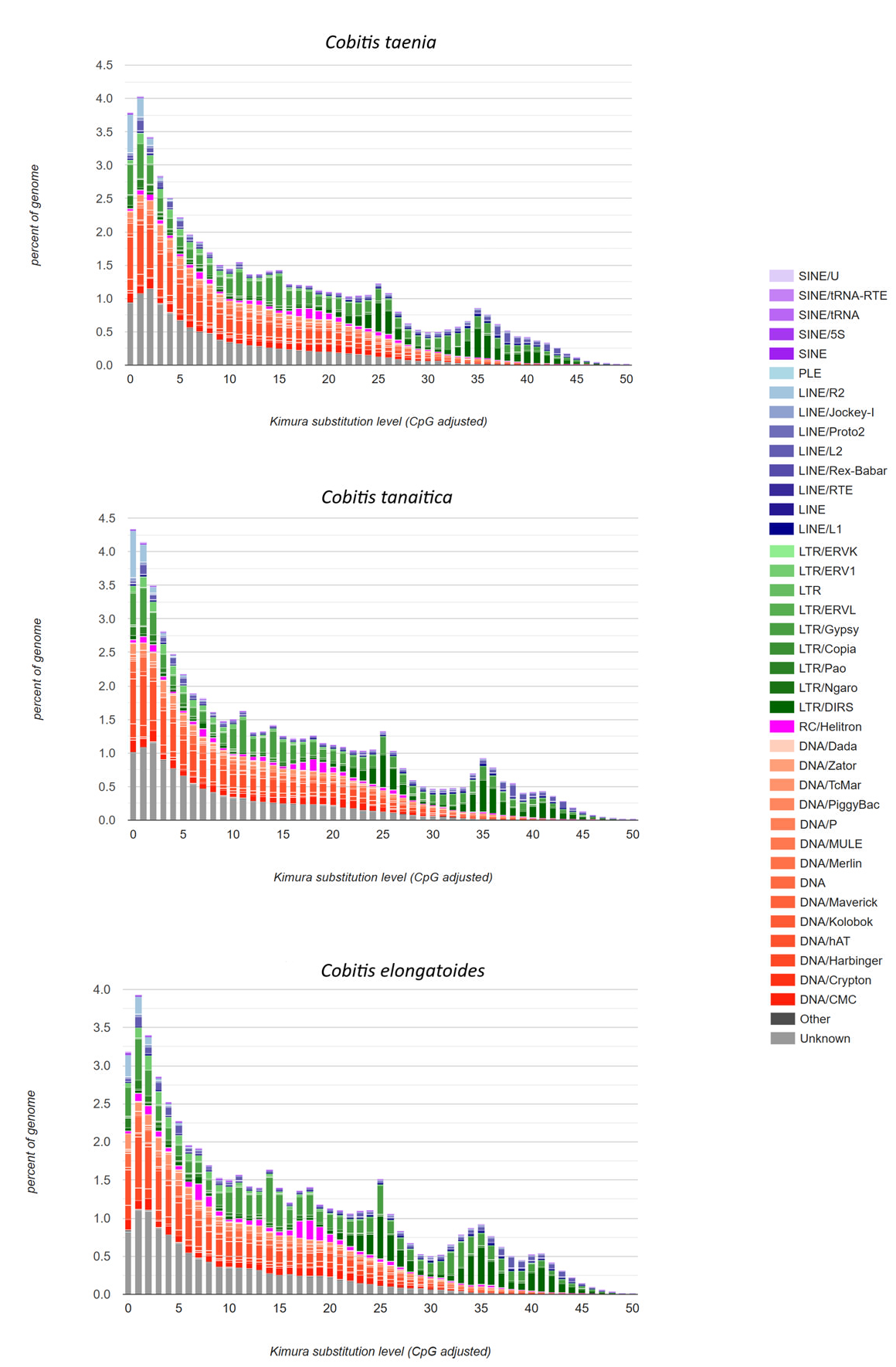

Supplement: giag031_Supplemental_Files [file giag031_supplemental_files.zip › Supplementary_Figure_S1.tiff]

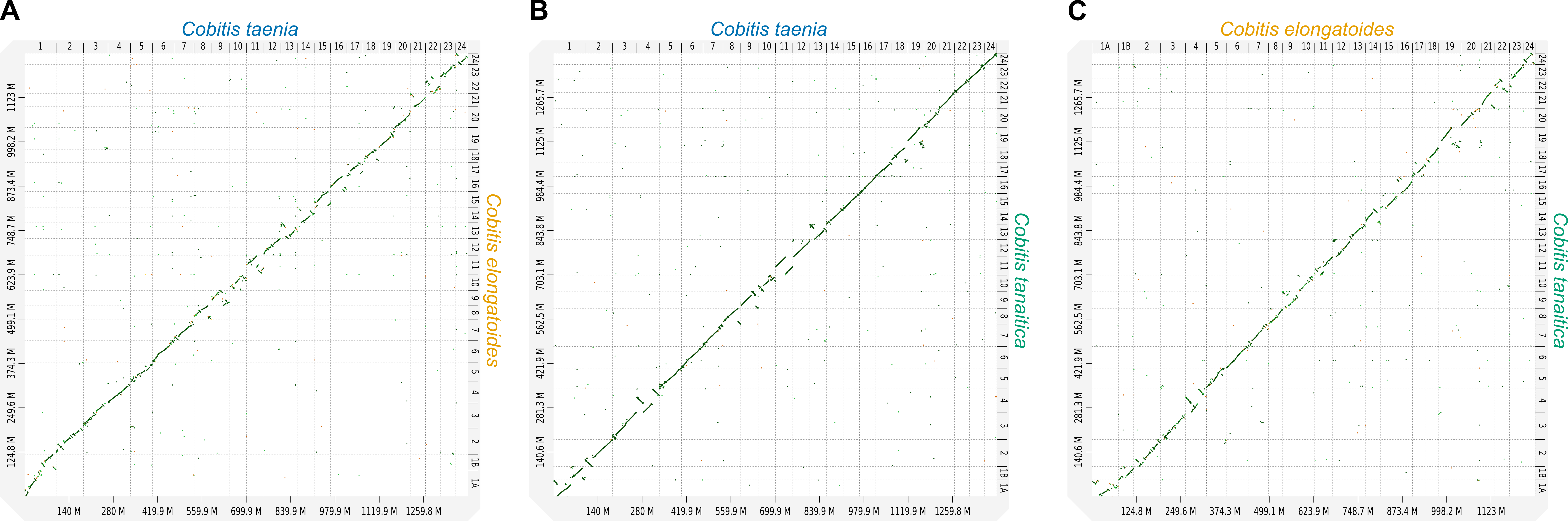

Supplement: giag031_Supplemental_Files [file giag031_supplemental_files.zip › Supplementary_Figure_S2.tiff]
