# Supplementary material for: Sex chromosome turnover and structural genome divergence shape meiotic outcomes in hybridizing Cobitis
Source: Gigascience. 2026 Mar 24;15:giag031. doi: 10.1093/gigascience/giag031 (PMC13175044; doi:10.1093/gigascience/giag031)
Supplement: giag031_GIGA-D-25-00241_original_submission [file giag031_giga-d-25-00241_original_submission.pdf]

GigaScience

Sex Chromosome Turnover and Structural Genome Divergence Shapes Meiotic Outcomes in Hybridising Cobitis

--Manuscript Draft--

**Manuscript Number:**	GIGA-D-25-00241
**Full Title:**	Sex Chromosome Turnover and Structural Genome Divergence Shapes Meiotic Outcomes in Hybridising Cobitis
**Article Type:**	Research

Funding Information:

Charles University Research Centre program (UNCE/24/SCI/006)	Dr. Stephen A. Schlebusch
Grantová Agentura České Republiky (24-12217S)	Dr. Karel Janko
HORIZON EUROPE Marie Sklodowska-Curie Actions (101081195)	Dr. Vladimir Trifonov
Spanish Ministry of Science and Innovation (PID2020-112557GB-I00)	Prof. Aurora Ruiz-Herrera
Agència de Gestió d'Ajuts Universitaris i de Recerca (2021SGR00122)	Prof. Aurora Ruiz-Herrera
Ministerio de Economía y Competitividad (PRE-2018-083257)	Dr. Lucia Álvarez-González
Ministerio de Economía y Competitividad (PRE-C-2021-0083)	MSc Gala Pujol
Grantová Agentura, Univerzita Karlova (314222)	MSc Zuzana Halenková

Abstract:

Background: Various metazoan lineages have shown that hybridization between divergent species can result in meiotic aberrations and the production of unreduced gametes, ultimately enabling the emergence of asexual reproduction. Yet, it remains poorly understood how such asexual hybrids cope with co-inherited differences in sex determination systems, diverged regulatory gene networks, and chromosomal incompatibilities—especially in the context of increased ploidy. Addressing these questions requires high-quality, chromosome-level reference genomes of the parental species involved in hybrid formation.
Findings: To this end, we present the first chromosome-level genome assemblies for three hybridising Cobitis species (C. elongatoides, C. taenia, and C. tanaitica), providing a comprehensive framework for investigating the genomic and cytogenetic basis of hybrid sterility and the transition to asexuality. By integrating genome scaffolding, male/female pooled sequencing, and molecular cytogenetics, we uncover extensive structural variation among homologous chromosomes of the three species, despite overall karyotype conservation. Population-level Pool-Seq analyses further revealed that each species possesses distinct, non-homologous sex chromosomes, highlighting rapid sex chromosome turnover even among recently diverged lineages. The newly generated assemblies enabled the design of chromosome-specific painting probes, which we applied to meiotic metaphase I spreads of diploid hybrids. This approach revealed striking differences in the pairing success of orthologous chromosomes, with some frequently forming bivalents, while others failed to pair and remained as univalents.
Conclusions: Our results demonstrate that chromosome-specific features, shaped by structural evolution and sex-linked divergence, contribute unequally to hybrid meiotic failure. Together, this work provides a high-resolution genomic and cytogenetic framework to understand how interspecific hybridisation gives rise to clonality, and how the architecture of inherited parental genomes shapes the success or breakdown of meiosis in hybrid vertebrates.

Corresponding Author:

Lucija Andjel
Institute of Animal Physiology and Genetics CAS: Ustav zivocisne fyziologie a genetiky Akademie ved Ceske republiky

Powered by Editorial Manager® and ProduXion Manager® from Aries Systems Corporation

	Libechov, CZECH REPUBLIC
**Corresponding Author Secondary Information:**	
**Corresponding Author's Institution:**	Institute of Animal Physiology and Genetics CAS: Ustav zivocisne fyziologie a genetiky Akademie ved Ceske republiky
**Corresponding Author's Secondary Institution:**	
**First Author:**	Stephen A. Schlebusch, PhD
**First Author Secondary Information:**	
**Order of Authors:**	Stephen A. Schlebusch, PhD
	Vladimir Trifonov, PhD
	Zuzana Halenková
	Marharyta Klianitskaya
	Dmitrij Dedukh, PhD
	Aurora Ruiz-Herrera, PhD
	Lucia Álvarez-González, PhD
	Gala Pujol
	Eva Hřibová, PhD
	Lucija Andjel
	Oldřich Bartoš
	Petr Pajer, PhD
	Tomáš Tichopád, PhD
	Daniel Kulik
	Jan Kotusz
	Marie Kaštánková Doležálková, PhD
	Astrid Böhne
	Anatolie Marta
	Patrik Horna
	Radka Reifova
	Yann Guiguen
	Heiner Kuhl
	Jan Pačes
	Karel Janko
**Order of Authors Secondary Information:**	

**Additional Information:**	
**Question**	**Response**
Are you submitting this manuscript to a special series or article collection?	No
**Experimental design and statistics**	Yes

Full details of the experimental design and statistical methods used should be given in the Methods section, as detailed in our Minimum Standards Reporting Checklist. Information essential to interpreting the data presented should be made available in the figure legends.<br><br>Have you included all the information requested in your manuscript?	
**Resources**<br><br>A description of all resources used, including antibodies, cell lines, animals and software tools, with enough information to allow them to be uniquely identified, should be included in the Methods section. Authors are strongly encouraged to cite Research Resource Identifiers (RRIDs) for antibodies, model organisms and tools, where possible.<br><br>Have you included the information requested as detailed in our Minimum Standards Reporting Checklist?	Yes
**Availability of data and materials**<br><br>All datasets and code on which the conclusions of the paper rely must be either included in your submission or deposited in publicly available repositories (where available and ethically appropriate), referencing such data using a unique identifier in the references and in the "Availability of Data and Materials" section of your manuscript.<br><br>Have you have met the above requirement as detailed in our Minimum Standards Reporting Checklist?	Yes
GigaScience has policies and guidelines in place for the use of generative AI-writing tools such as ChatGPT. If you have used such writing tools to assist with	No

writing the manuscript this must be declared and cited in the text. Authors should not list AI-writing tools and other AI-assisted technologies as an author or co-author and should acknowledge that they are fully responsible for text generated or refined by AI-writing tools.<p><br><br>A summary of use (particularly in the introduction or among methods) needs to be included at the end of the paper, and the outputs should also be included as a supplementary file hosted in GigaDB or other open repositories. Please read our guidelines for more information. <p><br><br>By submitting to GigaScience, you are aware of the journal's AI-writing tools policy, and if you have declared use of such tools below, you have acknowledged this where appropriate in your manuscript and have made a summary of use and outputs available. <p><br><b>AI-assisted writing tools have been used in the preparation of this manuscript?	

Manuscript

Click here to access/download;Manuscript;Schlebusch et al. 2025.docx

Sex Chromosome Turnover and Structural Genome Divergence Shapes Meiotic Outcomes in Hybridising *Cobitis*

Stephen A. Schlebusch[1,2], Vladimir Trifonov[2], Zuzana Halenková[1], Marharyta Klianitskaya[3], Dmitrij Dedukh[2], Aurora Ruiz-Herrera[4,5], Lucia Álvarez-González[4,5], Gala Pujol[4,5], Eva Hřibová[6], Lucija Andjel[2,7], Oldřich Bartoš[2,8], Petr Pajer[3,8], Tomáš Tichopád[2,9], Daniel Kulik[2,10], Jan Kotusz[10], Marie Kaštánková Doležálková[2], Astrid Böhne[11], Anatolie Marta[2], Patrik Horna[2], Radka Reifová[1], Yann Guiguen[12], Heiner Kuhl[13], Jan Pačes[3,*], Karel Janko[2,14,*]

Affiliations:

[1] Department of Zoology, Faculty of Science, Charles University, 12800 Prague, Czech Republic

[2] Laboratory of Non-Mendelian Evolution, Institute of Animal Physiology and Genetics, The Czech Academy of Sciences, 27721 Liběchov, Czech Republic

[3] Institute of Molecular Genetics of the Czech Academy of Sciences, 14220 Prague, Czech Republic

[4] Genome Integrity and Instability Group, Institut de Biotecnologia i Biomedicina (IBB), Universitat Autònoma de Barcelona (UAB), Cerdanyola del Vallès, 08193, Spain

[5] Departament de Biologia Cellular, Fisiologia i Immunologia, Universitat Autònoma de Barcelona (UAB), Cerdanyola del Vallès, 08193, Spain

[6] Institute of Experimental Botany of the Czech Academy of Sciences, Centre of the Region Haná for Biotechnological and Agricultural Research, 77900 Olomouc, Czech Republic

[7] Department of Ecology, Faculty of Science, Charles University, 12800 Prague, Czech Republic

[8] Military Health Institute, 16200 Prague, Czech Republic

[9] University of South Bohemia in České Budějovice, Faculty of Fisheries and Protection of Waters, South Bohemian Research Centre of Aquaculture and Biodiversity of Hydrocenoses, 38925 Vodňany, Czech Republic

[10] Museum of Natural History, University of Wrocław, 50335 Wrocław, Poland

[11] Centre for Molecular Biodiversity Research, Leibniz Institute for the Analysis of Biodiversity Change, Museum Koenig Bonn, 53113 Bonn, Germany

[12] INRAE, LPGP, 35000 Rennes, France

[13] Leibniz-Institute of Freshwater Ecology and Inland Fisheries, 12587 Berlin, Germany

[14] Department of Biology and Ecology, Faculty of Science, University of Ostrava, 70103 Ostrava, Czech Republic

*Correspondence address. Jan Pačes, Institute of Molecular Genetics of the Czech Academy of Sciences, 14220 Prague, Czech Republic, E-mail: hpaces@img.cas.cz; Karel Janko, Laboratory of Non-Mendelian Evolution, Institute of Animal Physiology and Genetics, The Czech Academy of Sciences, 27721 Liběchov, Czech Republic, E-mail: janko@iapg.cas.cz.

Abstract

Background: Various metazoan lineages have shown that hybridisation between divergent species can result in meiotic aberrations and the emergence of asexual reproduction. Yet, it remains poorly understood how such asexual hybrids cope with co-inherited differences in sex

determination systems, diverged regulatory gene networks, and chromosomal incompatibilities—especially in the context of increased ploidy. Addressing these questions requires high-quality, chromosome-level reference genomes of the parental species involved in hybrid formation.

Findings: To this end, we present the first chromosome-level genome assemblies for three hybridising *Cobitis* species (*C. elongatoides*, *C. taenia*, and *C. tanaitica*), providing a comprehensive framework for investigating the genomic and cytogenetic basis of hybrid sterility and the transition to asexuality. By integrating genome scaffolding, male/female pooled sequencing (Pool-Seq), and molecular cytogenetics, we uncover extensive structural variation among homologous chromosomes of the three species, despite overall karyotype conservation. Population-level Pool-Seq analyses further revealed that each species possesses distinct, non-homologous sex chromosomes, highlighting rapid sex chromosome turnover even among recently diverged lineages. The newly generated assemblies enabled the design of chromosome-specific painting probes, which we applied to meiotic metaphase I spreads of diploid hybrids. This approach revealed striking differences in the pairing success of orthologous chromosomes.

Conclusions: Our results demonstrate that chromosome-specific features contribute unequally to hybrid meiotic failure. Together, this work provides a high-resolution genomic and cytogenetic framework to understand how interspecific hybridisation gives rise to clonality, and how the architecture of inherited parental genomes shapes the success or breakdown of meiosis in hybrid vertebrates.

Keywords: speciation, asexual reproduction, polyploidy, hybrid sterility, loaches, sex determination, chromosome evolution

Background

Reproduction, the ability to transmit a genome from one generation to the next, is fundamental to all living organisms. In metazoans, this primarily occurs through the fusion of reduced gametes produced by meiotic divisions involving recombination. This process likely evolved as an efficient mechanism for DNA repair, but it also confers significant advantages over non-recombinant modes, generating variability to evade fast-evolving pathogens, eliminating deleterious alleles, and facilitating genetic exchange within a species' gene pool [1]. However, reproductive modes vary widely among taxa, and even meiosis and recombination frequencies are optimised for specific genomic regions, environments, or sexes [1–4]. Hybridisation between diverged (sub)species may also bring together karyotypes with accumulated structural variants (SVs), which may not only affect hybrid fertility through meiotic impairment [5,6] but also lead to the introgression of linked variants with limited recombination, potentially resulting in the formation of supergenes [4]. In extreme cases, even complex organisms such as animals—and vertebrates in particular—may abandon sexual reproduction altogether and instead produce unreduced, usually non-recombinant, gametes (e.g. [7]).

These so-called 'asexual' lineages do not represent a simply definable group. They are scattered across the tree of life and employ a wide spectrum of independently arisen cytological mechanisms for gamete production, ranging from completely ameiotic processes (apomixis) to those involving altered versions of meiotic divisions (automixis) [8,9], with different genetic consequences for the evolution and ecology of these lineages. Yet, despite the great variability of asexual organisms and their polyphyletic origins, asexual lineages have proven to be excellent natural models for studying fundamental biological questions—such as the evolutionary consequences of sex and recombination, mutation accumulation, coevolution with pathogens, and more (e.g. [10]).

Interestingly, recent research has identified several patterns in the mechanisms behind asexual reproduction, with unrelated asexual organisms often sharing similar cytogenetic and molecular traits. For example, the genomes of asexual organisms often do not accumulate deleterious mutations as quickly as originally expected [11–14]. The abandonment of sex frequently coincides with interspecific hybridisation and, for reasons still unknown, is

correlated with increasing divergence between the hybridising sexual species [15,16]. Many "asexual" hybrids produce unreduced gametes through an apparently shared cytological mechanism—premeiotic endoreplication (PMER)—in which the maternal genetic material is duplicated prior to meiosis. As a result, recombination occurs between identical sister chromosomes, supposedly leading to no genetic variability among the progeny, apart from de novo mutations [17–19]. This process appears to be sex-specific and is typically confined to females, whereas hybrid males from the same crosses are usually unable to produce clonal gametes [20,21].

Recent advances in genomics have motivated many researchers to investigate how the lack of effective genome mixing following the switch to asexuality impacts genome structure and gene expression. Interesting patterns, consistent across some independently arisen lineages, have been found (e.g. [22]). For instance, genomes originally inherited from sexual ancestors may not remain static but instead, due to effects such as gene conversion, tend to accumulate structural changes, leading to—among other effects—a loss of heterozygosity [11,23,24]. Comparisons with sexual ancestor species have shown that gene conversions in asexual genomes are closely correlated with the expression of orthologous alleles, base composition of loci, and specific gene functions [23]. This opens the question of whether such changes may be detrimental or adaptive, potentially contributing to, for example, the balancing and optimization of components within regulatory networks in hybrid genomes [25].

Understanding genome evolution under restricted recombination—especially in asexual organisms—remains a dynamic and challenging field. Robust comparative analyses between asexual lineages and their direct sexual ancestors are essential, yet many lineages still lack well-assembled and annotated genomes, let alone detailed characterisation of structural variants and the distribution of repetitive elements. Furthermore, many asexual lineages do not have identifiable ancestral sexual counterparts or have arisen through interspecific hybridisation, combining genomes from diverged sexual species. These factors complicate comparative genomic studies. Without the ability to directly compare the genotypes

and phenotypes of contemporary asexual strains to their sexual progenitors, it becomes difficult to determine whether observed patterns across unrelated asexual lineages are coincidental or reflect deeply conserved mechanisms that enable parallel transitions to asexuality across distant taxa [20,26–28]. Acquiring high-quality reference genomes of carefully determined and selected parental species and comparing them to their derived asexuals is essential for addressing these questions.

The spined loaches of the genus *Cobitis* serve as an appealing model for understanding the link between speciation, hybridisation, sex, and asexuality, with several instances of interspecific hybridisation found across Eurasian hybrid zones, between species that diverged between 1 and 15 million years ago (Figure 1). The outcome of hybridisation differs strikingly depending on the relatedness between parental species. Closely related species form fertile and sexually reproducing hybrids [15,27]. In contrast, hybridisation between distant loach species often results in sexually asymmetric fertility. Hybrid males are typically sterile due to chromosomal mispairing that disrupts meiosis, preventing the formation of spermatids—although early-stage germ cells such as spermatogonia and spermatocytes remain present. Hybrid females, however, retain fertility but reproduce asexually [15,18]. These asexual females exist in diploid, triploid, and tetraploid forms and produce clonal eggs via PMER. The basis for this stark sexual asymmetry in fertility and mode of reproduction remains unclear. Intriguingly, transplantation experiments have shown that spermatogonial germ cells from sterile hybrid males, when introduced into female gonads, are capable of undergoing PMER. This suggests that the female gonadal environment may play a critical role in enabling clonal reproduction, even in male-derived germ cells [21].

In any case, previous studies showed that hybridisation leading to asexuality has been ongoing throughout the Pleistocene, resulting in many clonal strains, some of which originated recently while others are several hundred thousand generations old [29]. While clonal hybrids tend to conserve their inherited parental karyotype structure for thousands of generations without significant restructuring [30], they are subject to a gradual loss of heterozygosity, which

accumulates selectively in certain loci depending on the relative transcription of the alleles [23].

These patterns raise important questions about the extent to which genome evolution in asexuals is driven by the overall system of heredity without recombination versus being determined by specific properties and levels of divergence between the genomes inherited from sexual ancestors. These genomic differences include specific genetic sex determination mechanisms, SVs affecting chromosome pairing, divergence in repetitive elements, gene regulatory networks, and epigenetic signalling [21,31].

The aim of this study is to generate high-quality, chromosome-level genome assemblies for three parental *Cobitis* species—*C. elongatoides*, *C. taenia*, and *C. tanaitica*—which serve as the sexual progenitors of various asexual hybrid lineages [15,27]. These assemblies provide a necessary foundation for investigating the genomic basis of hybrid sterility and asexuality, focusing on four key aspects: (i) the extent and nature of SVs accumulated between species and their impact on chromosomal compatibility; (ii) divergence in repeat element content and dynamics across lineages; (iii) identification and comparative analysis of genetic sex determination systems; and (iv) chromosomal pairing behaviour in hybrid males as a mechanistic insight into meiotic failure.

Methods

Sample collection

For this work, specimens from three *Cobitis* species (*C. elongatoides*, *C. taenia*, and *C. tanaitica*) were required. All of these individuals, as well as their investigated hybrids, belonged to laboratory strains originated from natural populations, that were contained at breeding facilities at the Institute of Animal Physiology and Genetics of the Czech Academy of Sciences under the permission 16OZ2636/202-18134 MZe-24154/2021-18134 (see Supplementary Table S1 for detailed individual information and Figure 1 for collection distribution). The specimens were categorised into taxonomic units using published

microsatellite markers and their ploidy determined by flow cytometry and karyotype verified by standard cytogenetic means as previously described [29].

RNA and DNA isolation and sequencing

A phenol/chloroform extraction protocol [32] was used to extract DNA from ~1 g of skeletal muscle for Oxford Nanopore (ONT) sequencing from one individual per species and Illumina sequencing from a second individual. DNA quality and quantity was assessed using a Qubit double-stranded DNA HS Assay Kit (Invitrogen, Thermo Fisher Scientific), agarose gel electrophoresis and an Agilent Bioanalyzer 2100 (Agilent Technologies). For *C. taenia*, DNA was used for Oxford Nanopore Technology (ONT) sequencing library preparation using the 1D Genomic DNA by ligation kit (SQK-LSK108) and the library was run on MinION device using the FLO-MIN107 R9 Flow Cells according to manufacturer's instructions. For *C. elongatoides* and *C. tanaitica*, which were analysed later, libraries for ONT sequencing were prepared using a ligation sequencing kit (SQK-LSK109, Oxford Nanopore) and sequenced on a Nanopore GridION instrument (FLO-MIN106 flow cell) according to manufacturer's instructions. Illumina sequencing was obtained with Illumina NextSeq 2000 platform according to manufacturer’s instructions using NextSeq 1000/2000 P1 XLEAP-SBS Reagent Kit. This generated paired-end reads with a length of 2 × 250 bp. Raw sequencing data were obtained in FASTQ format and subjected to quality control and downstream bioinformatic analyses.

For HiC, a spleen from the same specimen used for ONT was dissected and sent on dry ice to the Dovetail (*C. taenia*) and IAB (*C. elongatoides, C. tanaitica*) companies for OMNI-C library construction.

To investigate putative sex determination in studied species, we sequenced whole genomes of additional individuals. First, we isolated gDNA from a total of 122 male and female specimens of *C. taenia* and *C. elongatoides* for sex specific marker analysis with DNAeasy Blood&Tissue kit (Qiagen). The isolates were pooled equimolarly into four pools per species reflecting their sex and geographical origin (see Supplementary Table S1) and sequenced with Illumina 150bp paired end sequencing by IAB company. Due to the limited number of *C.*

tanaitica in our collections, we isolated gDNA from additional 3 females and 2 males, and these individuals were sequenced individually.

To obtain mRNA data for gene annotation, we extracted mRNA from brain and gonadal tissues from several individuals of *C. elongatoides, C. taenia* and *C. tanaitica* using the TRIzol protocol [33]. Libraries were then prepared using the Lexogen SENSE Total RNA-Seq Library Prep Kit and sequenced on a NextSeq 550 with a read length of 75 bp in paired-end mode. This mRNA was used in combination with published data [25] for comprehensive gene annotation.

Genome assembly

Oxford Nanopore sequencing that forms the basis of the three genome assemblies was based on a single male individual from each species. A *C. taenia* assembly was initially performed using the short reads mentioned above and *de Brujn* assemblers. To minimise misassemblies, we assembled the short reads with ABySS v2.0 [34] and SOAPdenovo v2.04 [35] and split the contigs at positions where the two assemblies disagreed. The contigs from the initial consensus assembly were combined with the nanopore reads and assembled using Flye4 v2.9.1 [36], followed by Nanopolish v0.13.1 [37]. The assembly was then improved by two runs of Pilon v1.24 [38] using the Illumina sequencing data. Because we had reads from several individuals, we normalised the number of reads per individual and mapped them on the polished assembly using bwa v0.7.17 [39] before variant calling with Bcftools v1.10.2 [40]. Each SNP was then modified to the major allele if necessary (custom script). Note that for obtaining the genome assemblies of *C. tanaitica* and *C. elongatoides*, only Flye4 with nanopore reads was used to create the primary assemblies before implementing aforementioned downstream analyses.

Chromosome-level De novo assembly of Cobitis genomes

Primary assemblies (N50 ~150 Kbp) were then scaffolded to a chromosomal level using Hi-C data while following the Juicer-3D-DNA pipeline v201008 [41]. The Hi-C reads for

the three species were mapped against their respective fragmented draft genome using Burrows-Wheeler Aligner (bwa) [39] and reads with a MAPQ < 30 were discarded. Based on the contact frequencies, 3D-DNA was run with default parameters to construct the final superscaffolds [41]. Final assembly stats were calculated with the script stats.sh included in the sequence-analysis package BBmap [42]. Hi-C matrices were built at 500 Kbp resolution by remapping Hi-C reads against the final assembly using Juicer with default parameters. Small scaffolds were discarded and only chromosome-level superscaffolds (> 30 Mbp), organised by size, were included. Lastly, matrices were normalised, corrected, and plotted using 'hicNomalize', 'hicCorrect' and 'hicPlotMatrix' from HiCExplorer (v.3.7) [43]. First eigenvector values were calculated using the tool 'fanc compartments' from the HiC analysis tools package, FAN-C (v0.9.1) [44]. Moreover, topologically associated domains (TADs) were detected with the tool 'hicFindTADs' from HiCExplorer (v.3.7) [43]. For both analyses, normalised 50 Kbp matrices were employed as input, as previously described [45].

Gene annotation

Ab initio gene prediction was performed by Dovetail as follows: repeat families found in the genome assembly of *Cobitis taenia* were identified *de novo* and classified using the software package RepeatModeler v2.0.1 [46]. RepeatModeler depends on the programs Recon v1.08 [47] and RepeatScout v1.0.6 [48] for the *de novo* identification of repeats within the genome. The custom repeat library obtained from RepeatModeler was used to discover, identify and mask the repeats in the assembly file using RepeatMasker v4.1.0 [49]. Coding sequences from *Triplophysa tibetana*, *Astyanax mexicanus,* and *Danio rerio* were used to train the initial *ab initio* model for *C. taenia* using Augustus v2.5.5 [50]. Six rounds of prediction optimisation were done with Augustus. The same coding sequences were also used to train a separate *ab initio* model for *Cobitis taenia* using Snap v2006-07-28 [51]. RNAseq reads (Supplementary Table S1) were mapped onto the genome using STAR v2.7 [52] and intron hints generated with the bam2hints tools within Augustus [53–55]. Maker [56], Snap and Augustus (with intron-exon boundary hints provided from RNA-Seq) were then used to predict

genes in the repeat-masked reference genome. To help guide the prediction process, Swiss-Prot peptide sequences from the UniProt database [57] were downloaded and used in conjunction with the protein sequences from *T. tibetana*, *A. mexicanus,* and *D. rerio* to generate peptide evidence in the Maker pipeline. Only genes that were predicted by both Snap and Augustus were retained in the final gene annotation. To help assess the quality of the gene prediction, AED scores were generated for each of the predicted genes as part of the Maker pipeline. Genes were further characterised for their putative function by performing a Blast [58] search of the peptide sequences against the UniProt database. tRNAs were predicted using tRNAscan-SE v2.05 [59].

Initial gene prediction metrics were 35,082 genes. A completeness analysis showed that only 74.9% of BUSCO genes were complete, 5.9% were partially present and 19.2% were missing. This was lower than the 95% of BUSCO genes found by BUSCO if the analysis was done directly on the genome.

The annotation was improved by extending the transcripts and generating new ones using StringTie v2.2.1 [60], followed by Transdecoder v5.7.1 [61]. We used published [25,27] as well as newly obtained mRNA data from muscle, liver, gonad, and spleen tissue from *C. taenia* and *C. elongatoides* for annotation (Supplementary Table S1). Mapping was done by STAR v2.7.10b. Both Dovetail annotation and StringTie annotation were merged using AGAT [62]. The BUSCO genes were improved to 93.3% completeness (C:), with 2.3% fragmented (F:) and 4.4% missing (M:) genes.

Because our repetitive element annotation (next section) differs from the masking used for *ab initio* gene prediction by Dovetail, we deleted genes that were identified as being repetitive elements. Annotation was improved by using HANNO v0.5 (Kuhl, 2024) to integrate evidence from *C. tanaitica* and *C. elongatoides* RNAseq data as well as related species' RefSeq mRNAs and proteins (*Misgurnus anguillicaudatus* and *Paramisgurnus dabryanus*) for gene modelling and functional annotation. All the species specific RNAseq data were mapped to the specific *Cobitis* genomes using Hisat2 [64] and transcript models were built using StringTie v2.2.1 [60]. In a first run, the resulting transcript GTF was added to the HANNO v0.5

pipeline (parameter -g) alongside RefSeq protein (-p) and mRNA evidence (-r) from *M. anguillicaudatus* (GCF_027580225.1) and *P. dabryanus* (GCF_030506205.1). A second run of HANNO was performed without RefSeq mRNAs, which successfully identified a few missing genes which were added to the results from the first run. This successfully reduced missing BUSCO genes to 6.2% for *C. elongatoides* and to 5.0% for *C. tanaitica*.

Repetitive element annotation

In order to identify and annotate the repetitive elements, initial consensus sequences were generated using the Dfam TETools container v1.87 [65] (running on Docker 24.0.5), which packages RepeatModeler v2.0.5 and RepeatMasker v4.1.5 together with Dfam 3.7 (curated portion only).

We ran three RepeatModeler runs on each of the base genome assemblies (including the unplaced contigs). The resulting consensus sequences from the three species were then combined with curated families from Dfam v3.7 to form a single library.

To remove redundancy in the resulting library, we used Blastn v2.11.0+ and compared the library against itself with a word size of 20 and a minimum percentage identity of 95%. Overlapping sequences were either joined to form a new consensus or one of them shortened to remove the overlap. This was run iteratively until there were no more segments to remove. RepeatMasker was run on each genome with this reduced repeat library, which was further refined by removing portions of each sequence in the library which only aligned to the genomes once. Finally, RepeatMasker was run on each assembly using the library from the secondary refinement.

Identification of structural variants

The assembled genomes of *C. elongatoides* and *C. tanaitica* were mapped to the *C. taenia* reference genome using minimap2 v2.24 [66] (parameters -ax asm10 --eqx). Homology between species was analysed using dot plots generated by D-Genies [67]. SyRI [68] was used to distinguish syntenic and rearranged blocks and to identify SVs (fusion, fissions,

translocations, inversions and duplications). SyRI was run independently for *C. elongatoides* and *C. tanaitica*, both analyses used the *C. taenia* genome assembly as reference. To meet the requirement of having the same number of chromosomes for all species (a requirement by SyRI identification software), Ch01A and Ch01B of *C. elongatoides* and *C. tanaitica* were combined using a 1 Kbp long spacer prior to the analyses. The coordinates of the identified structures were then transferred back to Ch01A and Ch01B.

Due to SyRI producing many short tandem events for translocations and duplications rather than one long rearrangement, all neighbouring blocks of length over 5 Kbp of the same structure type and orientation were merged to be considered a single rearrangement. This approach results in a more parsimonious set of changes in chromosomal structure.

Syntenic blocks and SVs were visualised using NGenomeSyn [69]. For visualization purposes, only structures spanning more than 5 Kbp were considered. Gene synteny analysis was produced using python JCVI package v1.4.16 [70]. Intersections of repeat annotations and indels were produced using Bedtools v2.31 [71].

Sex Chromosome identification and validation through candidate loci PCR

Four pooled DNA samples for *C. elongatoides* and *C. taenia*, along with six individual *C. tanaitica* samples were aligned to their respective reference genomes using bwa v0.7.17 (Li, 2013). Pooled DNA samples were split according to sex and geographic origin of the samples, i.e., two rough geographic groupings split into two sexes resulting in the 4 pools for *C. elongatoides* (altogether 25 males and 45 females) and *C. taenia* (altogether 20 males and 32 females) respectively (see Figure 1 and Supplementary Table S1). SNPs were called from the aligned data using Gatk v4.2.3.0 [72]. Samtools v1.19.2 [40], Vcftools v0.1.16 [73] and Bedtools v2.31.1 [74] were used to calculate the coverage across each genome assembly and the concentration of sex-specific SNPs.

Putative Y-chromosome specific regions were identified in the *C. elongatoides* and *C. taenia* genomes, where pooled female reads had zero coverage and pooled male reads had at least 30% of their average genomic coverage. Putative X-chromosome specific regions

were identified in *C. elongatoides* where the pooled female reads had twice the coverage as pooled male reads and within 20% of the average genomic coverage. Primers were designed to these regions, which included sections of Ch01A in *C. elongatoides,* as well as Ch02 and Ch05 in *C. taenia*. In the case of *C. elongatoides*, where the identified Y-chromosome regions were larger and more numerous, PCR primers were designed with their entire range within the male specific regions. In *C. taenia*, due to a smaller relevant region, only one primer from each pair was designed within the male specific region, while the other primer was placed on the flanking regions. In total, we tested seven *C. elongatoides* X-specific primer sets, twelve *C. elongatoides* Y-specific primer sets, and eighteen *C. taenia* Y-specific primer sets (Supplementary Table S2). To evaluate their efficacy and specificity, we conducted PCR reactions (see Supplementary Table S3 for conditions) with DNA from males and females of both species (see Supplementary Table S1 for details on the individuals used). Additionally, we tested *C. taenia* sex chromosome-specific markers on genomic DNA from six males and three females of *C. tanaitica*, and did not detect any positive bands. Gel electrophoresis was performed after the PCR to confirm the amplification of single products of the expected size and to verify that the bands only appeared in expected individuals. Detailed information of three confirmed primer sets can be found in Table 1.

Candidate Sex-determining genes

To identify potential candidate sex-determining genes in the three *Cobitis* species, we compiled a list of candidate actinopterygian master sex-determining genes based on the literature (see Supplementary Table S4). The presence and genomic location of these genes were then determined using the gene annotation of each species and verified with Tblastn (see Supplementary Table S5).

Cytogenetics

Mitotic and meiotic chromosome preparation

In order to visualise chromosomal pairing, adult *C. elongatoides*, *C. tanaitica,* and *C. taenia* males and females as well as *C. elongatoides* x *C. taenia* hybrid males were injected with 0.1% colchicine solution (1 ml/100 g of body weight). Mitotic and meiotic metaphase chromosome spreads were obtained from kidneys and testes according to previously published protocols [30,75]. Briefly, kidneys and testes were removed, dissected in 0.075 M KCl to release cells and treated hypotonically for 30 min at room temperature. After centrifugation, cells were fixed in freshly prepared fixative methanol: acetic acid (3:1) and washed twice in a new portion of fixative. The fixed cell suspension was then dropped onto slides. Mitotic and meiotic metaphase chromosomes were initially stained with Giemsa to assess chromosomal number and morphology.

Single chromosome Oligo-FISH probe design and Chromosomal painting

Oligomers specific to chromosomes 5 (Ch05) and 20 (Ch20) as well as for the long (Ch01A) and short (Ch01B) arms of chromosome 1 were designed based on the *C. taenia* assembly using Chorus software [76]. Considering the different chromosome lengths, one set of 27,000 oligomers (45-mers) was designed to visualise the whole length of Ch20, while partial regions of chromosome scaffolds Ch01A, Ch01B, and Ch05 were targeted by designed sets of oligomers specific to ensure sufficient probe coverage (Supplementary Table S6). Chromosome regions with very low oligo densities were omitted in the final probe datasets (Supplementary Table S6), which are available upon request. The final probe sets were synthesised as myTAGs® Labelled Libraries (Daicel Arbor Bioscience) and directly used for FISH experiments.

Oligoprobes to Ch20 and Ch01A were labelled with biotin, and oligoprobes to Ch05 and Ch01B were labelled with digoxigenin. Prior to hybridisation, chromosome slides were incubated with 0.01% pepsin/0.01 M HCl at room temperature for 10 min and fixed with 2% paraformaldehyde for 10 min. For two-colour FISH we mixed oligoprobes to Ch05 and Ch20 or Ch01A and Ch01B (50 ng of each probe per slide) with 20 ul of hybridisation mixture (50% formamide, 10% dextran sulphate, 2× SSC, and 500 ng of salmon sperm DNA (Sigma-

Aldrich). Probes were denatured at 86°C in the heating block for 10 min and then put on ice. Slides with mitotic or meiotic chromosomes were denatured in 75% formamide at 74°C for 5 min, dehydrated in an ice-cold series of ethanol (70%, 80%, 96%) and dried prior to denatured probe application. After hybridisation overnight at room temperature, slides were washed three times with 0.2x SSC for 5 min at 42°C and 2× SSC for 5 min at room temperature. The biotin and digoxigenin labelled probes were detected using streptavidin-AlexaFluor 488 (Invitrogen) and anti-digoxigenin-rhodamine (Invitrogen), respectively. After washings in 4× SSC with 0.1% Tween at 44°C for 5 min with shaking, slides were dehydrated in ethanol series (70%, 80% 96%), air dried, and mounted in Vectashield medium containing DAPI (1.5 mg/ml) (Vector).

Wide-field and fluorescence microscopy

Mitotic and meiotic chromosomes with chromosomal painting were inspected using Carl Zeiss Axio Imager.Z2 and Provis AX70 Olympus microscopes equipped with standard fluorescence filter sets. Microphotographs of chromosomes were captured by a CCD camera (DP30W Olympus) using Olympus Acquisition Software and CoolCube 1 using the MetaSystems platform for automatic search, capture and image processing. Microphotographs were finally adjusted and arranged in Adobe Photoshop, CS6 software.

Results

Genome assembly

The assembly process involved multiple sequencing technologies and scaffolding techniques to achieve a high-quality genome assembly. This was accomplished by combining deep coverage with short reads and lower coverage with long ONT reads. The initially fragmented assembly (N50 ~150 Kbp) was subsequently used to generate superscaffolds with Hi-C contact reads (Figure 2A, Table 2, see the Supplementary Table S7 for comparison of assemblies before and after Hi-C interaction mapping).

To generate high-quality genome assemblies (N50 > 40 Mbp), Hi-C contact reads were used to scaffold the initial fragmented genomes (N50 ~150 Kbp) (Supplementary Table S7) into chromosome-level superscaffolds (Supplementary Table S8). A total of 416 (*C. taenia),* 801 (*C. tanaitica*), and 988 million read pairs *(C. elongatoides)* of Hi-C reads were employed to assemble each genome using the Juicer-3D-DNA pipeline (see methods). After filtering, a total of 109 million (*C. taenia)*, 427 million (*C. tanaitica)* and 433 million *(C. elongatoides)* unique contacts were used to assemble each species genome (Supplementary Table S9).

Ultimately, we successfully generated chromosome-level assemblies for males of the three *Cobitis* species: *C. taenia* (n=24, where "n" is the number of chromosomes in a haploid set), *C. tanaitica* (n=25), and *C. elongatoides* (n=25); Table 2, Figure 2A) with N50 values greater than 40 Mbp, indicating high-quality and well-scaffolded genomes. The final genome sizes were 1.6 Gbp for *C. taenia*, 1.7 Gbp for *C. tanaitica*, and 1.8 Gbp for *C. elongatoides*. The majority of each genome, namely 85.8% in *C. taenia*, 69.8% in *C. tanaitica* and 68.4% in *C. elongatoides*, was organised into chromosome-level superscaffolds corresponding in number to the described diploid numbers (2n=48 in *C. taenia* and 2n=50 in *C. elongatoides* and *C. tanaitica*) [77].

Chromosome-level scaffolds were named based on their length in the *C. taenia* genome from Ch01 (the largest) to Ch24 (the smallest) (Supplementary Table S10). This suggested chromosome nomenclature is based on chromosome scaffold size and does not correspond to previously published classifications based on chromosome morphology (e.g. [75,77]). For the other two species, we followed the same naming convention and orientation for inferred homologous chromosomes. In the case of Ch01, which represents a recently fused chromosome in *C. taenia*, we labelled the two homologous chromosomes in *C. elongatoides* and *C. tanaitica*, which represent the ancestral syntenies, as Ch01A and Ch01B.

A/B compartments and topologically associated domains

Comparison of Hi-C matrices revealed similar patterns of chromosomal interactions amongst the species (Figure 2). The detection of 3D structures, such as compartments and

(TADs), showed consistent patterns in the three species (Figure 2A), mirroring previous observations in other vertebrates [45,78]. The genome-wide distribution of A/B compartments was similar across taxa, with ≈50% of the genome was detected as A compartments ("open" chromatin), and no major differences were observed on compartment strength between species (Figure 2B). TADs exhibited the same trends. In the three species, TADs of ≈0.8 Mbp (Supplementary Table S11) were defined with equal insulation capacity (Figure 2C, 2D). Overall, our results suggest a high level of chromatin structure conservation among *Cobitis* species.

Gene annotation

For *C. taenia* we identified 31,513 genes. BUSCO analysis using actinopterygii_odb10 (3,640 BUSCOs) showed 95.6% (C:93.3%; F:2.3%) coverage (BUSCO v3.0.2, ODB v10, hmmsearch v3.4). Both other genomes give slightly worse results, possibly due to less contigs placed in chromosome-level scaffolds, with *C. elongatoides* having 42,868 genes with a BUSCO coverage of 93.8% (C:88.6%; F:5.2%) and *C. tanaitica* having 40,250 genes and a BUSCO coverage of 95.0% (C:90.9%; F:4.1%). The number of genes per chromosome and the proportion of scaffolded versus unscaffolded parts of the genome are given in Supplementary Table S10.

Repetitive element annotation

Overall, approximately 54-55 % of the assembled *Cobitis* genomes were identified as being repetitive elements. This includes 9,928 repeat classes and 90 repeat families in *C. taenia*, 9,882 repeat classes and 90 repeat families in *C. elongatoides*, and 9,924 repeat classes and 91 repeat families in *C. tanaitica*, excluding simple, low complexity, and satellite repeats.

Most families are equally distributed (Figure 4A), which suggests they expanded before speciation, and they are no longer active. But several families are expanded only in

one or two species, suggesting that they were active after speciation. These families could therefore be used in the future for further detection of deregulation in hybrids.

A Kimura distance-based copy divergence analysis demonstrated that the closely related *C. taenia* and *C. tanaitica* have very similar TE content with clear evidence of an on-going expansion in some families, while *C. elongatoides* differs from both with indications of a recent decline in TE activity (Figure 4B).

Inference of homology and structural variants

Assembled genomes were aligned to identify homologous chromosomes between the species. A clear one-to-one alignment pattern was observed along the entire chromosomal length for 23 out of the 24 (in *C. taenia*) or 25 (in *C. tanaitica* and *C. elongatoides*) chromosomes (Figure 3A, 3B, Supplementary Figure S1, S2). The mapping of the remaining chromosome (Chr01) of *C. taenia* was split between the two remaining chromosomes (Chr01A and Chr01B) of *C. tanaitica* and *C. elongatoides* indicating a chromosomal fusion in *C. taenia*.

Syntenic regions and chromosomal rearrangements were identified using SyRI, followed by a merging procedure to ensure parsimony (see Methods). The proportion of syntenic regions between homologous chromosomes ranged from 24 to 68% in the comparison of *C. tanaitica* to *C. taenia* and from 17 to 49% in the comparison of *C. elongatoides* to *C. taenia*. We identified a total of 35,633 structural variants in the comparison of the *C. tanaitica* genome to the *C. taenia* genome (*C. taenia* - *C. tanaitica*) and 41,228 variants in the comparison of *C. elongatoides* to the *C. taenia* (*C. taenia* - *C. elongatoides*) (Supplementary Table S12). Among the longest rearrangements, we detected 53 and 66 inversions spanning over 1 Mbp in the *C. taenia* - *C. tanaitica* and *C. taenia* - *C. elongatoides* comparisons, respectively. Other types of rearrangements, such as duplications and translocations, were often scattered as adjacent shorter events, which could be better explained by a single larger event. To address this, we applied our parsimony merging procedure (see Methods), resulting in a total of 69 rearrangements in the *C. taenia* - *C. tanaitica* comparison, and 116 rearrangements in the *C. taenia* - *C. elongatoides* comparison,

each spanning over 1 Mbp (Supplementary Figure S2, Supplementary Table S13). These extensive rearrangements include large translocations in Ch11 and Ch04 in *C. tanaitica*, with approximately one-third of the chromosome spanned by inverted translocations (Figure 3B).

Identified insertions and deletions spanned a total length of 7.6 Mbp and 2.8 Mbp in the *C. taenia* - *C. tanaitica* and *C. taenia* - *C. elongatoides* comparisons respectively. Of these indels, approximately 73% were composed of repeats in the *C. taenia* - *C. tanaitica* comparison and 71% in the *C. taenia* - *C. elongatoides* comparison. Among the most represented classes of repeats among the indels were DNA hAT-Ac elements (9% in *C. taenia* - *C. tanaitica*, 4.8% in *C. taenia* - *C. elongatoides*) and long terminal repeat (LTR) Gypsy elements (10.6% in *C. taenia* - *C. tanaitica*, 12.4% in *C. taenia* - *C. elongatoides*).

The intrachromosomal rearrangements we detected were confirmed using gene synteny analysis (Figure 3A). Additionally, we observed several interchromosomal events, 12 in the *C. taenia* - *C. tanaitica* comparison, 50 in the *C. taenia* - *C. elongatoides* comparison and 100 in the *C. tanaitica* - *C. elongatoides* comparison. These interchromosomal events span on average 8 genes in *C. taenia* - *C. tanaitica*, 11 genes in *C. taenia* - *C. elongatoides* and approximately 9 genes in *C. tanaitica* - *C. elongatoides*. The longest detected rearrangements (spanning over 40 genes) from the *C. taenia* - *C. elongatoides* comparison are located on Ch05, Ch19, Ch22, and Ch23 in *C. elongatoides* and Ch02, Ch03, Ch10, and Ch22 in *C. taenia*. Interchromosomal rearrangements between these pairs of chromosomes are also the longest ones in the *C. tanaitica* - *C. elongatoides* comparison.

Identification of sex chromosomes

Pooled genomic sequencing from 24 males and 45 females from *C. elongatoides* and 20 males and 32 females from *C. taenia* were used to calculate sex-specific coverage and identify sex-associated SNPs across each respective reference genome. Due to the difficulty of getting *C. tanaitica* individuals, three males and three females were sequenced individually and used for the analysis.

In *C. elongatoides*, our analysis provided a clear signal across the whole Ch01A scaffold. Portions of this chromosome-level scaffold in *C. elongatoides* had a coverage in females equal to the average genomic coverage and half the coverage in males, consistent with an X chromosome, while other regions of the chromosome showed half the average genomic coverage in males and low coverage in females, consistent with a Y chromosome (Figure 5C). This intermixed pattern suggests that *C. elongatoides* has an X/Y system and that the assembled Ch01A scaffold represents a chimeric combination of these chromosomes' sequence.

By contrast, no visible coverage differences were observed in any chromosome in *C. taenia* and *C. tanaitica* (Figure 5A, 5B), suggesting that these species have, compared to *C. elongatoides*, undifferentiated sex chromosomes, if any.

Sex-specific SNPs were identified in all three species using the (pooled) Illumina datasets. The proportion of these sex-specific SNPs was used to locate regions of differentiation between male and female individuals. This revealed several potential sex-specific regions in *C. tanaitica*, most notably a 900 Kbp region on Chromosome 6 (Figure 5E) as well as once again highlighting the whole of Chromosome 1A in *C. elongatoides* (Figure 5F). No high concentration of sex-specific SNPs was observed in *C. taenia* (Figure 5D).

As a final attempt to identify a sex chromosome in *C. taenia*, regions were identified with zero coverage in females and a coverage greater than 30% of the average genomic coverage in males (where the Y chromosome-specific regions are expected to have an average of 50% coverage). The two regions with the highest density of such regions were located on Ch02 and Ch05. PCR primers were designed to these two chromosomes such that one of the two primer pairs was within one of these putative male specific regions. The sex-chromosome specificity of primers designed to Ch05 was successfully validated using an additional 11 sexed individuals not used in the Pool-Seq data, while the Ch02 primers failed to predict the sex of the tested samples (Supplementary Table S2).

Primers were also designed to amplify regions predicted to be sex chromosome-specific (either X- or Y-specific) in *C. elongatoides* to use for future sex identification of pure

individuals or identification of sex chromosome presence in interspecific hybrids. The PCR primers and their amplification conditions were tested on the individuals that were not used in the creation of the PoolSeq library. As a criterion for primer selection for further screening we considered that primers were (1) amplifying a single band in a sex-chromosome-specific manner (i.e. Y-marker in males only and X-marker in both sexes), and (2) they amplified in a species-specific manner, hence, not making any product in non-target species. Finally, three sets of primers were selected, reliably diagnosing Ch01A Y- and X- specific loci in *C. elongatoides* and Ch05 Y-specific loci in *C. taenia* (Table 1). A primer set that exclusively amplified the *C. taenia* X locus was not found.

From the literature (Supplementary Table S4), 41 genes (including paralogs) were found which are master sex determination or key regulators of sex-determining pathways in actinopterygians. All of these genes were mapped to chromosome-level scaffolds except four in *C. tanaitica* and nine in *C. elongatoides*, which were found on unplaced scaffolds. Notably, among these genes, *paics* (the master sex determination genes in the blue tilapia - *Oreochromis aureus*) aligned to Ch01A, the putative sex chromosome, in *C. elongatoides* (Supplementary Table S5).

Additionally, we analysed the list of genes located on Ch01A to check for the presence of any additional genes, which were considered as linked to sex differentiation in various fish species (Supplementary Table S5). We detected the presence of four such genes (*bmp2b, gata4, gpatch2,* and *gopc*) which are known to play a key role in vertebrate sex determination/differentiation and thus are potential candidates for the sex determination master gene in *Cobitis elongatoides*. Interestingly, *gata4* and *gpatch2* are located in a highly differentiated region of the X chromosome while *gopc* and *bmp2b* are in a moderately diverged region. *paics* is in the least diverged part. All these genes are located on the same syntenic group (autosome 20) in *Danio rerio*.

Cytogenetic Validation of Chromosome Structures Through Mitotic Chromosome Painting

Chromosome painting was performed using probes designed based on the genome assembly of *C. taenia*. These included probes covering the entire scaffold Ch20, a part of the of Ch05, as well as two regions of Ch01 corresponding to collinear parts of Ch01A and Ch01B of *C. tanaitica* and *C. elongatoides*. These paintings confirmed the accuracy of our genome assemblies across four selected linkage groups in all three sexual *Cobitis* species.

Specifically, the probe of Ch05 highlighted the distal part of the q-arm of a large submetacentric chromosome across all species, confirming the structural integrity and assembly accuracy of this chromosome scaffold. The probe of Ch20 labelled the q-arm of small subtelocentric chromosomes in *C. taenia* and *C. elongatoides*, suggesting a conserved structure in these species. Conversely, in *C. tanaitica*, the probe signal was observed in the q-arm of a small acrocentric chromosome (Figure 6), suggesting morphological differences. Finally, the application of probes for linkage groups Ch01A and Ch01B in *C. elongatoides*, when applied in *C. taenia*, showcased distinct hybridisation signals on the short and long arms of the largest metacentric chromosome with its centromeric region unstained and exhibiting only DAPI signal (Figure 6). This confirmed a fusion event unique to this species. In *C. tanaitica* the Ch01A and Ch01B probes highlighted two pairs of acrocentric chromosomes, while in *C. elongatoides*, two pairs of submetacentric chromosomes, once again highlighting structural differences among the species.

Interestingly, even after the visualisation of Ch01A, we did not observe any difference in morphology and hybridisation signals between male and female mitotic chromosomes in all three studied sexual species, despite the possible role of Ch01A in sex determination of *C. elongatoides* and the identified divergence between Y- and X-specific sequences. This suggests that differentiation of the XY chromosomes is limited to nucleotide substitutions and other small rearrangements rather than large structural changes.

In diploid *C. elongatoides-taenia* (ET) hybrids, chromosome painting verified the presence of orthologous Ch05 and Ch20 corresponding to those identified in *C. elongatoides* and *C. taenia*. Further, the Ch01A and Ch01B probes revealed the fused *C. taenia*'s

chromosome Ch01 alongside the distinct submetacentric chromosomes from *C. elongatoides* (Figure 6).

Cytogenetic Exploration of Meiotic Chromosome Spreads in Hybrids and Pure Species

Chromosome painting with Ch05 and Ch20 probes applied to meiotic metaphase I spermatocyte spreads showed, in all three parental species, the presence of one larger bivalent corresponding to Ch05 homologs and a smaller bivalent corresponding to Ch20 homologs (Supplementary Figure S3). In addition, meiotic metaphase I of *C. taenia* showed both Ch01A and Ch01B hybridisation signals on the single largest bivalent, while *C. elongatoides* and *C. tanaitica* exhibited two distinct small bivalents corresponding to Ch01A and Ch01B paired homologs, corroborating the mitotic chromosome data (Supplementary Figure S3).

To understand how the pairing patterns proceed in interspecific hybrids, we applied these chromosome-specific probes to 526 meiotic metaphase I spermatocytes from two diploid *C. elongatoides-taenia* (ET) hybrid males (Figure 7A-7C). These males were derived from crossing of *C. taenia* mothers and *C. elongatoides* fathers and were now confirmed to have inherited the Y gametolog of Ch01A from *C. elongatoides* via positive PCR amplification signal with the Y-linked primer pair and negative amplification for the X-linked primer pair (see Table 1).

Despite differences in the total number of metaphases inspected for each individual and chromosome (see Figure 7D for exact counts), statistical analysis revealed significant differences in pairing success among the four investigated chromosomes. A generalised linear model (GLM) with a binomial error structure was used to test the effects of chromosome identity, individual, and their interaction on pairing success (response variable: paired vs. unpaired). The model showed that chromosome identity had a highly significant effect on pairing success ($p < 0.0001$), indicating consistent interchromosomal differences in pairing likelihood.

Post-hoc pairwise comparisons, adjusted for multiple testing using the Bonferroni correction, confirmed that Ch01B paired significantly more frequently than all other chromosomes ($p < 0.0001$ for all pairwise contrasts; Figure 7D). Ch05 and Ch20 bivalents were observed in only a small proportion of cells, with both chromosomes showing significantly lower pairing likelihoods compared to Ch01B ($p < 0.0001$ for both comparisons). Ch01A, the putative Y gametolog derived from *C. elongatoide*s, exhibited the lowest pairing rates overall, appearing primarily as univalents (Figure 7D).

While these interchromosomal differences were consistent across both individuals, significant interindividual differences were observed for Ch01B. A separate GLM for Ch01B revealed that its pairing success was significantly lower in one hybrid male (*et1c5*) compared to the other (*et1c4*; $p = 0.0006$). However, no significant interindividual differences were detected for the other chromosomes, suggesting that variability in pairing dynamics for Ch01B might reflect unique characteristics of this chromosome or its interaction with individual-specific factors.

These findings underscore the variability in chromosomal pairing success, with Ch01B showing the greatest likelihood to form bivalents, while the Y gametolog of Ch01A from *C. elongatoides* rarely pairs with its ortholog from *C. taenia*. This variability likely contributes to the observed chromosomal incompatibility and reproductive barriers in these hybrids.

Discussion

Filling the taxonomical gap in chromosome level assemblies

Recent advances in long read sequencing and chromatin capture technologies have substantially improved the feasibility of chromosome-level genome assemblies for non-model organisms. We took advantage of this trend to generate three high quality *Cobitis* genome assemblies. While assemblies created with these technologies represent a major improvement over short read assemblies, they can still fail in assembling genomic regions enriched in repetitive content (e.g. centromeres, telomeres), and structural variants. This can

lead to fragmented scaffolds that are not placed in their respective chromosomes [79,80]. In this respect, we were unable to confidently place 14-32% of sequences in each genome. Despite this, these assemblies are still of high enough quality to contain the expected number of chromosomes in all three species, with an N50 range of 43-53 Mbp.

We used the genome assemblies to design 4 chromosome-specific probes (Ch01A, Ch01B, Ch05, and Ch20). Their application to metaphase spreads of the three species confirmed some features of the assemblies, most notably the fusion of two chromosomes (Ch01A and Ch01B) into a single chromosome (Ch01) in *C. taenia*. Interestingly, however, while both Ch01A, Ch01B appear as small chromosomes in *C. elongatoides* and *C. tanaitica*, they are not acrocentric, but submetacentric, with p-arms highlighted by a euchromatic probe in both species. This in turn suggests that it was not a standard Robertsonian fusion into Ch01 of *C. taenia,* but also some fine-scale structural rearrangements took place. This is in line with suggested inversions and translocations along both Ch01 - Ch01A and Ch01B (see and compare Figure 6 and Figure 3A, 3B).

Our comparison to published chromosome-level assemblies suggests that the syntenic groups of the Cobitoidea suborder appear highly conserved, with most previously studied species demonstrating 25 elements in haploid state and very few interchromosomal rearrangements. However, fusions of two elements have been identified in the genome assemblies of *Paramisgurnus dabryanus* (2n=48, [81] and *Oreonectes platycephalus* (2n=48, [82]). A closer investigation of these fused chromosomes reveals their independent origin from the fusion observed in *C. taenia;* in *P. dabryanus* the fusion involved homologs of *Cobitis* Ch14 and Ch22 (Figure 3A), while in *O. platycephalus* - homologs of *Cobitis* Ch21 and Ch22 (Supplementary Table S14).

While the number of chromosome-level genome assemblies from non-model species has grown exponentially, the data remain taxonomically and geographically biased. This bias is evident in Cypriniformes, a diverse and economically significant order of Old-World freshwater fishes. As of March 2025, the NCBI Genome database lists 306 genomes of Cypriniformes, including 89 genomes from species of the family Cyprinidae (which has around

1,780 species in total) and only 5 species from the family Cobitidae (which has approximately 260 species). This is even though the Cobitidae family, which diverged from its common ancestor ~20–30 million years ago (Mya) [83], spans the entire Palearctic region. This bias likely stems from the economic importance of the cyprinids, particularly carps, which are farmed extensively worldwide. Loaches, on the other hand, are generally non-commercial, with exceptions in China and Japan, explaining why the available genomes primarily originate from East Asian species.

Such a taxonomic and geographic imbalance is understandable given the costs and labour required for high-quality chromosome-level assemblies. However, it significantly limits large-scale comparative genomic studies by underrepresenting or omitting entire deeply diverged clades. In this context, the present study, delivering chromosome-level genomes of *Cobitis taenia*, *C. tanaitica*, and *C. elongatoides*, more than doubles the number of available Cobitidae genomes. Crucially, it addresses a key taxonomic and geographic gap by providing genomes from the Western Cobitidae lineage [84], which diverged from the nearest available Asian *Misgurnini* species during the Oligocene epoch (~30 Mya).

Beyond merely filling this gap, our assemblies—supported by additional work on Pool-Seq and chromosome-specific probe design—have leveraged the unique potential of this dataset to advance our understanding of the intricate links between speciation, hybridisation, asexuality, and hybrid sterility at two distinct levels. First, they enabled the detection and characterization of sex chromosomes in these closely related but reproductively divergent hybridising species. Second, they facilitated a deeper understanding of the mechanisms underpinning chromosomal incompatibilities in hybrids.

Structural variant evolution

Inversions are structural mutations well known for suppressing recombination in heterozygous states, thereby creating barriers to genetic exchange between chromosomes within populations [85]. A striking feature of *Cobitis* genome evolution is the prevalence of intrachromosomal structural rearrangements over interchromosomal changes, including

numerous pericentric and paracentric inversions. Even between the recently diverged species *C. taenia* and *C. tanaitica* (0.5–1.5 Mya), we identified over 50 large (over 1 Mb) inversions (Figure 3A, Supplementary Table S8, S9), including pericentric ones on Ch01a and Ch01b, confirmed by changes in centromere position from the cytogenetic methods (Figure 6).

Surprisingly, the number of large interchromosomal translocations remains low, limited to a single Ch01a/01b fusion, apomorphic to *C. taenia* as discussed above. The distribution of inversions appears non-random across chromosomes: while some chromosomes (e.g. Ch15, Ch16, Ch21, Ch24) maintain relatively conserved synteny, others (e.g. Ch01b, Ch4, Ch10) exhibit extensive reshuffling (Figure 3; Supplementary Figure S1). This chromosomal reshuffling may influence meiotic pairing frequencies and bivalent formation in hybrids.

Recent studies have demonstrated that inversions tend to fix more frequently in population zones where reproductively isolated species overlap [86]. In such cases, inversions may be positively selected as they preserve loci's synteny by suppressing recombination in hybrids. Our observations align with this hypothesis. Future pan-genomic projects will target the distribution and population structure of inversions within *Cobitis* species and explore their role in gonosome divergence.

Repeatome evolution

As in other cypriniform fishes [87], *Cobitis* appears to have a relatively high transposable element (TE) content, which likely explains the large genome size in this group (~1.7 Gbp in *Cobitis* vs ~500-700 Mbp in different species of *Triplophysa* [88] and ~450 Mbp in *Beaufortia* [89]). Representatives of both Class I and Class II TEs are almost equally dominant and continue to expand. Similar to other fish genomes, *Cobitis* species are poor in SINEs [90]. However, like cyprinids [87], *Cobitis* exhibits a high level of TE diversity.

The *Cobitis* genome has a relatively high LTR content (17-19%) compared to other teleosts, including *Danio rerio* (5%) [87] and *Paramisgurnus dabryanus* (7.5%) [81]. Notably, *C. taenia* shows evidence of a recent transpositional burst involving DNA transposons (hAT), LTR retroelements (Gypsy), and long interspersed nuclear element (LINE) retrotransposons

(L2). Compared to many other teleosts, *Cobitis* appears to be undergoing an ongoing transposon expansion, where the rate of active transposon accumulation surpasses the rate of their decline. Even in *C. elongatoides*, with a recent decline in activity, this is still true.

Bursts of TEs have been suggested to play an important role in population diversification and speciation [91–93], and the high TE activity observed in *Cobitis* aligns well with this hypothesis, assuming a contribution of TE to high number of genomic structural variants, rapid karyotype evolution as well as speciation in this group.

Hybridising species have nonhomologous genetic sex determination systems

It has also been observed that mechanisms of sex determination, while being of fundamental importance for species, evolves far more dynamically than previously believed [94,95]. The currently available TreeOfSex database v.1 [96] lists 40 cypriniformes species for which sex determination has been investigated. Of these only three previous studies suggested the existence of genetic sex determination (GSD) in four species across the entire Cobitoidei suborder, namely X0 [97], ZW [98] and multiple sex chromosomes X_1X_2Y [99]. Additionally, two recent papers [81,82] indicated the presence of sex chromosomes in two Asian loaches, an XY system in *Oreonectes platycephalus* and a ZW system in *Paramisgurnus dabryanus*, respectively.

Our study brings robust evidence for the presence of GSD in this group of fish, clearly identifying candidate genomic regions/chromosomes associated with sex in each species and validating these predictions with PCR primers in two of the species, which enables further comparative studies within this dynamically evolving field of research. Using Pool-Seq of dozens of male and female individuals in two species and individual male and female sequencing for the third species our study revealed dynamic sex-chromosome turnover within *Cobitis sensu stricto*, namely no signal (*C. taenia*) or ambiguous signal (*C. tanaitica*) on two independent linkage groups, while relatively well differentiated XY chromosomes in linkage group Ch01A of *C. elongatoides*, which contains a gene previously associated with GSD in the blue tilapia and four additional genes regulating the sex determination cascade in

vertebrates (Supplementary Table S5). This suggests a rapid turnover of sex chromosomes within the lineage which diverged less than 10 Mya (divergence of *C. elongatoides* from the other species) or even more recently, if we consider that *C. taenia* and C. *tanaitica* diverged in the last 2 Mya [27] and appear to have different sex chromosomes (Ch05 and Ch06 respectively).

The observed turnover of sex chromosomes among closely related *Cobitis* species aligns with the well-documented evolutionary plasticity of GSD systems in teleost fishes [94,95]. Moreover, the sex chromosomes identified in the other cobitoid species (XY in *Oreonectes platycephalus* and ZW in *Paramisgurnus dabryanus*) originated from different syntenic groups (Supplementary Table S14).

However, our finding that *C. elongatoides* utilises a GSD system (XY on Ch01A) that is non-homologous to the putative sex-determining regions in *C. tanaitica* (XY on Ch06) and *C. taenia* (XY on Ch05) adds a significant dimension to understanding the inherent link between interspecific hybridisation and the evolution of asexuality and polyploidy as hybridisation between *C. elongatoides* and the other two species consistently produces sterile males and fertile, clonally reproducing females. Such asymmetric patterns are not unique to *Cobitis* but rather have been consistently reported across diverse vertebrate taxa following hybridisation events and have long suggested a potential role for sex chromosomes in linking hybrid sterility and asexuality [20]. Interestingly, in most studied systems involving asexual hybrids, sex chromosomes remained undifferentiated, unknown, or poorly characterised, leaving this hypothesis largely speculative until now [20]. However, among those asexual systems with known sex chromosomes, the ZZ/ZW prevails over XX/XY [20].

While such asymmetries superficially align with Haldane's Rule, especially given current evidence for male heterogamety in loaches, the fertility of hybrid females is not maintained through typical meiotic repair mechanisms but arises via PMER — a cellular mechanism where germ cells duplicate their chromosomes before meiosis, allowing bivalents to form between identical chromosomal copies. This bypasses meiotic pairing issues and restores fertility. Crucially, however, PMER is restricted to female hybrids, which subsequently

reproduce clonally, while hybrid males remain sterile. Notably, transplantation experiments have demonstrated that PMER can be reactivated in spermatogonial cells of hybrid males if these cells develop in a female gonadal environment and transdifferentiate into oogonia that may subsequently give rise to unreduced oocytes [21]. While such a finding suggests that the ability to initiate asexual reproduction via PMER is primarily dictated by tissue-specific cues from the female gonadal environment, our discovery adds a new layer of complexity to this. It demonstrates that GSD systems in hybrids represent a combination of fundamentally different mechanisms inherited from parental species. Such an interplay between GSD and the female gonadal environment hints at a deeper integration between genetic triggers, epigenetic regulation, and cellular signalling pathways in enabling PMER. It raises a hypothesis that, while the female-specific gonadal environment acts as a permissive factor for PMER, the genetic sex determination system may function as an upstream regulatory mechanism, shaping how these cellular pathways are activated in hybrids. Our findings, therefore, highlight an exciting new research avenue into the genetic and molecular basis of PMER and the broader evolutionary consequences of sex chromosome turnover in hybrid systems.

Chromosome Pairing in Hybrids: Insights into Hybrid Sterility and Asexuality

The assembly of chromosome linkage groups and the design of chromosome-specific probes allowed us to investigate meiotic chromosome pairing in hybrid males, providing unprecedented insights into the chromosomal basis of hybrid sterility and asexual reproduction. Such analyses, examining the pairing rates of individual chromosomes in hybrid meiosis, remain technologically challenging and are rarely performed. Yet, they address a fundamental aspect of hybrid sterility models, which suggest that individual chromosomes vary in their contributions to meiotic success or failure [100,101]. Our findings align with this hypothesis, showing that orthologous chromosome pairs (Ch01A, Ch01B, Ch05, and Ch20) exhibit significantly different rates of bivalent formation in hybrid males (Figure 7D). For example, while two-thirds of spermatocytes contained a bivalent between *C. elongatoides*-derived Ch01B and its ortholog from *C. taenia*, only 2 out of > 300 cells displayed pairing

between the Y-linked Ch01A from *C. elongatoides* and its ortholog from *C. taenia*. Analogously, for Ch05 and Ch20, given an average of five bivalents per spermatocyte [18] and assuming random pairing, approximately 25 cells out of 120 and 117 inspected, respectively, would be expected to contain bivalents of these chromosomes. However, far fewer such cells were observed, indicating systematic biases in pairing success. In addition, significant interindividual differences were found between both hybrid males analysed in terms of pairing success of at least one chromosome (Ch01B), suggesting that variability in pairing dynamics might reflect unique characteristics of this chromosome or its interaction with individual-specific factors.

These results, the first of their kind in an asexually reproducing vertebrate, gain additional importance when interpreted within the context of asexual gametogenesis pathways. Studies have shown that, even in evolutionarily successful clones, only a minority of gonial cells undergo PMER, while most hybrid female oogonia and all male spermatogonia fail to pair orthologous chromosomes and stall at the first meiotic checkpoint [18,102]. Despite this widespread failure, each gonocyte still contains several fully formed bivalents, with their numbers varying between individuals and sexes (~5 bivalents in hybrid males and ~16 in hybrid females). This variability raises critical questions about whether specific chromosomes systematically contribute more or less to pairing success and why such differences persist between males and females.

Furthermore, accumulating evidence suggests that asexual hybrids accumulate a loss of heterozygosity [11,23,24,103]. Our results propose an exciting hypothesis: if individual chromosomes differ in their likelihood to form orthologous bivalents during PMER, certain linkage groups may be disproportionately affected by gene conversion events. This non-random pairing propensity could predict non-random distributions of loss of heterozygosity across the genomes of asexual hybrids.

Conclusions

Our study highlights how integrating chromosome-level genome assemblies, molecular cytogenetics, and meiotic analysis can illuminate the mechanisms underlying hybrid sterility and asexuality. We show that hybridising *Cobitis* species differ in their sex determination systems, accumulate extensive structural variants, and exhibit non-random, chromosome-specific pairing affinities during male meiosis. Notably, the frequent mispairing of the *C. elongatoides*-originated Y chromosome Ch01A points to structural or regulatory incompatibilities as potential barriers to normal gametogenesis. Together, these findings underscore how genome divergence between parental species may shape reproductive outcomes in hybrids and pave the way for future research into the chromosomal basis of asexuality.

Additional Files

Supplementary Figure S1. Dotplot analysis of genomic homologies between *Cobitis elongatoides*, *C. taenia*, and *C. tanaitica*. Dotplots depict pairwise genomic comparisons between *C. elongatoides* (E), *C. taenia* (T), and *C. tanaitica* (N), illustrating sequence homology and structural variation. Each panel represents a pairwise alignment: (A) *C. elongatoides* vs. *C. taenia*, (B) *C. taenia* vs. *C. tanaitica* and (C) *C. elongatoides* vs. *C. tanaitica*. Diagonal lines indicate regions of synteny, while disruptions or scattered points suggest structural rearrangements such as inversions, translocations, or duplications. The density and continuity of dot patterns reflect the level of sequence similarity and collinearity between species**.**

Supplementary Figure S2. Synteny plots of homologous sequences and intrachromosomal rearrangements in chromosomes of the three Cobitis species (from top to bottom *C. tanaitica*, *C. taenia* and *C. elongatoides*). Darker colour shades highlight the SyRI detected events while lighter shades show the results of parsimonious merging. Only blocks longer than 5 Kbp are shown.

Supplementary Figure S3. Chromosome painting of Ch05 (green) and Ch20 (red) (A-C) as well as Ch01A (green) and Ch01B (red) (D-E) on meiotic metaphases of *C. taenia* (A, D), *C. tanaitica* (B, E), *C. elongatoides* (C, F). Ch05 and Ch20 (a-c) indicate two bivalents in all species. Chromosome painting of Ch01A and Ch01B indicates one bivalent in *C. taenia* (D) while two bivalents in *C. tanaitica* (E) and *C. elongatoides* (F). Chromosomes are stained by DAPI (blue). Scale bar = 10 μm.

Supplementary Table S1. List of the specimens used in the study, including information on the species and sex of the individuals, the tissues taken from them, together with the purpose and technique of the study and where the specimens were collected.

Supplementary Table S2. List of all primers tested for PCR amplification and the number of tested individuals of *C. elongatoides* and *C. taenia*. Rows coloured in light green are showing primer pairs which gave expected PCR results on both species.

Supplementary Table S3. PCR amplification conditions (valid for all primer combinations).

Supplementary Table S4. List of known master sex-determining genes (MSD), candidate MSD, or those connected with male- or female-developing pathway (sex-related) in actinopterygians. Genes missing from any *Cobitis* annotation file are yellow marked.

Supplementary Table S5. Distribution on chromosomes of three loach species of identified master sex determination genes among Actinopterygians. Confirmed master sex-determining genes are marked as bold.

Supplementary Table S6. Design of oligo probes for chromosome-specific FISH visualisation.

Supplementary Table S7. Pre and post Hi-C assembly statistics.

Supplementary Table S8. Superscaffolds lengths (in bp).

Supplementary Table S9. Hi-C mapping quality stats.

Supplementary Table S10. Number of bases and annotated genes per chromosome.

Supplementary Table S11. Compartments and TADs metrics.

Supplementary Table S12. Counts of structural variants as detected by SyRI reported by

different types and lengths.
Supplementary Table S13. Counts of intrachromosomal rearrangements after the filtering step leaving out the shortest (< 5 Kbp) variants and the merging step combining variants matching in their type, location and orientation.
Supplementary Table S14. Chromosome homologies between cobitoid and a reference cyprinid species (*Danio rerio*) identified in available chromosome level genome assemblies. Sex chromosomes are marked with purple. Fused chromosomes are bold.

List of Abbreviations

Bwa – Burrows-Wheeler Aligner; ET – C. elongatoides-taenia hybrid; GLM – generalised linear model; GSD – genetic sex determination; LINE – long interspersed nuclear element; LTR – long terminal repeat; Mya – million years ago; ONT – Oxford Nanopore Technology; PMER – premeiotic endoreplication; SSC – saline-sodium citrate; SV – structural variant; TAD – topologically associated domain; TE – transposable element.

Ethics Approval

The Valid Animal Use Protocol was in force during the study at the Institute of Animal Physiology and Genetics, Liběchov, Czech Republic (No. CZ 02386). All institutional and national guidelines were covered by the "Valid Animal Use Protocol" No. CZ 02386 of the Laboratory of Fish genetics.

Acknowledgements

Authors are profoundly obliged to our greatest technicians, Š. Pelikanová, J. Machová and P. Šejnohová. We thank M. Schartl and M. Stöck for inspiring advice regarding the PoolSeq analysis and sex chromosome identification.

Author Contributions

K.J. and J.P. conceived and supervised the research. D.D., L.A., T.T., D.K., J.K., A.M., K.J., P.P., P.H. collected the samples and performed the experiments. S.A.S., D.D., V.T., Y.G., K.J., Z.H., M.K., A.R.-H., L.Á.-G., G.P., E.H., L.A., O.B., T.T., M.K.D., A.B., H.K. analysed data. S.A.S., D.D., V.T., K.J., J.P., Z.H., L.Á.-G., L.A., T.T., A.B., R.R., Y.G. interpreted the data and wrote the manuscript. All authors have read and approved the final manuscript.

Funding

The study was supported by the Czech Science Foundation Project No. 24-12217S. Institute of Animal Physiology and Genetics receives support from Institutional Research Concept, Grant/ Award Number: RVO67985904. S.A.S. was supported by the Charles University Research Centre program No. UNCE/24/SCI/006. V.T. was supported by the Marie Skłodowska-Curie Actions - COFUND project, which is co-funded by the European Union (MERIT - Grant Agreement No. 101081195). A.R.-H. is founded by the Spanish Ministry of Science and Innovation (PID2020-112557GB-I00 funded by AEI/10.13039/501100011033), the Agència de Gestió d'Ajuts Universitaris i de Recerca, AGAUR (2021SGR00122) and the Catalan Institution for Research and Advanced Studies (ICREA). L.A.-G. and G.P. were supported by FPI predoctoral fellowships from the Ministry of Economy, Industry, and Competitiveness (PRE-2018-083257 and PRE-C-2021-0083, respectively). Z.H. was supported by the Grant Agency of Charles University (grant number 314222) and SVV 260818/2025.

Data Availability

The raw genomic sequencing data for all analysed species have been deposited in the European Nucleotide Archive (ENA) under BioProject accession number PRJEB90107.

Abbreviations

Bwa – Burrows-Wheeler Aligner; ET – C. elongatoides-taenia hybrid; GLM – generalised linear model; GSD – genetic sex determination; LINE – long interspersed nuclear element; LTR – long terminal repeat; Mya – million years ago; ONT – Oxford Nanopore Technology; PMER – premeiotic endoreplication; SSC – saline-sodium citrate; SV – structural variant; TAD – topologically associated domain; TE – transposable element.

Competing Interests

The authors declare that they have no competing interests.

References

1. Lenormand T, Engelstädter J, Johnston SE, Wijnker E, Haag CR. Evolutionary mysteries in meiosis. *Philos Trans R Soc B Biol Sci*. 2016; doi: 10.1098/rstb.2016.0001.

2. Kochakpour N, Moens PB. Sex-specific crossover patterns in Zebrafish (Danio rerio). *Heredity*. 2008; doi: 10.1038/sj.hdy.6801091.

3. Ortiz-Barrientos D, Engelstädter J, Rieseberg LH. Recombination Rate Evolution and the Origin of Species. *Trends Ecol Evol*. 2016; doi: 10.1016/j.tree.2015.12.016.

4. Thompson MJ, Jiggins CD. Supergenes and their role in evolution. *Heredity*. 2014; doi: 10.1038/hdy.2014.20.

5. Berdan EL, Aubier TG, Cozzolino S, Faria R, Feder JL, Giménez MD, et al. Structural Variants and Speciation: Multiple Processes at Play. *Cold Spring Harb Perspect Biol*. Cold Spring Harbor Laboratory; 2024; doi: 10.1101/cshperspect.a041446.

6. Zhang L, Reifová R, Halenková Z, Gompert Z. How Important Are Structural Variants for Speciation? *Genes*. 2021; doi: 10.3390/genes12071084.

7. Janko K, Mikulíček P, Hobza R, Schlupp I. Sperm-dependent asexual species and their role in ecology and evolution. *Ecol Evol*. 2023; doi: 10.1002/ece3.10522.

8. Stenberg P, Saura A. Cytology of Asexual Animals. In: Schön I, Martens K, Dijk P, editors. *Lost Sex*. Dordrecht: Springer Netherlands;

9. Stenberg P, Saura A. Meiosis and Its Deviations in Polyploid Animals. *Cytogenet Genome Res*. 2013; doi: 10.1159/000351731.

10. Hörandl E, Bast J, Brandt A, Scheu S, Bleidorn C, Cordellier M, et al. Genome Evolution of Asexual Organisms and the Paradox of Sex in Eukaryotes. In: Pontarotti P, editor. *Evol Biol Transdiscipl Approach*. Cham: Springer International Publishing;

11. Jaron KS, Bast J, Nowell RW, Ranallo-Benavidez TR, Robinson-Rechavi M, Schwander T. Genomic Features of Parthenogenetic Animals. Orive M, editor. *J Hered*. 2021; doi: 10.1093/jhered/esaa031.

12. Kočí J, Röslein J, Pačes J, Kotusz J, Halačka K, Koščo J, et al. No evidence for accumulation of deleterious mutations and fitness degradation in clonal fish hybrids: Abandoning sex without regrets. *Mol Ecol*. 2020; doi: 10.1111/mec.15539.

13. Loewe L, Lamatsch DK. Quantifying the threat of extinction from Muller’s ratchet in the diploid Amazon molly (Poecilia formosa). *BMC Evol Biol*. 2008; doi: 10.1186/1471-2148-8-88.

14. Pellino M, Hojsgaard D, Schmutzer T, Scholz U, Hörandl E, Vogel H, et al. Asexual genome evolution in the apomictic *R anunculus auricomus* complex: examining the effects of hybridization and mutation accumulation. *Mol Ecol*. 2013; doi: 10.1111/mec.12533.

15. Marta A, Tichopád T, Bartoš O, Klíma J, Shah MA, Bohlen VŠ, et al. Genetic and karyotype divergence between parents affect clonality and sterility in hybrids. *eLife*. 2023; doi: 10.7554/eLife.88366.3.

16. Moritz C, Brown WM, Densmore LD, Wright JW, Vyas D, Donnellan S, et al. Genetic diversity and the dynamics of hybrid parthenogenesis in Cnemidophorus (Teiidae) and Heteronotia (Gekkonidae). *Evol Ecol Unisexual Vertebr*. p. 87–112.

17. Arai K, Fujimoto T. Genomic Constitution and Atypical Reproduction in Polyploid and Unisexual Lineages of the ***Misgurnus*** Loach, a Teleost Fish. *Cytogenet Genome Res*. 2013; doi: 10.1159/000353301.

18. Dedukh D, Majtánová Z, Marta A, Pšenička M, Kotusz J, Klíma J, et al. Parthenogenesis as a Solution to Hybrid Sterility: The Mechanistic Basis of Meiotic Distortions in Clonal and Sterile Hybrids. *Genetics*. 2020; doi: 10.1534/genetics.119.302988.

19. Lutes AA, Neaves WB, Baumann DP, Wiegraebe W, Baumann P. Sister chromosome pairing maintains heterozygosity in parthenogenetic lizards. *Nature*. 2010; doi: 10.1038/nature08818.

20. Stöck M, Dedukh D, Reifová R, Lamatsch DK, Starostová Z, Janko K. Sex chromosomes in meiotic, hemiclonal, clonal and polyploid hybrid vertebrates: along the 'extended speciation continuum'. *Philos Trans R Soc B Biol Sci*. 2021; doi: 10.1098/rstb.2020.0103.

21. Tichopád T, Franěk R, Doležálková-Kaštánková M, Dedukh D, Marta A, Halačka K, et al. Clonal gametogenesis is triggered by intrinsic stimuli in the hybrid's germ cells but is dependent on sex differentiation. *Biol Reprod*. 2022; doi: 10.1093/biolre/ioac074.

22. Parker DJ, Bast J, Jalvingh K, Dumas Z, Robinson-Rechavi M, Schwander T. Repeated Evolution of Asexuality Involves Convergent Gene Expression Changes. Parsch J, editor. *Mol Biol Evol*. 2019; doi: 10.1093/molbev/msy217.

23. Janko K, Bartoš O, Kočí J, Roslein J, Drdová EJ, Kotusz J, et al. Genome Fractionation and Loss of Heterozygosity in Hybrids and Polyploids: Mechanisms, Consequences for Selection, and Link to Gene Function. Satta Y, editor. *Mol Biol Evol*. 2021; doi: 10.1093/molbev/msab249.

24. Warren WC, García-Pérez R, Xu S, Lampert KP, Chalopin D, Stöck M, et al. Clonal polymorphism and high heterozygosity in the celibate genome of the Amazon molly. *Nat Ecol Evol*. 2018; doi: 10.1038/s41559-018-0473-y.

25. Bartoš O, Röslein J, Kotusz J, Paces J, Pekárik L, Petrtýl M, et al. The Legacy of Sexual Ancestors in Phenotypic Variability, Gene Expression, and Homoeolog Regulation of Asexual Hybrids and Polyploids. Wittkopp P, editor. *Mol Biol Evol*. 2019; doi: 10.1093/molbev/msz114.

26. Albertini E, Barcaccia G, Carman JG, Pupilli F. Did apomixis evolve from sex or was it the other way around? *J Exp Bot*. 2019; doi: 10.1093/jxb/erz109.

27. Janko K, Pačes J, Wilkinson-Herbots H, Costa RJ, Roslein J, Drozd P, et al. Hybrid asexuality as a primary postzygotic barrier between nascent species: On the interconnection between asexuality, hybridization and speciation. *Mol Ecol*. 2018; doi: 10.1111/mec.14377.

28. Murphy RW, Fu J, Macculloch RD, Darevsky IS, Kupriyanova LA. A fine line between sex and unisexuality: the phylogenetic constraints on parthenogenesis in lacertid lizards. *Zool J Linn Soc*. 2000; doi: 10.1111/j.1096-3642.2000.tb02200.x.

29. Janko K, Kotusz J, De Gelas K, Šlechtová V, Opoldusová Z, Drozd P, et al. Dynamic Formation of Asexual Diploid and Polyploid Lineages: Multilocus Analysis of Cobitis Reveals the Mechanisms Maintaining the Diversity of Clones. Steinke D, editor. *PLoS ONE*. 2012; doi: 10.1371/journal.pone.0045384.

30. Majtánová Z, Choleva L, Symonová R, Ráb P, Kotusz J, Pekárik L, et al. Asexual Reproduction Does Not Apparently Increase the Rate of Chromosomal Evolution: Karyotype Stability in Diploid and Triploid Clonal Hybrid Fish (Cobitis, Cypriniformes, Teleostei). Laudet V, editor. *PLOS ONE*. 2016; doi: 10.1371/journal.pone.0146872.

31. Janko K, Eisner J, Cigler P, Tichopád T. Unifying framework explaining how parental regulatory divergence can drive gene expression in hybrids and allopolyploids. *Nat Commun*. 2024; doi: 10.1038/s41467-024-52546-5.

32. Sambrook J, Russell DW. Purification of Nucleic Acids by Extraction with Phenol:Chloroform. *Cold Spring Harb Protoc*. 2006; doi: 10.1101/pdb.prot4455.

33. Rio DC, Ares M, Hannon GJ, Nilsen TW. Purification of RNA Using TRIzol (TRI Reagent). *Cold Spring Harb Protoc*. 2010; doi: 10.1101/pdb.prot5439.

34. Jackman SD, Vandervalk BP, Mohamadi H, Chu J, Yeo S, Hammond SA, et al. ABySS 2.0: resource-efficient assembly of large genomes using a Bloom filter. *Genome Res*. 2017; doi: 10.1101/gr.214346.116.

35. Li D, Liu C-M, Luo R, Sadakane K, Lam T-W. MEGAHIT: an ultra-fast single-node solution for large and complex metagenomics assembly via succinct de Bruijn graph. *Bioinforma Oxf Engl*. 2015; doi: 10.1093/bioinformatics/btv033.

36. Kolmogorov M, Yuan J, Lin Y, Pevzner PA. Assembly of long, error-prone reads using repeat graphs. *Nat Biotechnol*. 2019; doi: 10.1038/s41587-019-0072-8.

37. Loman NJ, Quick J, Simpson JT. A complete bacterial genome assembled de novo using only nanopore sequencing data. *Nat Methods*. 2015; doi: 10.1038/nmeth.3444.

38. Walker BJ, Abeel T, Shea T, Priest M, Abouelliel A, Sakthikumar S, et al. Pilon: An Integrated Tool for Comprehensive Microbial Variant Detection and Genome Assembly Improvement. Wang J, editor. *PLoS ONE*. 2014; doi: 10.1371/journal.pone.0112963.

39. Li H. Aligning sequence reads, clone sequences and assembly contigs with BWA-MEM. arXiv;

40. Danecek P, Bonfield JK, Liddle J, Marshall J, Ohan V, Pollard MO, et al. Twelve years of SAMtools and BCFtools. *GigaScience*. 2021; doi: 10.1093/gigascience/giab008.

41. Dudchenko O, Batra SS, Omer AD, Nyquist SK, Hoeger M, Durand NC, et al. De novo assembly of the *Aedes aegypti* genome using Hi-C yields chromosome-length scaffolds. *Science*. 2017; doi: 10.1126/science.aal3327.

42. Bushnell B. BBMap: A Fast, Accurate, Splice-Aware Aligner. Walnut Creek, CA, United States: Lawrence Berkeley National Lab. (LBNL), Berkeley, CA (United States);

43. Wolff J, Rabbani L, Gilsbach R, Richard G, Manke T, Backofen R, et al. Galaxy HiCExplorer 3: a web server for reproducible Hi-C, capture Hi-C and single-cell Hi-C data analysis, quality control and visualization. *Nucleic Acids Res*. 2020; doi: 10.1093/nar/gkaa220.

44. Kruse K, Hug CB, Vaquerizas JM. FAN-C: a feature-rich framework for the analysis and visualisation of chromosome conformation capture data. *Genome Biol*. 2020; doi: 10.1186/s13059-020-02215-9.

45. Álvarez-González L, Burden F, Doddamani D, Malinverni R, Leach E, Marín-García C, et al. 3D chromatin remodelling in the germ line modulates genome evolutionary plasticity. *Nat Commun*. 2022; doi: 10.1038/s41467-022-30296-6.

46. Flynn JM, Hubley R, Goubert C, Rosen J, Clark AG, Feschotte C, et al. RepeatModeler2 for automated genomic discovery of transposable element families. *Proc Natl Acad Sci*. 2020; doi: 10.1073/pnas.1921046117.

47. Bao Z, Eddy SR. Automated De Novo Identification of Repeat Sequence Families in Sequenced Genomes. *Genome Res*. 2002; doi: 10.1101/gr.88502.

48. Price AL, Jones NC, Pevzner PA. De novo identification of repeat families in large genomes. *Bioinformatics*. 2005; doi: 10.1093/bioinformatics/bti1018.

49. Smit AFA, Hubley R, Green P. *RepeatMasker Open-4.0*. 2013-2015. https://www.repeatmasker.org.

50. Stanke M, Morgenstern B. AUGUSTUS: a web server for gene prediction in eukaryotes that allows user-defined constraints. *Nucleic Acids Res*. 2005; doi: 10.1093/nar/gki458.

51. Korf I. Gene finding in novel genomes. *BMC Bioinformatics*. 2004; doi: 10.1186/1471-2105-5-59.

52. Dobin A, Davis CA, Schlesinger F, Drenkow J, Zaleski C, Jha S, et al. STAR: ultrafast universal RNA-seq aligner. *Bioinformatics*. 2013; doi: 10.1093/bioinformatics/bts635.

53. Keller O, Kollmar M, Stanke M, Waack S. A novel hybrid gene prediction method employing protein multiple sequence alignments. *Bioinformatics*. 2011; doi: 10.1093/bioinformatics/btr010.

54. Stanke M, Keller O, Gunduz I, Hayes A, Waack S, Morgenstern B. AUGUSTUS: ab initio prediction of alternative transcripts. *Nucleic Acids Res*. 2006; doi: 10.1093/nar/gkl200.

55. Stanke M, Diekhans M, Baertsch R, Haussler D. Using native and syntenically mapped cDNA alignments to improve *de novo* gene finding. *Bioinformatics*. 2008; doi: 10.1093/bioinformatics/btn013.

56. Campbell MS, Holt C, Moore B, Yandell M. Genome Annotation and Curation Using MAKER and MAKER-P. *Curr Protoc Bioinforma*. 2014; doi: 10.1002/0471250953.bi0411s48.

57. The UniProt Consortium, Bateman A, Martin M-J, Orchard S, Magrane M, Adesina A, et al. UniProt: the Universal Protein Knowledgebase in 2025. *Nucleic Acids Res*. 2025; doi: 10.1093/nar/gkae1010.

58. Altschul SF, Gish W, Miller W, Myers EW, Lipman DJ. Basic local alignment search tool. *J Mol Biol*. 1990; doi: 10.1016/S0022-2836(05)80360-2.

59. Chan PP, Lin BY, Mak AJ, Lowe TM. tRNAscan-SE 2.0: improved detection and functional classification of transfer RNA genes. *Nucleic Acids Res*. 2021; doi: 10.1093/nar/gkab688.

60. Shumate A, Wong B, Pertea G, Pertea M. Improved transcriptome assembly using a hybrid of long and short reads with StringTie. Li J, editor. *PLOS Comput Biol*. 2022; doi: 10.1371/journal.pcbi.1009730.

61. Haas B. TransDecoder (2024). *TransDecoder* (Version 5.7.1.) https://github.com/TransDecoder/TransDecoder.

62. Dainat J. NBISweden/AGAT: AGAT (2024). AGAT (Version 1. 4.1). Zenodo. https://github.com/NBISweden/AGAT/compare/v1.4.0...v1.4.1.

63. Kuhl H. HANNO: efficient High-throughput ANNOtation of protein coding genes in eukaryote genomes. Zenodo. https://zenodo.org/doi/10.5281/zenodo.11532370.

64. Kim D, Paggi JM, Park C, Bennett C, Salzberg SL. Graph-based genome alignment and genotyping with HISAT2 and HISAT-genotype. *Nat Biotechnol*. 2019; doi: 10.1038/s41587-019-0201-4.

65. Storer J, Hubley R, Rosen J, Wheeler TJ, Smit AF. The Dfam community resource of transposable element families, sequence models, and genome annotations. *Mob DNA*. 2021; doi: 10.1186/s13100-020-00230-y.

66. Li H. Minimap2: pairwise alignment for nucleotide sequences. Birol I, editor. *Bioinformatics*. 2018; doi: 10.1093/bioinformatics/bty191.

67. Cabanettes F, Klopp C. D-GENIES: dot plot large genomes in an interactive, efficient and simple way. *PeerJ*. 2018; doi: 10.7717/peerj.4958.

68. Goel M, Sun H, Jiao W-B, Schneeberger K. SyRI: finding genomic rearrangements and local sequence differences from whole-genome assemblies. *Genome Biol*. 2019; doi: 10.1186/s13059-019-1911-0.

69. He W, Yang J, Jing Y, Xu L, Yu K, Fang X. NGenomeSyn: an easy-to-use and flexible tool for publication-ready visualization of syntenic relationships across multiple genomes. Marschall T, editor. *Bioinformatics*. 2023; doi: 10.1093/bioinformatics/btad121.

70. Tang H, Krishnakumar V, Zeng X, Xu Z, Taranto A, Lomas JS, et al. JCVI: A versatile toolkit for comparative genomics analysis. *iMeta*. 2024; doi: 10.1002/imt2.211.

71. Quinlan AR. BEDTools: The Swiss-Army Tool for Genome Feature Analysis. *Curr Protoc Bioinforma*. 2014; doi: 10.1002/0471250953.bi1112s47.

72. McKenna A, Hanna M, Banks E, Sivachenko A, Cibulskis K, Kernytsky A, et al. The Genome Analysis Toolkit: A MapReduce framework for analyzing next-generation DNA sequencing data. *Genome Res*. 2010; doi: 10.1101/gr.107524.110.

73. Danecek P, Auton A, Abecasis G, Albers CA, Banks E, DePristo MA, et al. The variant call format and VCFtools. *Bioinformatics*. 2011; doi: 10.1093/bioinformatics/btr330.

74. Quinlan AR, Hall IM. BEDTools: a flexible suite of utilities for comparing genomic features. *Bioinformatics*. 2010; doi: 10.1093/bioinformatics/btq033.

75. Marta A, Dedukh D, Bartoš O, Majtánová Z, Janko K. Cytogenetic Characterization of Seven Novel satDNA Markers in Two Species of Spined Loaches (Cobitis) and Their Clonal Hybrids. *Genes*. 2020; doi: 10.3390/genes11060617.

76. Zhang T. (2024). Chorus2 (Version 2.0.3). https://github.com/zhangtaolab/Chorus2.

77. Janko K, Flajšhans M, Choleva L, Bohlen J, ŠLechtová V, Rábová M, et al. Diversity of European spined loaches (genus *Cobitis* L.): an update of the geographic distribution of the *Cobitis taenia* hybrid complex with a description of new molecular tools for species and hybrid determination. *J Fish Biol*. 2007; doi: 10.1111/j.1095-8649.2007.01663.x.

78. Pérez-Rico YA, Barillot E, Shkumatava A. Demarcation of Topologically Associating Domains Is Uncoupled from Enriched CTCF Binding in Developing Zebrafish. *iScience*. 2020; doi: 10.1016/j.isci.2020.101046.

79. Kim J, Lee C, Ko BJ, Yoo DA, Won S, Phillippy AM, et al. False gene and chromosome losses in genome assemblies caused by GC content variation and repeats. *Genome Biol*. 2022; doi: 10.1186/s13059-022-02765-0.

80. Vara C, Paytuví-Gallart A, Cuartero Y, Álvarez-González L, Marín-Gual L, Garcia F, et al. The impact of chromosomal fusions on 3D genome folding and recombination in the germ line. *Nat Commun*. 2021; doi: 10.1038/s41467-021-23270-1.

81. Zhang L, Zhang W, Cheng Y, Fang Y, Guan X, Gong A, et al. Chromosome-level genome assembly and annotation of the gynogenetic large-scale loach (Paramisgurnus dabryanus). *Sci Data*. 2025; doi: 10.1038/s41597-025-04498-8.

82. Wang X, Wang D, Wang H, Dudgeon D, Reid K, Merilä J. Chromosome-level haplotype-resolved genome of the tropical loach (Oreonectes platycephalus). *Sci Data*. 2025; doi: 10.1038/s41597-024-04301-0.

83. Šlechtová V, Musilova Z, Tan HH, Kottelat M, Bohlen J. One northward, one southward: Contrasting biogeographical history in two benthic freshwater fish genera across Southeast Asia (Teleostei: Cobitoidea: Nemacheilus, Pangio). *Mol Phylogenet Evol*. 2021; doi: 10.1016/j.ympev.2021.107139.

84. Perdices A, Bohlen J, Šlechtová V, Doadrio I. Molecular Evidence for Multiple Origins of the European Spined Loaches (Teleostei, Cobitidae). Peng Z, editor. *PLOS ONE*. 2016; doi: 10.1371/journal.pone.0144628.

85. Stevison LS, Hoehn KB, Noor MAF. Effects of Inversions on Within- and Between-Species Recombination and Divergence. *Genome Biol Evol*. 2011; doi: 10.1093/gbe/evr081.

86. Hooper DM, Price TD. Chromosomal inversion differences correlate with range overlap in passerine birds. *Nat Ecol Evol*. 2017; doi: 10.1038/s41559-017-0284-6.

87. Shao F, Han M, Peng Z. Evolution and diversity of transposable elements in fish genomes. *Sci Rep*. 2019; doi: 10.1038/s41598-019-51888-1.

88. He C, Zhang X, Wen Z, Shi Q, Song Z. A chromosome-scale reference genome assembly for Triplophysa lixianensis. *Sci Data*. Springer Science and Business Media LLC; 2024; doi: 10.1038/s41597-024-04268-y.

89. Deng Y, Meng M, Fang J, Jiang H, Sun N, Lv W, et al. Genome of the butterfly hillstream loach provides insights into adaptations to torrential mountain stream life. *Mol Ecol Resour*. Wiley; 2021; doi: 10.1111/1755-0998.13400.

90. Sotero-Caio CG, Platt RN, Suh A, Ray DA. Evolution and Diversity of Transposable Elements in Vertebrate Genomes. *Genome Biol Evol*. 2017; doi: 10.1093/gbe/evw264.

91. Jurka J, Bao W, Kojima KK. Families of transposable elements, population structure and the origin of species. *Biol Direct*. 2011; doi: 10.1186/1745-6150-6-44.

92. Oliver KR, Greene WK. Mobile DNA and the TE-Thrust hypothesis: supporting evidence from the primates. *Mob DNA*. 2011; doi: 10.1186/1759-8753-2-8.

93. Platt RN, Vandewege MW, Kern C, Schmidt CJ, Hoffmann FG, Ray DA. Large Numbers of Novel miRNAs Originate from DNA Transposons and Are Coincident with a Large Species Radiation in Bats. *Mol Biol Evol*. 2014; doi: 10.1093/molbev/msu112.

94. Heule C, Salzburger W, Böhne A. Genetics of Sexual Development: An Evolutionary Playground for Fish. *Genetics*. 2014; doi: 10.1534/genetics.114.161158.

95. Mank JE, Avise JC. Evolutionary Diversity and Turn-Over of Sex Determination in Teleost Fishes. *Sex Dev*. 2009; doi: 10.1159/000223071.

96. Jeffries D, Benvenuto C, Böhne A, Fraisse C, Garcia S, Jay P, et al. The Tree of Sex consortium: A global initiative for studying the evolution of reproduction in eukaryotes. *J Evol Biol*. 2025; doi: 10.1093/jeb/voaf053.

97. Vasil'eva ED, Vasil'ev VP. Sibling species in genus Cobitis (Cobitidae). Cobitis rossomeridionalis sp. nova. *J Ichthyol*. 1998;

98. Sharma OP, Tripathi NK. Female heterogamety in two teleostean fishes. *Cytologia (Tokyo)*. 1988; doi: 10.1508/cytologia.53.81.

99. Saitoh K. Multiple sex-chromosome system in a loach fish. *Cytogenet Genome Res*. 1989; doi: 10.1159/000132840.

100. Bhattacharyya T, Gregorova S, Mihola O, Anger M, Sebestova J, Denny P, et al. Mechanistic basis of infertility of mouse intersubspecific hybrids. *Proc Natl Acad Sci*. 2013; doi: 10.1073/pnas.1219126110.

101. Forejt J, Jansa P. Meiotic Recognition of Evolutionarily Diverged Homologs: Chromosomal Hybrid Sterility Revisited. Malik H, editor. *Mol Biol Evol*. 2023; doi: 10.1093/molbev/msad083.

102. Dedukh D, Marta A, Janko K. Challenges and Costs of Asexuality: Variation in Premeiotic Genome Duplication in Gynogenetic Hybrids from Cobitis taenia Complex. *Int J Mol Sci*. 2021; doi: 10.3390/ijms222212117.

103. Tucker AE, Ackerman MS, Eads BD, Xu S, Lynch M. Population-genomic insights into the evolutionary origin and fate of obligately asexual *Daphnia pulex*. *Proc Natl Acad Sci*. 2013; doi: 10.1073/pnas.1313388110.

Figure captions

Figure 1. Map of European rivers indicating the distribution ranges of the three *Cobitis* species included in this study. *C. taenia* is in blue, *C. tanaitica* is in green and *C. elongatoides* is in yellow. Pie charts indicate the sample size and sex ratio of samples taken from each locality (males are indicated by the darker colour and females by the lighter one). The insert indicates a *C. taenia* female individual.

Figure 2. *Cobitis* genomes higher-order chromatin organization. (A) Genome-wide Hi-C contact maps. Contact maps represent 500 Kbp resolution Hi-C matrices obtained using the final assembly as a reference. For the three species clear interacting blocks corresponding to the expected number of chromosomes can be observed. (B) Boxplot depicting the 1st eigenvector distribution of the three species. Eigenvector values are used as a proxy to determine open (A compartments) and close (B compartments) chromatin regions. The similarities in the distribution between the three species indicate similar 3D organization (two-sided t test, ns $p > 0.05$). (C) Boxplot showing insulator score distribution on the three species. Insulator capacity is used to determine TADs strength. Like eigenvector distribution, similarities on the insulator score reflect the same patterns of chromatin folding in the three species (two-sided t test, ns $p > 0.05$). (D) Region-specific 500 Kbp heatmaps, 1st eigenvector and insulator score tracks in the three species. Similar tendencies can be clearly observed.

Figure 3. Syntenic and rearranged regions in Cobitis species. A) Gene synteny plot of three Cobitis species and Paramisgurnus dabryanus (from left to right Cobitis tanaitica, C. taenia, C. elongatoides and P. dabryanus). B) Synteny plots of homologous sequences and intrachromosomal rearrangements in four selected chromosomes (chromosome 1, chromosome 4, chromosome 5, chromosome 20) from the three species (from top to bottom C. tanaitica, C. taenia and C. elongatoides). Darker colour shades highlight the SyRI detected events while lighter shades show the results of parsimonious merging. Only blocks longer than 5 Kbp are shown.

Figure 4. Repeatome in *Cobitis*. A) A comparison of relative distribution of individual TE families for each genome (coloured by species). Black bars show the absolute lengths of each family in *C. taenia*. B) The repeat landscape plot illustrates the transposable element (TE) accumulation history for the three *Cobitis* genomes (*C. taenia*, *C. tanaitica*, and *C. elongatoides*), based on Kimura distance-based copy divergence analyses. The sequence divergence (CpG adjusted Kimura substitution level) is shown on the x-axis while the percentage of the genome represented by each TE type is on the y-axis. Transposon type is indicated by the key on the right.

Figure 5. Signals of sex-linked differentiation across the three *Cobitis* genomes. Differences in coverage between male and female individuals are shown on the top row (A-C), with values representing the log2 transformed coverage in females divided by the coverage in males. The concentrations of sex-specific SNPs are shown on the bottom row (D-F) with values normalised by the number of SNPs identified in that region. Each data point represents the total across a window of 200 Kbp of the genome, with consecutive windows starting 50 Kbp apart. *C. taenia* (A, D) and *C. elongatoides* (C, F) are created using pooled DNA from males and females while *C. tanaitica* (B, E) is created from 3 male and 3 female individuals. Arrows point to the most promising (if any) differentiated sex regions.

Figure 6. Chromosome painting of selected chromosomes. Ch01A (red) and Ch01B (green) (A-D) and Ch05 (red) and Ch20 (green) (E-H) are shown on mitotic metaphases of *C. taenia* (A, E), *C. tanaitica* (B, F), *C. elongatoides* (C, G), and diploid ET hybrid (D, H) males. Ch01A and Ch01B are located on different arms of the largest metacentric chromosome in *C. taenia* (A). In *C. tanaitica,* signals appeared on two pairs of acrocentric chromosomes (B) and in *C. elongatoides*, they were located on two pairs of submetacentric chromosomes (C). Small submetacentric chromosome stained by Ch01A represents the sex chromosome of *C. elongatoides* (C). In diploid ET hybrid, both signals were detected on one metacentric chromosome of *C. taenia* (pointed by arrow) and two submetacentric chromosomes of *C. elongatoides* (D). Chromosome painting of Ch05 showed signals on the long arm of a large submetacentric chromosome across all species (E-G) and in diploid hybrid (H). Chromosome

painting of Ch20 was detected in the q-arm of a subtelocentric chromosome in *C. taenia* (E) and *C. elongatoides* (G) and corresponding chromosomes in the diploid hybrid (H) but locates in a q-arm of a subtelocentric chromosome in *C. tanaitica* (F). Chromosomes are stained by DAPI (blue). Scale bar = 10 µm.

Figure 7. Chromosome pairing in hybrid males. (A-C) Chromosome painting of Ch05 (green) and Ch20 (red) (A) as well as Ch01A (green) and Ch01B (red) (B, C) on meiotic metaphases of diploid hybrid males. Probes for Ch01A and Ch01B hybridised to the *C. taenia* chromosome and two small chromosomes of *C. elongatoides* (B, C). Chr01A of *C. elongatoides* usually existed as univalent (B, C). In some spermatocytes, *C. elongatoides* chromosome hybridising with probe for Chr01B showed pairing with homologous arm of Chr01B of *C. taenia* (B), while in other spermatocytes, there was no pairing between part of the Chr01B of *C. taenia* and chromosome Chr01B of *C. elongatoides* (C). Chromosomes are stained by DAPI (blue). Scale bar = 10 µm. (D) Mosaic plot showing pairing success of four chromosomes (Ch01A, Ch01B, Ch05, Ch20) in two diploid hybrid males. Each chromosome is represented by two adjacent bars (one per individual), with bar height normalised to 100% (brown bars represent the individual one and blue ones the individual two). The lower shaded section indicates the proportion of cells where the chromosome formed a bivalent, while the upper section represents univalents. Numbers within each section show absolute counts. Bar width reflects the total number of spermatocytes analysed per chromosome and individual. Ch01B exhibited the highest pairing frequency, whereas Ch05, Ch20, and especially Ch01A were mostly unpaired.

Table captions

Table 1. Primers used for PCR amplification and the number of tested individuals of *C. elongatoides* and *C. taenia*. A minus sign (-) indicates no detectable signal in electrophoresis after PCR amplification, while a plus sign (+) indicates the presence of a strong band.

Table 2. Final genome assembly statistics for the three *Cobitis* species.

Table 1. Primers used for PCR amplification and the number of tested individuals of *C. elongatoides* and *C. ta*
detectable signal in electrophoresis after PCR amplification, while a plus sign (+) indicates the presence of a s

Forward primer	Reverse primer	Species	Chromosome
SexMark_EE_M_p2F ACCCTCGTTTGGCTCAGATG	SexMark_EE_M_p7R AGAGGTACGCTAAAATGGGACA	*C. elongatoides*	Ch01A (Y)
SexMark_EE_F_p4F ACACCTGGCAGGCCAAATAA	SexMark_EE_F_p4R AAGGCCACAGTCCAAAACCA	*C. elongatoides*	Ch01A (X)
SexMark_TT_M_p2F TGACCCTGACTAATTCGGAAAAC	SexMark_TT_M_p5R ACGAGAATATAACACGACTTTCCA	*C. taenia*	Ch05 (Y)

aenia . A minus sign (-) indicates no
strong band.

C. elongatoides		*C. taenia*	
**Females**	**Males**	**Females**	**Males**
11-	18+	16-	16-
11+	17+	14-	14-
11-	15-	13-	14+

Click here to access/download;Table;Table 2.xlsx

Table 2: Final genome assembly statistics for the three *Cobitis* species.

Species	*C. taenia*	*C. tanaitica*	*C. elongatoides*
Number of Contigs	7.2k	25.8k	28.9k
Cumulative Length (Gbp)	1.63	1.71	1.82
N50 (Mbp)	53.2	51.3	43
L50	13.00	14.00	17.00
Number of Chromosomes	24.00	25.00	25.00
Proportion of genome assembled into chromosomes	85.80%	69.80%	68.40%

Figure 1

Click here to access/download;Figure;Figure 1.tiff

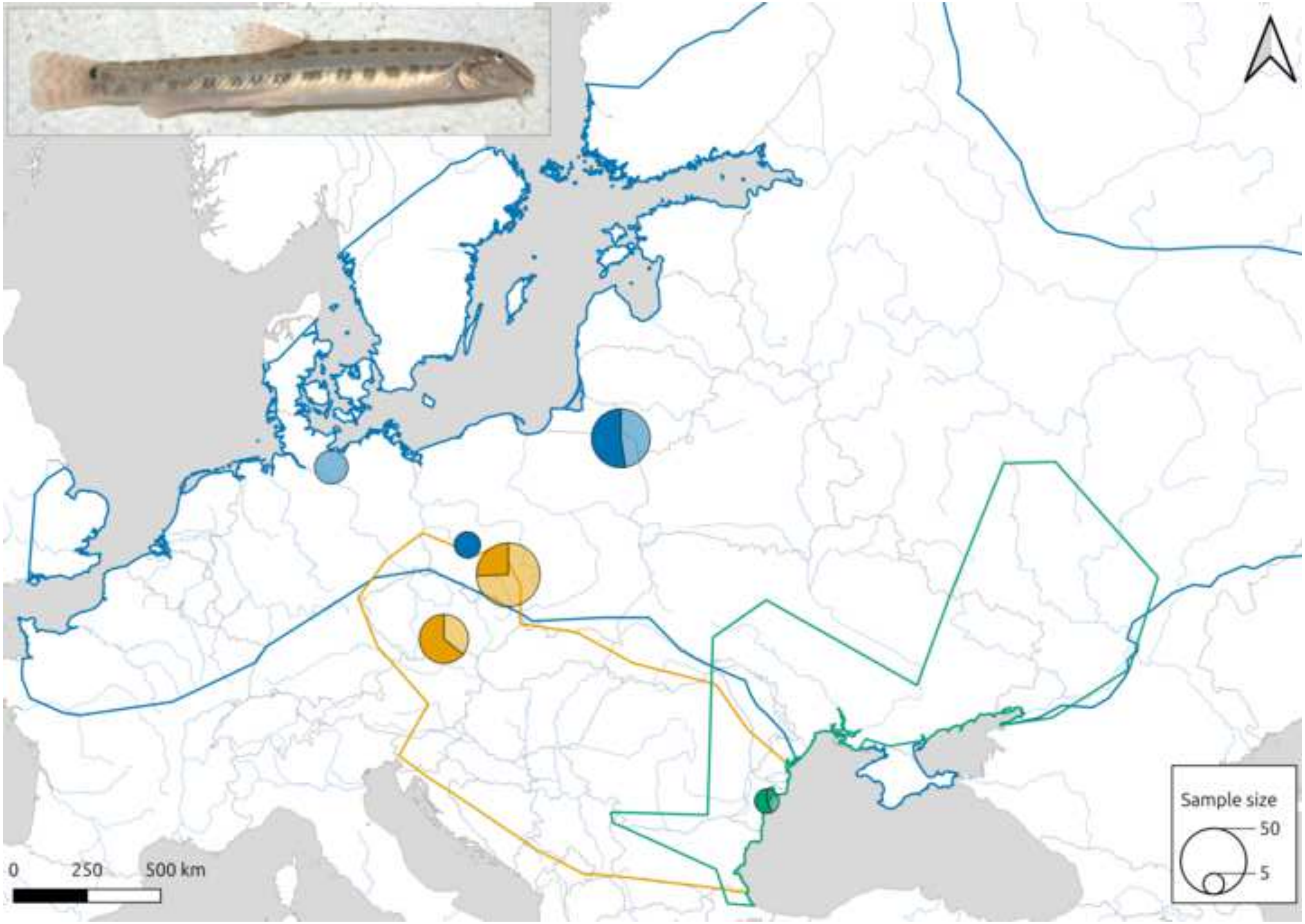

Figure 2

Click here to access/download;Figure;Figure 2.tiff

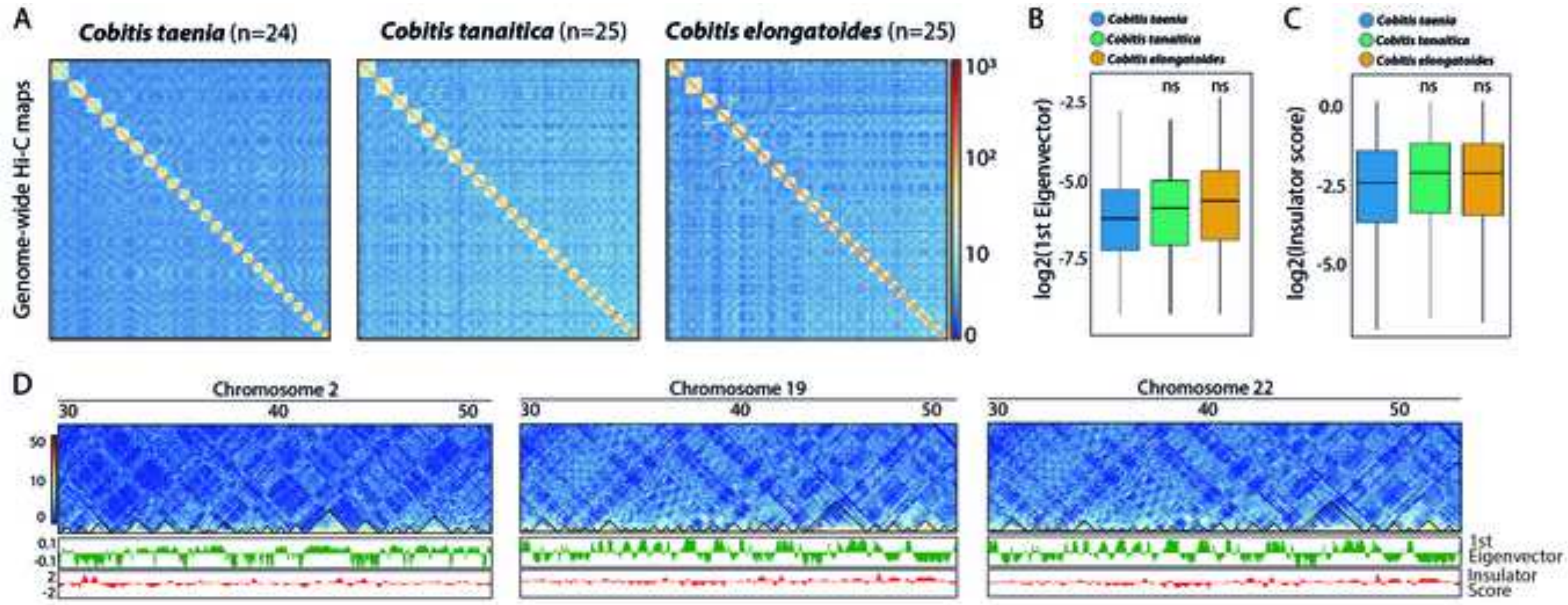

Figure 3

Click here to access/download;Figure;Figure 3.tiff

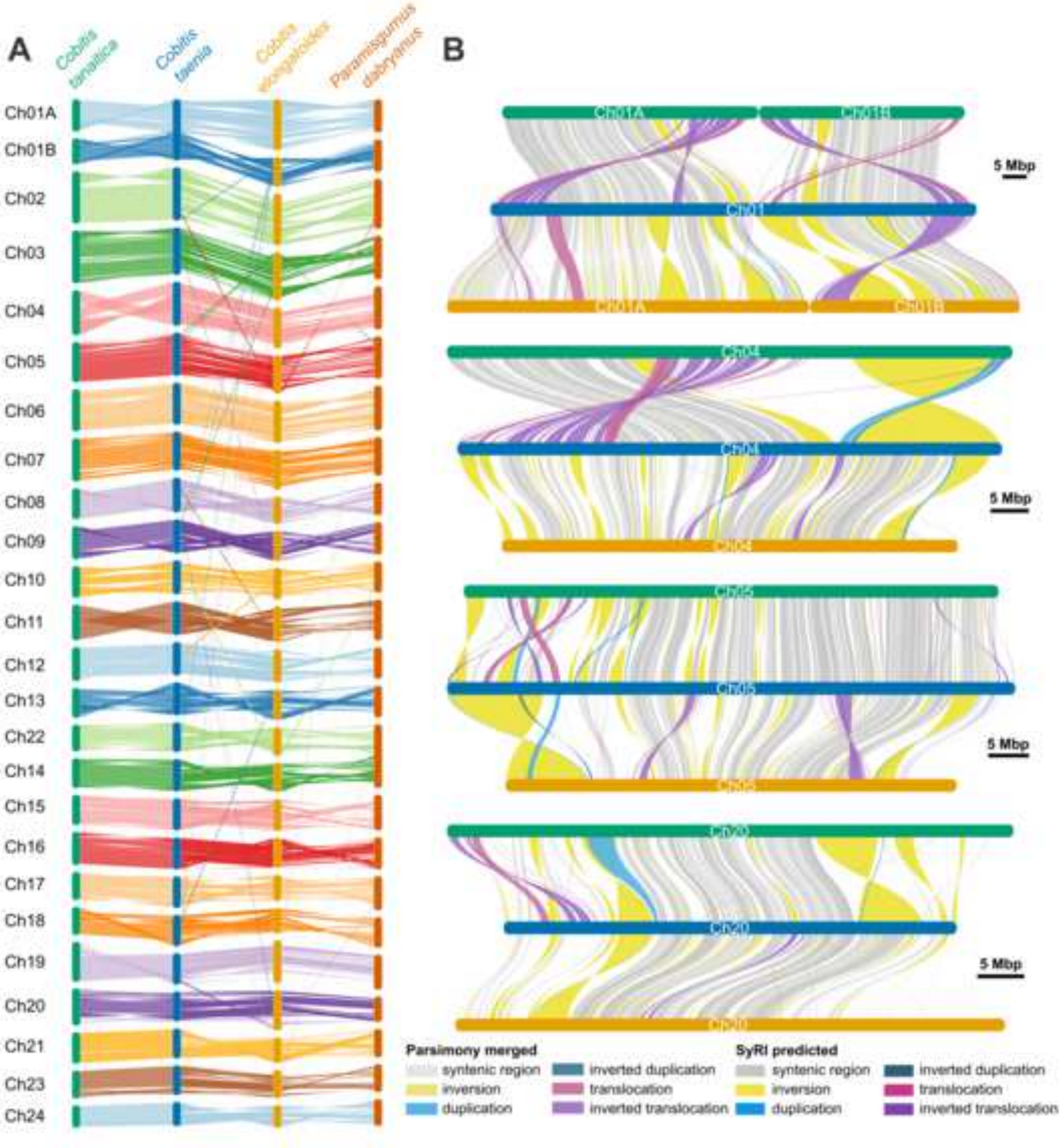

Figure 4

Click here to access/download;Figure;Figure 4.tif

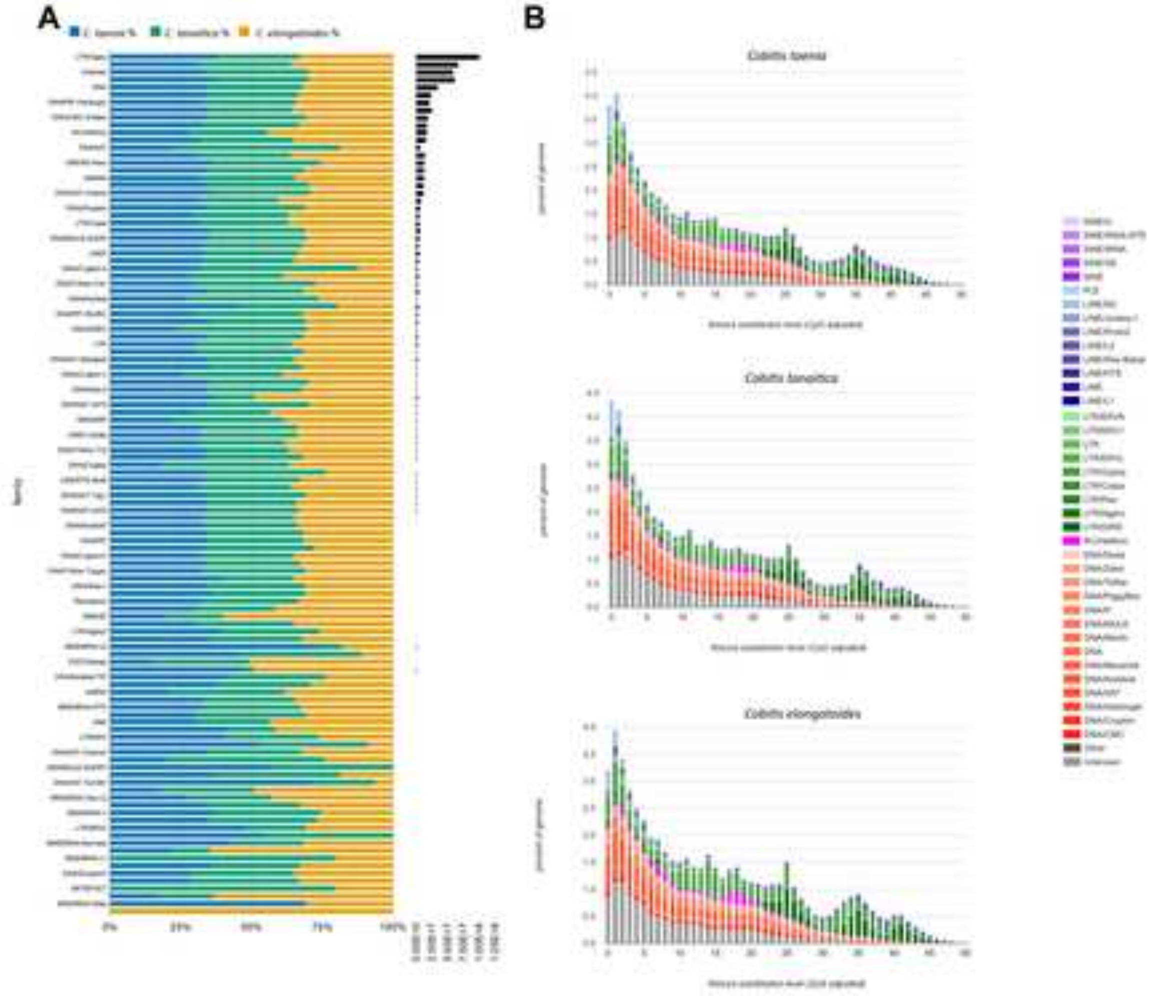

Figure 5

Click here to access/download;Figure;Figure 5.tiff

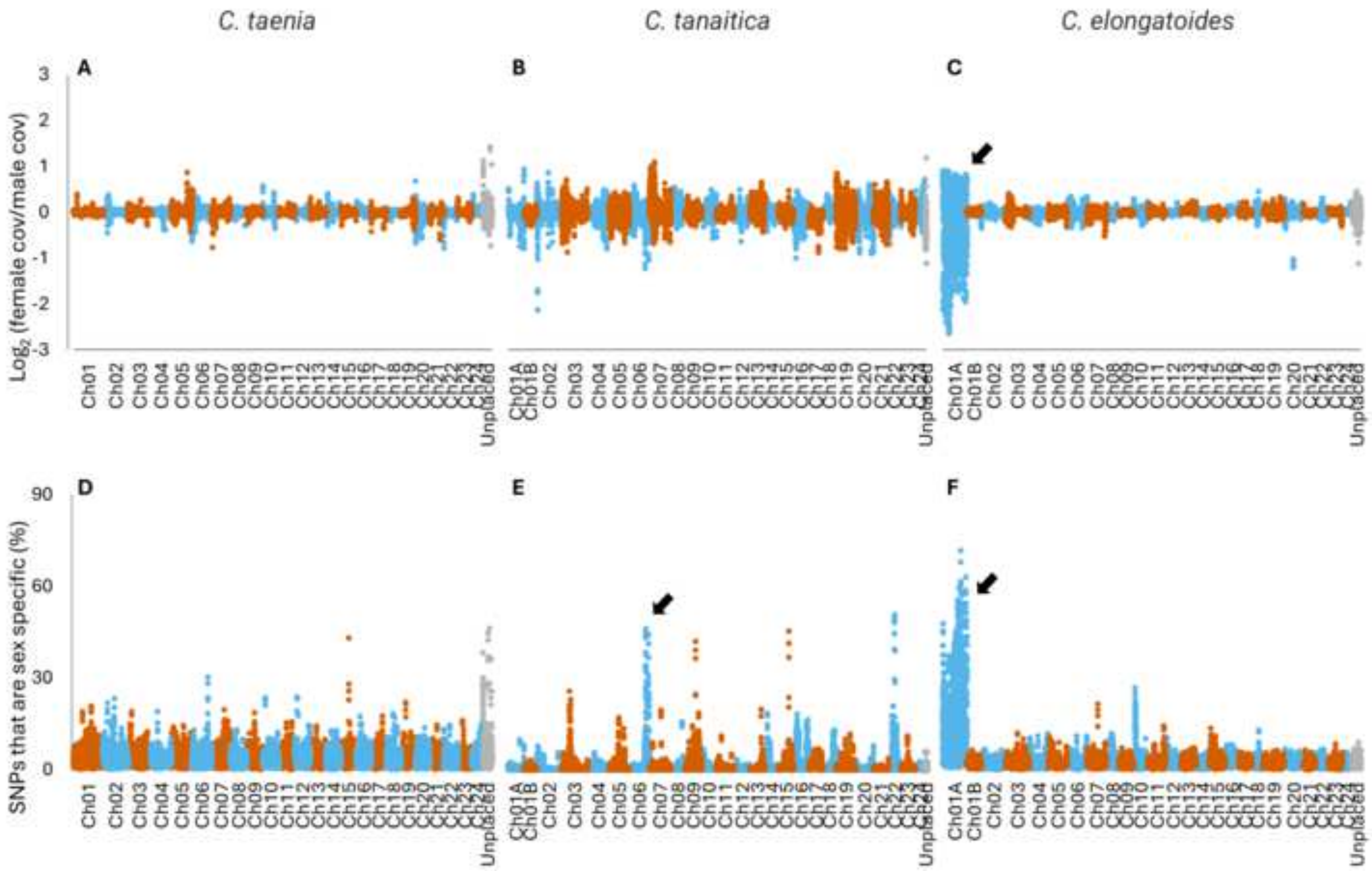

Figure 6

Click here to access/download;Figure;Figure 6.tif

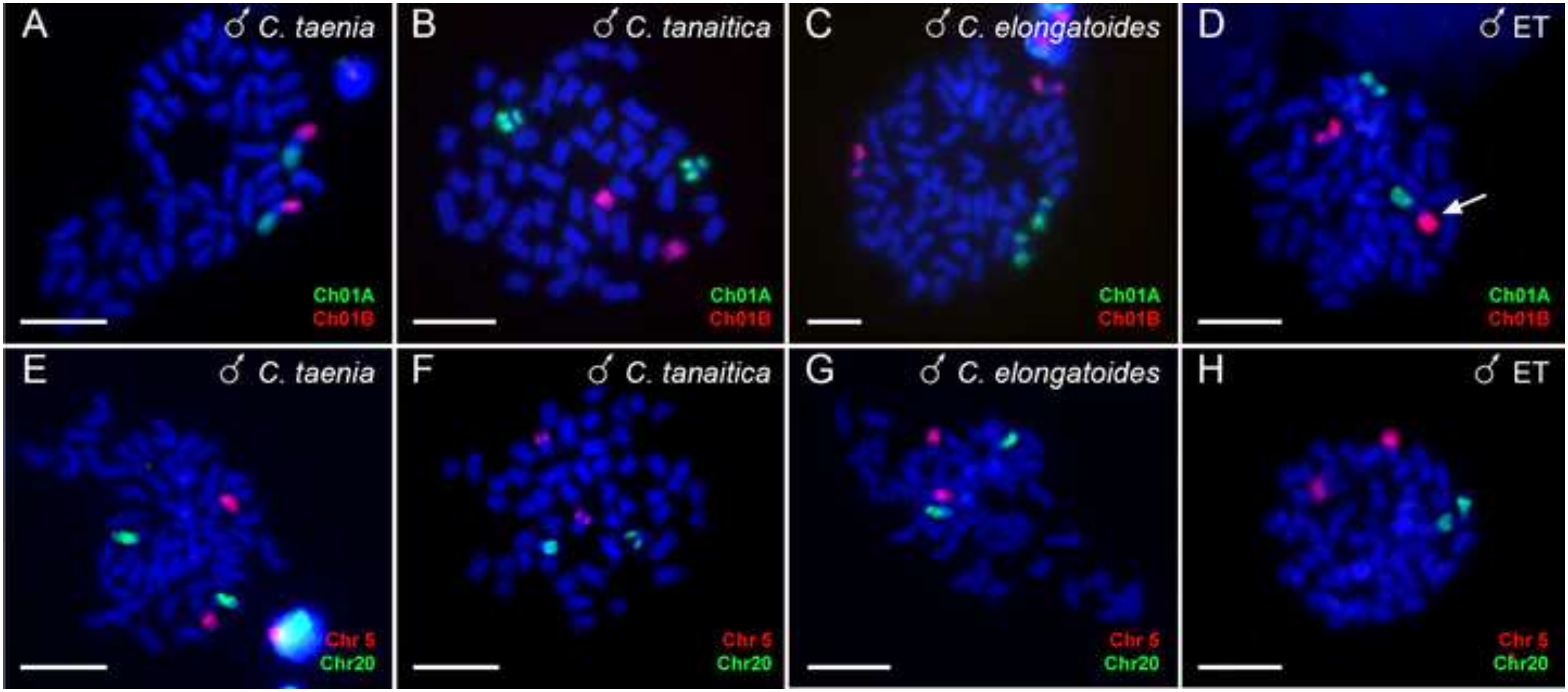

Figure 7

Click here to access/download;Figure;Figure 7.tif

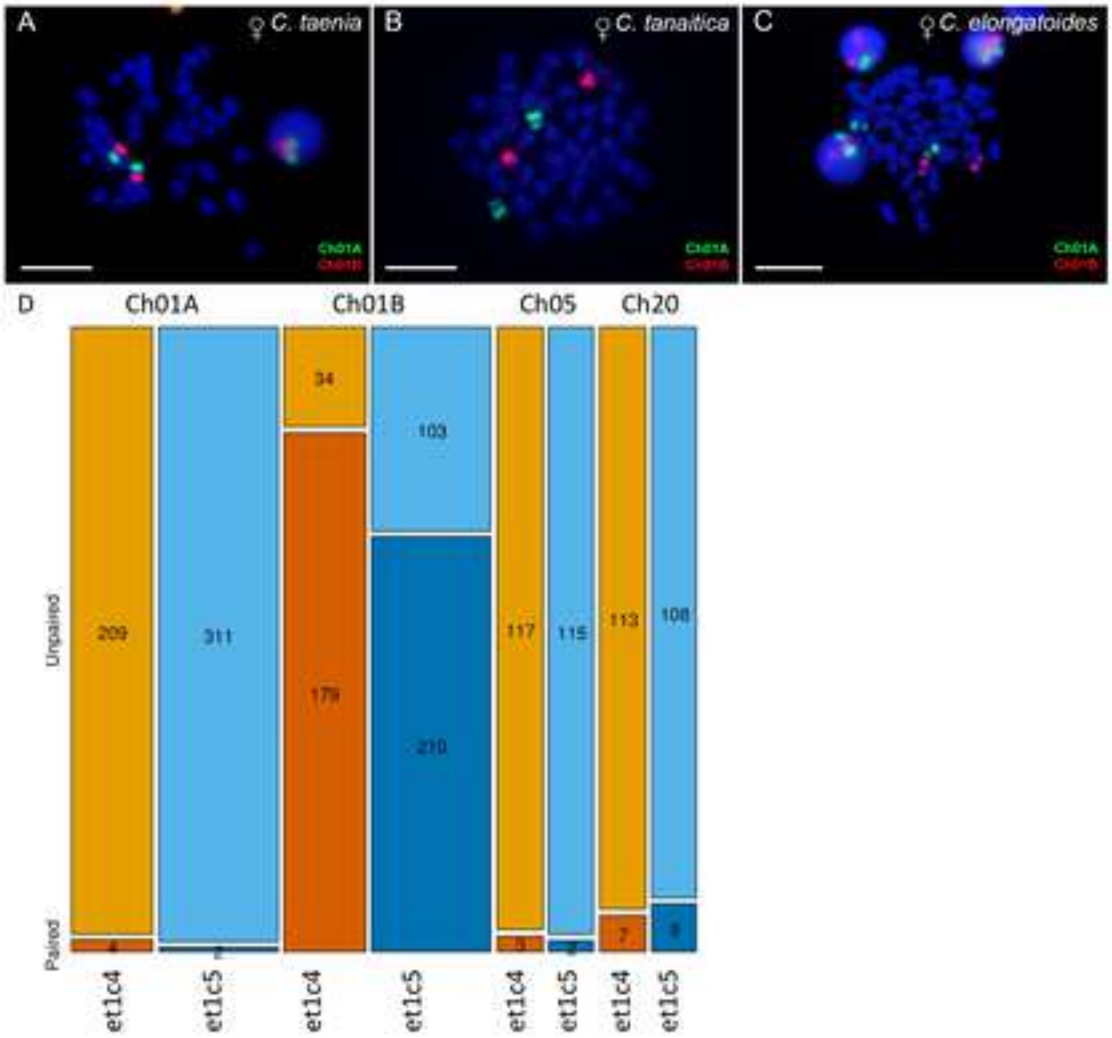

Supplementary Figures

Click here to access/download
Supplementary Material
Supplementary Figures.pdf

Click here to access/download
Supplementary Material
Supplementary Tables.xlsx
