# Supplementary material for: Sex chromosome turnover and structural genome divergence shape meiotic outcomes in hybridizing Cobitis
Source: Gigascience. 2026 Mar 24;15:giag031. doi: 10.1093/gigascience/giag031 (PMC13175044; doi:10.1093/gigascience/giag031)
Supplement: giag031_GIGA-D-25-00241_Revision_1 [file giag031_giga-d-25-00241_revision_1.pdf]

GigaScience

Sex Chromosome Turnover and Structural Genome Divergence Shapes Meiotic Outcomes in Hybridising Cobitis

--Manuscript Draft--

**Manuscript Number:**	GIGA-D-25-00241R1
**Full Title:**	Sex Chromosome Turnover and Structural Genome Divergence Shapes Meiotic Outcomes in Hybridising Cobitis
**Article Type:**	Research

Funding Information:

Funder	Recipient
Grantová Agentura České Republiky (24-12217S)	Dr. Karel Janko
HORIZON EUROPE Marie Sklodowska-Curie Actions (101081195)	Dr. Vladimir Trifonov
Spanish Ministry of Science and Innovation (PID2020-112557GB-I00)	Prof. Aurora Ruiz-Herrera
Agència de Gestió d'Ajuts Universitaris i de Recerca (2021SGR00122)	Prof. Aurora Ruiz-Herrera
Ministerio de Economía y Competitividad (PRE-2018-083257)	Dr. Lucia Álvarez-González
Ministerio de Economía y Competitividad (PRE-C-2021-0083)	MSc Gala Pujol
Grantová Agentura, Univerzita Karlova (314222)	MSc Zuzana Halenková
Charles University Research Centre program (UNCE/24/SCI/006)	Dr. Stephen A. Schlebusch

Abstract:

Background: Hybridisation between divergent species can result in meiotic aberrations and the emergence of asexual reproduction. Yet, it remains poorly understood to what extent such outcomes arise from genome-wide incompatibilities versus more specific conflicts among individual chromosomes inherited from parental species, including their ability to pair during meiosis in hybrids. It is also unclear how interspecific hybrids cope with differences in sex determination systems, particularly in the context of increased ploidy. Addressing these questions requires high-quality, chromosome-level reference genomes of the parental species involved in hybrid formation.

Findings: Here, we present the first chromosome-level genome assemblies for three hybridising Cobitis species (C. elongatoides, C. taenia, and C. tanaitica), providing a comprehensive framework for investigating the genomic and cytogenetic basis of hybrid sterility and the transition to asexuality. By integrating genome scaffolding, male/female pooled sequencing (Pool-Seq), and molecular cytogenetics, we uncover extensive structural variation among homologous chromosomes of the three species, despite overall karyotype conservation. Population-level analyses revealed that each species possesses distinct, non-homologous sex chromosomes, highlighting rapid sex chromosome turnover in this recently diverged lineage. Finally, the design of chromosome-specific painting probes, which we applied to meiotic metaphase I spreads of diploid hybrids. This approach revealed striking differences in the pairing success of orthologous chromosomes.

Conclusions: Our results demonstrate that individual orthologous chromosomes differ markedly in their ability to form bivalents during meiosis in hybrids, indicating that hybrid meiotic behaviour is shaped by chromosome-specific incompatibilities rather than uniform genome-wide failure. We also found that even closely related parental species possess distinct, non-homologous sex chromosomes, highlighting rapid turnover of sex determination systems in hybridising lineages. Together, these findings provide a high-resolution genomic and cytogenetic framework to explore how the architecture of inherited parental genomes influences sex-specific reproductive outcomes in hybrids—ranging from male sterility to the establishment of fertile, clonally reproducing female lineages—and how such asymmetries may contribute to the emergence of asexuality in vertebrates.

Powered by Editorial Manager® and ProduXion Manager® from Aries Systems Corporation

Corresponding Author:	Lucija Andjel<br>Institute of Animal Physiology and Genetics CAS: Ustav zivocisne fyziologie a genetiky Akademie ved Ceske republiky<br>Libechov, CZECH REPUBLIC
Corresponding Author Secondary Information:	
Corresponding Author's Institution:	Institute of Animal Physiology and Genetics CAS: Ustav zivocisne fyziologie a genetiky Akademie ved Ceske republiky
Corresponding Author's Secondary Institution:	
First Author:	Stephen A. Schlebusch, PhD
First Author Secondary Information:	
Order of Authors:	Stephen A. Schlebusch, PhD
	Vladimir Trifonov, PhD
	Zuzana Halenková
	Marharyta Klianitskaya
	Dmitrij Dedukh, PhD
	Aurora Ruiz-Herrera, PhD
	Lucia Álvarez-González, PhD
	Gala Pujol
	Eva Hřibová, PhD
	Lucija Andjel
	Oldřich Bartoš
	Petr Pajer, PhD
	Tomáš Tichopád, PhD
	Daniel Kulik
	Jan Kotusz
	Marie Kaštánková Doležálková, PhD
	Astrid Böhne
	Anatolie Marta
	Patrik Horna
	Radka Reifova
	Yann Guiguen
	Heiner Kuhl
	Jan Pačes
	Karel Janko
Order of Authors Secondary Information:	

Response to Reviewers:	Dear Editors and Reviewers,<br><br>We sincerely thank you for your thorough evaluation of our manuscript entitled “Sex Chromosome Turnover and Structural Genome Divergence Shapes Meiotic Outcomes in Hybridising Cobitis.” (GIGA-D-25-00241). We appreciate the constructive feedback and the recommendation for major revision. We have carefully considered all comments and revised the manuscript accordingly to address the concerns raised. Below, we provide a point-by-point response to each comment, detailing the changes

Powered by Editorial Manager® and ProduXion Manager® from Aries Systems Corporation

implemented.

Reviewer #1 – General Comment
The authors assembled the genomes of three Cobitis species native to Eurasia in an attempt to investigate the effects of structural variants on hybrid meiotic failure. This is certainly an interesting topic given the advances in our abilities to study hybridization that have been enabled by modern genomic sequencing methods, and the evolutionary consequences of asexually-reproducing species that result from rare instances of these hybrid events.
Response:
We thank the reviewer for these appreciative words regarding the significance and relevance of our study.

Reviewer #1 – Comment 1:
The introduction of the manuscript is well-written and focused on the topic at hand. Language was mostly clear throughout the manuscript. However, the paper overall is very lengthy and would benefit from extensive revision. Personally, I think the assembly and annotation of the three genomes is worthy of being a paper (genome report) on its own. Extraction of this material into a separate manuscript would allow the authors to hone the remainder of the paper into a much more concise and focused manuscript.
Response:
We thank the reviewer for this suggestion and fully acknowledge the concern regarding manuscript length. Following the editor's guidance that separation is not required, we have retained the genome assemblies and downstream analyses within a single integrated manuscript. This decision reflects the strong interdependence between the assemblies and cytogenetic validation: confirming scaffold contiguity was essential for interpreting meiotic pairing in hybrids, and the pairing analyses gain biological meaning only in the context of the assembled chromosomal architecture. For these reasons, we believe the study is best presented as a unified whole.
Nevertheless, we took the comment seriously and substantially streamlined the Introduction, Results, and Discussion to improve the focus and reduce redundancy. Given the extent of these revisions, we do not indicate specific line changes, but the overall manuscript is now considerably more concise.

Reviewer #1 – Comment 2:
Some aspects of the methods section related to genome assembly and annotation could be clarified and/or bolstered. Presentation of methods is mostly clear, but the description of genome annotation methods is a bit tough to follow. This procedure included many complicated steps and may benefit from a flow chart, even if included only as a supplemental figure.
Response:
We thank the reviewer for highlighting the need for clearer presentation of the genome assembly and annotation workflow. To address this, we have revised these sections for improved clarity, reordered several steps for better readability, and expanded descriptions where necessary. These changes are reflected on lines 218–308 of the revised manuscript. We did not include an additional figure with a schematic flowchart because our pipeline is quite standard and should be easily followable from the Methods section.

Reviewer #1 – Comment 3:
Several important quality control steps pertaining to genome assembly and DNA/RNA sequence processing were not mentioned. Authors do not report methods used for quality filtering or trimming. They do not report any process for removal of sequencing adapters. Additionally, they do not report screening of the genome assemblies for contamination from other species. These are critical steps in producing high-quality genome assemblies that need to be addressed.
Response:
We thank the reviewer for emphasizing the importance of quality control in genome assembly. In response, we have added details on quality testing, filtering, and adapter trimming to the Methods section (lines 179 and 198;). No contamination filtering was performed, as preliminary assemblies showed a high proportion of reads mapping to the expected Cobitis sequences, indicating negligible contamination. Furthermore, all three genomes were consistent in length and content, and chromosome sizes

correlated well with cytogenetic observations, supporting the absence of significant contamination.

Reviewer #1 – Comment 4:
Presentation of statistics describing genome assembly quality, contiguity, and completeness could be improved. Authors might want to take some inspiration from statistics required for reporting in genome reports published by other journals, such as G3 or Genome Biology and Evolution. Sequencing depth is not reported in any context for the initial assemblies. Only log-transformed values are available in a single figure. Throughout the manuscript, authors conflate sequencing coverage (the proportion of a genome or genomic region that has been sequenced) with sequencing depth (the number of times a base or genomic region has been sequenced).
Response:
We thank the reviewer for pointing out these important issues. We have updated the assembly statistics to include additional metrics such as Largest Contig, N90, L90, GC content, and BUSCO gene scores. We have also corrected terminology by replacing "coverage" with "depth" where appropriate and reported sequencing depth explicitly in the relevant sections (lines 218–238; lines 241 and 256; lines 403-404). These revisions align the manuscript more closely with reporting standards used in genome-focused journals.

Reviewer #1 – Comment 5:
For the sex-linked primers designed by the authors - I would recommend development of an internal positive control that would be expected to amplify in both sexes and be easily distinguishable from the sex-linked locus by size or fluorescent label. This allows the users to distinguish between failed PCRs and identification of the homogametic sex. This is especially important because the fish selected for marker development were collected from a relatively small portion of the species' distributions (Figure 1) so there could be population-specific differences that affect reliability of these markers for identifying sex. This is a problem I regularly encounter in my own work for wide-ranging species.
Response:
We appreciate the reviewer's recommendation regarding inclusion of an internal positive control for the sex-linked PCR assay. In our study, all individuals were independently validated through standard genotyping prior to sex-marker testing: each specimen was successfully genotyped using a panel of microsatellite markers and a Sanger-sequenced nuclear locus (rps7 intron), all of which are PCR-based assays. Only individuals whose DNA consistently amplified in these established markers were included; any sample failing these tests was excluded because its genotype could not be verified. Thus, successful microsatellite and rps7 genotyping effectively served as internal confirmation of DNA quality and PCR suitability for all individuals analyzed. We have added this clarification to the Methods section ("RNA and DNA isolation and sequencing," lines 175–178).

Reviewer #1 – Comment 6:
I was also surprised that the authors did not conduct a GWAS analysis. That seems to be a fairly typical analysis included in studies of this type to elucidate sex-linked SNPs. It would add to an already extensive manuscript; however, this could add an additional argument for splitting this manuscript in two. It would provide more space to include it in a more focused manuscript.
Response:
We thank the reviewer for this suggestion and agree that GWAS can be informative in studies of sex-linked loci. However, GWAS was not feasible in our case for methodological reasons. We employed a Pool-Seq design to reduce sequencing costs and maximize genome-wide coverage across species. Because Pool-Seq aggregates DNA from multiple individuals, it does not provide individual-level genotype data required for SNP–phenotype association tests such as GWAS. Instead, our approach leverages allele-frequency differences between male and female pools, which efficiently identifies sex-linked SNPs and chromosomal regions while minimizing sampling noise typical of small individual-based datasets. This strategy revealed clear, species-specific sex-linked regions and turnover patterns that directly support our study's aims.
We acknowledge that an individual-based approach could, in principle, be applied to C. tanaitica, which was not sequenced using Pool-Seq. However, the number of

available specimens was too limited to support a statistically meaningful GWAS. For this reason, we refrained from overinterpreting results for C. tanaitica, aside from noting its non-homologous sex-determination system relative to C. elongatoides.

Reviewer #1 – Comment 7:
The results section contains many statements that would be more appropriate in the Methods section, or could be deleted entirely because they are redundant with statements already present in the Methods section. Additionally, there are some sentences that are more appropriate for inclusion in the Discussion section because they are interpretive. I have included examples under the 'Minor comments' section of this review. Some of the material presented as results in the Supplementary tables is presented in a confusing manner, and appears to contain errors (see examples in 'Minor comments' section below).
Response:
We thank the reviewer for these helpful observations. We have revised the Results and Methods sections to remove redundancies, relocated interpretive statements to the Discussion, and corrected the issues identified in the Supplementary tables as per the minor comments. These changes improve clarity and ensure that each section adheres to its intended purpose.

Reviewer #1 – Comment 8:
The first several paragraphs of the Discussion section either repeat material already covered in the Results section, or go on tangents that are not directly related to the main purpose of the paper. However, some of it could be more appropriate to include in a genome report if the authors split the manuscript in two.
Response:
We thank the reviewer for this observation. The Discussion section has been thoroughly rewritten to remove redundancies, eliminate tangential content, and improve focus. These revisions affect the entire section; therefore, we do not provide specific line references.

Reviewer #1 – Comment 9:
Given the above issues, I find that the paper needs extensive editing and possibly more analytical work (if some of the methodological deficiencies were overlooked in the analysis phase as well as the writing phase of this project). It is unlikely this work could be accomplished in the normal window for a revision. Therefore, I regrettably suggest rejection of the manuscript.
Response:
We appreciate the reviewer’s candid assessment and acknowledge the concerns raised. We have undertaken extensive revisions to address all major and minor comments, including clarifying methods, improving presentation of results, streamlining the manuscript, and correcting issues in supplementary materials. We hope that these substantial changes satisfactorily resolve the identified deficiencies and demonstrate the robustness of our analyses. We respectfully ask the reviewer to reconsider the manuscript in light of these improvements.

Reviewer #1 - Minor Comments
We thank the reviewer for the detailed minor comments, which helped improve clarity and accuracy throughout the manuscript. All suggested changes have been implemented as follows:
- Added missing version numbers for Hisat2, D-Genies, SyRI, and NGenomeSyn.
- Included additional assembly metrics (N90, L90) in Table 1.
- Corrected genome statistics and clarified the definition of “chromosome-level” assemblies; updated Supplementary Table S11 accordingly.
- Fixed terminology (e.g., replaced “coverage” with “depth”), removed redundancies, and relocated interpretive statements to the Discussion.
- Deleted repeated sentences and tangential content; streamlined Results and Discussion sections.
- Corrected typographical issues (e.g., “less” → “fewer,” removed “loci’s,” removed redundant PMER definition).
- Removed novelty claim (“the first of their kind”) as suggested.
- Standardized reference formatting and species name italicization across text, figures, and tables.
- Corrected numerical formatting in Table 2 and errors in Supplementary Tables

Powered by Editorial Manager® and ProduXion Manager® from Aries Systems Corporation

	S7–S11; added missing row descriptions.<br>•Updated figure captions and supplementary materials for clarity.<br>These revisions address all minor points raised by the reviewer.<br><br><br>Reviewer #2 – General Comment:<br>This study presents the first chromosome-level genome assemblies for three hybridising Cobitis species (C. elongatoides, C. taenia, and C. tanaitica) to investigate the genomic and cytogenetic basis of hybrid sterility and the transition to asexuality. They provide large amount of integrated data including genome scaffolding, male/female pooled sequencing (Pool-Seq), and molecular cytogenetics, and found extensive structural variation among homologous chromosomes of the three species, despite overall karyotype conservation. They further used population-level Pool-Seq analyses further revealed that each species possesses distinct, non-homologous sex chromosomes. Overall, the analyses are comprehensive and results are solid, which is suitable to this journal.<br>Response:<br>We sincerely thank the reviewer for these positive and encouraging comments regarding the scope, quality, and relevance of our study.<br><br>Reviewer #2 – Minor Comments<br>Comment 1: The Background is too long, with many short paragraphs; you can shorten it to 4–5 paragraphs.<br>Response:<br>Thank you for this comment, which also mirrors the suggestion of Reviewer #1. We have shortened and streamlined the Introduction to improve focus and readability.<br><br>Comment 2: Methods: there is no Ethics statement; please add it.<br>Response:<br>We added an Ethics statement in the dedicated section of the manuscript (lines 930-933).<br><br>Comment 3: Table 1 should be moved to supplementary files.<br>Response:<br>We moved Table 1 to the supplementary materials; it is now Supplementary Table S4.<br><br>Comment 4: Figure 4 is not easy to see.<br>Response:<br>Figure 4 has been improved for clarity and divided into two figures: the main figure is now Figure 3, and the additional detail is provided as Supplementary Figure S1.<br><br>We thank the reviewers and editors for their constructive feedback, which has significantly improved the manuscript. We believe the revised version addresses all concerns and strengthens the clarity, rigor, and impact of the study. We hope the manuscript is now suitable for publication and remain available for any further clarifications.<br><br>Sincerely,<br>Stephen Schlebusch, Vladimir Trifonov, Zuzana Halenková, Marharyta Klianitskaya, Dmitrij Dedukh, Aurora Ruiz-Herrera, Lucia Álvarez-González, Gala Pujol, Eva Hřibová, Lucija Andjel, Oldřich Bartoš, Petr Pajer, Tomáš Tichopád, Daniel Kulik, Jan Kotusz, Marie Kaštánková Doležálková, Astrid Böhne, Anatolie Marta, Patrik Horna, Radka Reifová, Yann Guiguen, Heiner Kuhl, Jan Pačes, and Karel Janko
**Additional Information:**	
**Question**	**Response**
Are you submitting this manuscript to a special series or article collection?	No
**Experimental design and statistics**	Yes

Powered by Editorial Manager® and ProduXion Manager® from Aries Systems Corporation

Full details of the experimental design and statistical methods used should be given in the Methods section, as detailed in our Minimum Standards Reporting Checklist. Information essential to interpreting the data presented should be made available in the figure legends.<br><br>Have you included all the information requested in your manuscript?	
**Resources**<br><br>A description of all resources used, including antibodies, cell lines, animals and software tools, with enough information to allow them to be uniquely identified, should be included in the Methods section. Authors are strongly encouraged to cite Research Resource Identifiers (RRIDs) for antibodies, model organisms and tools, where possible.<br><br>Have you included the information requested as detailed in our Minimum Standards Reporting Checklist?	Yes
**Availability of data and materials**<br><br>All datasets and code on which the conclusions of the paper rely must be either included in your submission or deposited in publicly available repositories (where available and ethically appropriate), referencing such data using a unique identifier in the references and in the "Availability of Data and Materials" section of your manuscript.<br><br>Have you have met the above requirement as detailed in our Minimum Standards Reporting Checklist?	Yes
GigaScience has policies and guidelines in place for the use of generative AI-writing tools such as ChatGPT. If you have used such writing tools to assist with	No

writing the manuscript this must be declared and cited in the text. Authors should not list AI-writing tools and other AI-assisted technologies as an author or co-author and should acknowledge that they are fully responsible for text generated or refined by AI-writing tools.<p><br><br>A summary of use (particularly in the introduction or among methods) needs to be included at the end of the paper, and the outputs should also be included as a supplementary file hosted in GigaDB or other open repositories. Please read our guidelines for more information. <p><br><br>By submitting to GigaScience, you are aware of the journal's AI-writing tools policy, and if you have declared use of such tools below, you have acknowledged this where appropriate in your manuscript and have made a summary of use and outputs available. <p><br><b>AI-assisted writing tools have been used in the preparation of this manuscript?	

Manuscript

Click here to access/download;Manuscript;Schlebusch_et_al_2026.docx

Sex Chromosome Turnover and Structural Genome Divergence Shapes Meiotic Outcomes in Hybridising *Cobitis*

Stephen A. Schlebusch[1,2], Vladimir Trifonov[2,3], Zuzana Halenková[1], Marharyta Klianitskaya[4], Dmitrij Dedukh[2], Aurora Ruiz-Herrera[5,6], Lucia Álvarez-González[5,6], Gala Pujol[5,6], Eva Hřibová[7], Lucija Andjel[2,8,*], Oldřich Bartoš[2,9], Petr Pajer[4,9], Tomáš Tichopád[2,10], Daniel Kulik[2,11], Jan Kotusz[11], Marie Kaštánková Doležálková[2], Astrid Böhne[12], Anatolie Marta[2], Patrik Horna[2], Radka Reifová[1], Yann Guiguen[13], Heiner Kuhl[14,15], Jan Pačes[3,*], Karel Janko[2,16,*]

Affiliations:

[1]Department of Zoology, Faculty of Science, Charles University, 12800 Prague, Czech Republic

[2]Laboratory of Non-Mendelian Evolution, Institute of Animal Physiology and Genetics, The Czech Academy of Sciences, 27721 Liběchov, Czech Republic

[3]Research Department for Limnology, University of Innsbruck, 5310 Mondsee, Austria

[4]Institute of Molecular Genetics of the Czech Academy of Sciences, 14220 Prague, Czech Republic

[5]Genome Integrity and Instability Group, Institut de Biotecnologia i Biomedicina (IBB), Universitat Autònoma de Barcelona (UAB), Cerdanyola del Vallès, 08193, Spain

[6]Departament de Biologia Cellular, Fisiologia i Immunologia, Universitat Autònoma de Barcelona (UAB), Cerdanyola del Vallès, 08193, Spain

[7]Institute of Experimental Botany of the Czech Academy of Sciences, Centre of the Region Haná for Biotechnological and Agricultural Research, 77900 Olomouc, Czech Republic

[8]Department of Ecology, Faculty of Science, Charles University, 12800 Prague, Czech Republic

[9]Military Health Institute, 16200 Prague, Czech Republic

[10]University of South Bohemia in České Budějovice, Faculty of Fisheries and Protection of Waters, South Bohemian Research Centre of Aquaculture and Biodiversity of Hydrocenoses, 38925 Vodňany, Czech Republic

[11]Museum of Natural History, University of Wrocław, 50335 Wrocław, Poland

[12]Centre for Molecular Biodiversity Research, Leibniz Institute for the Analysis of Biodiversity Change, Museum Koenig Bonn, 53113 Bonn, Germany

[13]INRAE, LPGP, 35000 Rennes, France

[14]Leibniz-Institute of Freshwater Ecology and Inland Fisheries, 12587 Berlin, Germany

[15]Ecotoxicological Laboratory, German Environment Agency, 12307 Berlin, Germany

[16]Department of Biology and Ecology, Faculty of Science, University of Ostrava, 70103 Ostrava, Czech Republic

*Correspondence address. Jan Pačes, Institute of Molecular Genetics of the Czech Academy of Sciences, 14220 Prague, Czech Republic, E-mail: hpaces@img.cas.cz; Karel Janko, Laboratory of Non-Mendelian Evolution, Institute of Animal Physiology and Genetics, The Czech Academy of Sciences, 27721 Liběchov, Czech Republic, E-mail: janko@iapg.cas.cz; Lucija Andjel, Laboratory of Non-Mendelian Evolution, Institute of Animal Physiology and Genetics, The Czech Academy of Sciences, 27721 Liběchov, Czech Republic, E-mail: andjell@natur.cuni.cz. .

Stephen A. Schlebusch [0000-0003-2355-2652];Vladimir Trifonov [0000-0003-0454-8359]; Zuzana Halenková [0000-0002-8550-1693]; Marharyta Klianitskaya [0009-0000-8631-2838]; Dmitrij Dedukh [0000-0002-1152-813X]; Aurora Ruiz-Herrera [0000-0003-3868-6151]; Lucia Álvarez-González [0000-0001-8154-8614]; Gala Pujol [0000-0002-3915-4766]; Eva Hřibová [000-0002-6868-4344]; Lucija Andjel [0000-0002-5708-1433]; Oldřich Bartoš [0000-0001-5441-5592]; Petr Pajer [0000-0002-8706-9371]; Tomáš Tichopád [0000-0002-9154-2969]; Daniel Kulik [0000-0003-1268-5409]; Jan Kotusz [0000-0001-6229-7610]; Marie Kašťánková Doležálková [0000-0002-7002-6201]; Astrid Böhne [0000-0002-1284-3115]; Anatolie Marta [0000-0002-4457-8838]; Patrik Horna [0000-0001-7337-5899]; Radka Reifová [0000-0001-5852-5174]; Yann Guiguen [0000-0001-5464-6219]; Heiner Kuhl [0000-0001-7623-9227]; Jan Pačes [0000-0003-3059-6127]; Karel Janko [0000-0002-7866-4937]

Abstract

Background: Hybridisation between divergent species can result in meiotic aberrations and the emergence of asexual reproduction. Yet, it remains poorly understood to what extent such outcomes arise from genome-wide incompatibilities versus more specific conflicts among individual chromosomes inherited from parental species, including their ability to pair during meiosis in hybrids. It is also unclear how interspecific hybrids cope with differences in sex determination systems, particularly in the context of increased ploidy. Addressing these questions requires high-quality, chromosome-level reference genomes of the parental species involved in hybrid formation.

Findings: Here, we present the first chromosome-level genome assemblies for three hybridising *Cobitis* species (*C. elongatoides*, *C. taenia*, and *C. tanaitica*), providing a comprehensive framework for investigating the genomic and cytogenetic basis of hybrid sterility and the transition to asexuality. By integrating genome scaffolding, male/female pooled sequencing (Pool-Seq), and molecular cytogenetics, we uncover extensive structural variation among homologous chromosomes of the three species, despite overall karyotype conservation. Population-level analyses revealed that each species possesses distinct, non-homologous sex chromosomes, highlighting rapid sex chromosome turnover in this recently

diverged lineage. Finally, the design of chromosome-specific painting probes, which we applied to meiotic metaphase I spreads of diploid hybrids. This approach revealed striking differences in the pairing success of orthologous chromosomes.

Conclusions: Our results demonstrate that individual orthologous chromosomes differ markedly in their ability to form bivalents during meiosis in hybrids, indicating that hybrid meiotic behaviour is shaped by chromosome-specific incompatibilities rather than uniform genome-wide failure. We also found that even closely related parental species possess distinct, non-homologous sex chromosomes, highlighting rapid turnover of sex determination systems in hybridising lineages. Together, these findings provide a high-resolution genomic and cytogenetic framework to explore how the architecture of inherited parental genomes influences sex-specific reproductive outcomes in hybrids—ranging from male sterility to the establishment of fertile, clonally reproducing female lineages—and how such asymmetries may contribute to the emergence of asexuality in vertebrates.

Keywords: speciation, asexual reproduction, polyploidy, hybrid sterility, loaches, sex determination, chromosome evolution, chromosome-specific incompatibilities

Background

Reproduction in metazoans primarily occurs through the fusion of reduced gametes produced by meiotic divisions involving recombination. However, reproductive modes vary widely among taxa, and even meiosis and recombination frequencies may be optimised for specific genomic regions, environments, or sexes [1–4]. When hybrids form between (sub)species, accumulated genetic incompatibilities and genomic structural variants (SVs) may affect hybrid fertility through meiotic impairment [5,6]. Studies in model systems further show that these meiotic problems are not evenly distributed across the karyotype: individual chromosomes differ in their ability to synapse and recombine properly in hybrids, so that only a subset of linkage groups makes a disproportionate contribution to hybrid sterility [7–9].

In extreme cases, such a merging of diverged genomes and gene regulatory networks can even lead to the abandonment of sexual reproduction due to the production of unreduced gametes, essentially resulting in a non-recombinant asexual reproduction mode (e.g., [10]).These so-called 'asexual' lineages are scattered across the tree of life and employ a wide spectrum of independently arisen cytological mechanisms for gamete production, ranging from completely ameiotic processes (apomixis) to those involving altered versions of meiotic divisions (automixis) [11,12]. Yet, despite the great variability of asexual organisms and their polyphyletic origins, recent research has identified several convergent patterns. For example, the abandonment of sex frequently coincides with interspecific hybridisation and is correlated with increasing divergence between the hybridising sexual species [13,14]. Many "asexual" hybrids produce unreduced gametes through a similar cytological mechanism—premeiotic endoreplication (PMER)—in which the maternal genetic material is duplicated prior to meiosis. As a result, recombination occurs between identical sister chromosomes, supposedly leading to no genetic variability among the progeny, apart from *de novo* mutations [15–17]. This process appears to be sex-specific and is typically confined to females, whereas hybrid males from the same crosses are usually unable to produce clonal gametes [18,19].

Recent advances in genomics have motivated studies on how the lack of effective recombination in asexual lineages shapes genome structure and gene expression. Several interesting patterns, consistent across independently arisen lineages, have been found. For example, in hybrid asexual lineages, genome evolution is not characterised by simple stasis. Instead, genomes inherited from divergent sexual ancestors may undergo gradual intragenomic restructuring, most notably through gene conversion–mediated loss of heterozygosity (LOH) [20–22]. These processes involve the non-reciprocal rewriting of one orthologous allele by its counterpart and appear distributed non-randomly across the genome and are associated with expression level, base composition, and gene function [20], suggesting that they may contribute to the adaptive optimisation of regulatory networks in hybrid genomes [23]. A largely unexplored question is whether the propensity for such intragenomic restructuring is uniform across the karyotype, or whether it reflects inherited differences among orthologous chromosomes themselves—similar to the non-random, chromosome-specific contribution to meiotic failure observed in sexual hybrids.

Thus, understanding genome evolution under restricted recombination—especially in asexual organisms—is a dynamic and challenging field. Robust comparative analyses between asexual lineages and their direct sexual ancestors are essential, yet many lineages still lack well-assembled and annotated genomes for both them and their sexual ancestors [18,24–26]. Acquiring these high-quality reference genomes is essential for addressing these questions.

The spined loaches of the genus *Cobitis* are appealing models for understanding the link between speciation, hybridisation, sex, and asexuality. Across Eurasian hybrid zones, hybridization between sexual species that diverged between 1–15 million years ago yields contrasting outcomes based on the parental species' relatedness (Figure 1). Closely related species typically form fertile and sexually reproducing hybrids [14,25]. In contrast, distantly related species generate hybrids with stark sexual asymmetry: hybrid males are normally sterile due to chromosomal mispairing disrupting meiosis, while hybrid females technically retain fertility, but only through the production of clonal eggs via PMER [14,16]. The basis for this asymmetry is unclear, but transplantation experiments show that sterile hybrid males' spermatogonia can undergo PMER in a female gonadal environment, suggesting a critical role for the maternal environment in enabling clonal reproduction [19]. These asexual all-female lineages have been repeatedly originating by interspecific hybridization throughout the Pleistocene, resulting in a diverse contemporary array of clonal strains, some of which being several hundred thousand generations old [27]. They tend to conserve their inherited parental karyotype structure without significant restructuring [28] but are subject to a gradual loss of heterozygosity which accumulates selectively in certain loci, depending on the relative transcription of the alleles [20].

This raises a central question: is genome evolution in hybrid and asexual lineages driven mainly by the passive accumulation of mutations under restricted recombination, or is it instead shaped by selection acting on specific genomic divergences, inherited among parental chromosomes? Such divergences may include differences in chromosome structure, pairing compatibility, sex determination systems, repetitive element dynamics, and regulatory networks, all of which can contribute unequally to meiotic success or failure in hybrids, asexuals and polyploids [19,29].

The aim of this study is to generate high-quality, chromosome-level genome assemblies for three parental *Cobitis* species—*C. elongatoides* (NCBI: txid166482), *C. taenia* (NCBI: txid98395), and *C. tanaitica* (NCBI: txid196115)—which serve as the sexual progenitors of various asexual hybrid lineages [14,25]. These assemblies provide a necessary foundation for investigating the genomic basis of hybrid sterility and asexuality, focusing on four key aspects: (i) divergence in repeat element content and dynamics across lineages; (ii) the extent and nature of SVs accumulated between species and their impact on chromosomal compatibility; (iii) chromosomal pairing behaviour in hybrid males as a mechanistic insight into meiotic failure; and (iv) identification and comparative analysis of genetic sex determination systems.

Methods

Sample collection

For this work, we used specimens from three *Cobitis* species: *C. elongatoides* (RRID: NCBITaxon_166482), *C. taenia* (RRID: NCBITaxon_98395), and *C. tanaitica* RRID: NCBITaxon_196115). All individuals, including the investigated hybrids, were derived from laboratory strains originally established from natural populations. These strains were maintained at the breeding facilities of the Institute of Animal Physiology and Genetics of the Czech Academy of Sciences under permit 16OZ2636/202-18134 MZe-24154/2021-18134 (see Supplementary Table S1 for detailed individual information and Figure 1 for collection sites). Specimens were assigned to taxonomic units using published microsatellite markers. and their ploidy determined by flow cytometry and karyotype verified by standard cytogenetic means as previously described [27].

RNA and DNA isolation and sequencing

A phenol/chloroform extraction protocol [30] was used to extract DNA from ~1 g of skeletal muscle for Oxford Nanopore (ONT) sequencing from one individual per species and Illumina sequencing from a second individual. DNA quality and quantity was assessed using a Qubit double-stranded DNA HS Assay Kit (Invitrogen, Thermo Fisher Scientific), agarose gel electrophoresis and an Agilent Bioanalyzer 2100 (Agilent Technologies, RRID: SCR_018043). Every specimen included in this paper was genotyped by a suite of PCR-based methods, including microsatellite screening and Sanger sequencing of nuclear (the *rps7* intron) and mitochondrial (cytochrome b) markers, to verify the taxonomy as described in [27,31].

For *C. taenia*, DNA was used for Oxford Nanopore Technology (ONT) sequencing library preparation using the 1D Genomic DNA by ligation kit (SQK-LSK108) and the library was run on MinION (RRID: SCR_017985) device using the FLO-MIN107 R9 Flow Cells according to manufacturer's instructions. For *C. elongatoides* and *C. tanaitica*, which were analysed later, libraries for ONT sequencing were prepared using a ligation sequencing kit (SQK-LSK109, Oxford Nanopore) and sequenced on a Nanopore GridION Mk1 (RRID: SCR_017986) instrument (FLO-MIN106 flow cell) according to manufacturer's instructions.

Raw Nanopore reads were filtered based on length using a minimum threshold of 1,000 bp to remove short, low-information fragments prior to assembly. No additional quality-based filtering was applied to preserve read length and maximize assembly contiguity. Illumina reads were processed using Trimmomatic v0.33 (RRID:SCR_011848) [32] with standard parameters for adapter removal and quality trimming. Bases with a Phred score below 20 were trimmed from read ends and reads shorter than 50 bp after trimming were discarded. No contamination filtering was performed, as preliminary assemblies showed a high proportion of reads mapping to the expected *Cobitis* sequences, indicating negligible contamination. Illumina sequencing was obtained with Illumina NextSeq 2000 platform (RRID: SCR_023614) according to manufacturer's instructions using NextSeq 1000/2000 P1 XLEAP-SBS Reagent Kit. This generated paired-end reads with a length of 2 × 250 bp. Raw sequencing data were obtained in FASTQ format and subjected to quality control and downstream bioinformatic analyses.

For Hi-C, a spleen was dissected from the same specimen used for ONT sequencing. The tissue was shipped on dry ice to Dovetail Genomics (*C. taenia*) or the Institute of Applied Biotechnology (IAB) (*C. elongatoides, C. tanaitica*) for OMNI-C library construction.

To investigate putative sex determination in the studied species, we sequenced the whole genomes of additional individuals. Genomic DNA (gDNA) was isolated from 122 male and female specimens of *C. taenia* and *C. elongatoides* using the DNAeasy Blood&Tissue kit (Qiagen). For each species, the isolates were pooled equimolarly into four libraries based on sex and geographical origin (see Supplementary Table S1) and sequenced using 150 bp paired-end Illumina sequencing by the IAB company. Due to the limited availability of *C. tanaitica* specimens, gDNA was isolated from additional three females and two males, and these individuals were sequenced individually.

To obtain mRNA data for gene annotation, total RNA was extracted from brain and gonadal tissues of several individuals of *C. elongatoides, C. taenia,* and *C. tanaitica* using the TRIzol protocol [33]. Sequencing libraries were prepared using the Lexogen SENSE Total RNA-Seq Library Prep Kit and were sequenced on an Illumina NextSeq 550 platform in 75 bp in paired-end mode. These newly generated transcriptomic data were combined with previously published data [23] for comprehensive gene annotation.

Genome assembly

The three genome assemblies were primarily based on Oxford Nanopore sequencing of a single male individual from each species. A hybrid approach was used for *C. taenia*, where its Nanopore reads (depth 32x) were combined with Illumina short-read data. This was done by initially assembling the short reads with *de Brujn* assemblers (about 50x depth). In order to be conservative and minimise misassemblies, the short reads were assembled with ABySS v2.0 (RRID: SCR_010709) [34] and SOAPdenovo v2.04 (RRID: SCR_010752) [35]. Long contigs which were not found in both assemblies were split into their component parts. This resulted in a more fragmented assembly, but one in which there was a consensus between the two assembly methods that the contigs were accurate. The contigs from this initial consensus assembly were then combined with the nanopore reads and assembled using Flye4 v2.9.1 (RRID: SCR_017016) [36], followed by Nanopolish v0.13.1 (RRID: SCR_016157)

(Loman et al., 2015). The assembly was then improved by two runs of Pilon v1.24 (RRID: SCR_014731) [37] using the Illumina sequencing data. Because we had Illumina reads from several individuals, we normalised the number of reads per individual and mapped them on the polished assembly using bwa v0.7.17 (RRID: SCR_010910) [38] before variant calling with Bcftools v1.10.2 (RRID: SCR_005227) [39]. Each SNP was then modified to the major allele if necessary (custom script). In contrast, the *C. tanaitica* and *C. elongatoides* genomes were assembled directly from Nanopore reads (depth 29x and 35x respectively) using Flye, followed by the same downstream polishing and variant-calling pipeline (Nanopolish, Pilon, and Bcftools; Illumina data approximately 50x depth) without an initial *de Bruijn* graph-based consensus step.

Chromosome-level de novo assembly of Cobitis genomes

Primary assemblies (N50 ~150 Kbp) were then scaffolded to a chromosomal level using Hi-C data while following the Juicer-3D-DNA pipeline v201008 (RRID: SCR_017226) [40]. The Hi-C reads for the three species were mapped against their respective fragmented draft genome using bwa [38] and reads with a MAPQ < 30 were discarded. Based on the contact frequencies, 3D-DNA (RRID: SCR_017227) was run with default parameters to construct the final superscaffolds [40]. Final assembly stats were calculated with the script stats.sh included in the sequence-analysis package BBmap (RRID: SCR_016965) [41]. Hi-C matrices were built at 500 Kbp resolution by remapping Hi-C reads against the final assembly using Juicer with default parameters. Small scaffolds were discarded and only chromosome-level superscaffolds (> 30 Mbp), organised by size, were included. Lastly, matrices were normalised, corrected, and plotted using 'hicNomalize', 'hicCorrect' and 'hicPlotMatrix' from HiCExplorer (v.3.7) (RRID: SCR_022111) [42]. First eigenvector values were calculated using the tool 'fanc compartments' from the HiC analysis tools package, FAN-C (v0.9.1) [43]. Finally, topologically associated domains (TADs) were detected with the tool 'hicFindTADs' from HiCExplorer (v.3.7) [42]. For both analyses, normalised 50 Kbp matrices were employed as input, as previously described [44].

Gene annotation

Ab initio gene prediction was performed by Dovetail as follows: repeat families found in the genome assembly of *C. taenia* were identified *de novo* and classified using the software package RepeatModeler v2.0.1 (RRID: SCR_015027) [45]. RepeatModeler depends on the programs Recon v1.08 (RRID: SCR_021170) [46] and RepeatScout v1.0.6 (RRID: SCR_014653) [47] for the *de novo* identification of repeats within the genome. The custom repeat library obtained from RepeatModeler was then used to discover, identify and mask the repeats in the assembly using RepeatMasker v4.1.0 (RRID: SCR_012954) [48]. Coding sequences from *Triplophysa tibetana* (RRID: NCBITaxon_1572043), *Astyanax mexicanus* (RRID: NCBITaxon_7994), and *Danio rerio* (RRID: NCBITaxon_2593261) (v. 2020) were used to train the *ab initio* gene prediction models in Augustus v2.5.5 (RRID: SCR_008417) [49] and Snap v2006-07-28 (RRID: SCR_007936) [50], with the Augustus model being optimised across six runs. RNAseq reads (Supplementary Table S1) were then mapped onto the genome using STAR v2.7 (RRID: SCR_004463) [51] and intron hints generated with the

bam2hints tools within Augustus [52–54]. Gene prediction was then done on the repeat masked *C. taenia* genome with Maker (RRID: SCR_005309) [55], with Snap and Augustus (with intron-exon boundary hints provided from RNA-Seq) conducting *ab initio* gene prediction as part of this pipeline. To help guide the prediction process with Maker, Swiss-Prot peptide sequences from the UniProt database (v. downloaded in 2020) (RRID: SCR_002380) [56] were used in conjunction with the aforementioned protein sequences from *T. tibetana*, *A. mexicanus,* and *D. rerio*. *Ab initio* genes were only kept in the annotation if they were predicted by both Snap and Augustus. To help assess the quality of the gene prediction, AED scores were generated for each of the predicted genes as part of the Maker pipeline. Genes were further characterised for their putative function using NCBI Blastx (RRID: SCR_004870) [57] search of the peptide sequences against the UniProt database. tRNAs were predicted using tRNAscan-SE v2.05 (RRID: SCR_008637) [58].

Initial gene prediction metrics at this point however showed that the annotation was not of high enough quality, with 35,082 genes and only 74.9% of BUSCO genes found to be complete (with 5.9% partially found and 19.2% were missing). This was lower than the 95% of BUSCO genes found by BUSCO (RRID: SCR_015008) if the analysis was done directly on the genome.

The annotation was improved by extending the transcripts and generating new ones using StringTie v2.2.1 (RRID: SCR_016323) [59], followed by Transdecoder v5.7.1 (RRID: SCR_017647) [60]. Our analysis incorporated published [23,25] as well as newly obtained mRNA data from muscle, liver, gonad, and spleen tissue from *C. taenia* and *C. elongatoides* for annotation (Supplementary Table S1). Mapping was done by STAR v2.7.10b. Dovetail's annotation was then merged with this annotation from StringTie using Another Gtf/Gff Analysis Toolkit (AGAT) (RRID: SCR_027223) [61]. Finally, we deleted genes that were identified as being repetitive elements. The new combined annotation had 93.3% of BUSCO genes complete, with 2.3% fragmented and 4.4% missing genes.

The *C. tanaitica* and *C. elongatoides* genomes were annotated using HANNO v0.5 [62]. Species specific RNAseq data were mapped to the relevant *Cobitis* genome using Hisat2 v2.2.1 (RRID: SCR_015530) [63] and transcript models were built using StringTie v2.2.1 [59]. The resulting transcript GTF from this initial run was added to the HANNO v0.5 pipeline (parameter -g) alongside RefSeq protein (-p) (RRID: SCR_003496) and mRNA evidence (-r) from *M. anguillicaudatus* (GCF_027580225.1) (RRID: NCBITaxon_75329) and *P. dabryanus* (GCF_030506205.1) (RRID: NCBITaxon_1515643). A second run of HANNO was performed without RefSeq mRNAs, which successfully identified a few missing genes which were added to the results from the first run. This resulted in 88.6% complete BUSCO genes being found for *C. elongatoides* (with 5.2% fragmented and 6.2% missing) and 90.9% complete genes found for *C. tanaitica* (with 4.1% fragmented and 5.0% missing).

Repetitive element annotation

In order to identify and annotate the repetitive elements, initial consensus sequences were generated using the Dfam TETools container v1.87 [64] (running on Docker 24.0.5), which packages RepeatModeler v2.0.5 and RepeatMasker v4.1.5 together with Dfam 3.7 (RRID: SCR_021168) (curated portion only).

We ran three RepeatModeler runs on each of the base genome assemblies (including the unplaced contigs). The resulting consensus sequences from the three species were then combined with curated families from Dfam v3.7 to form a single library.

To remove redundancy in the resulting library, we used Blastn v2.11.0+ and compared the library against itself with a word size of 20 and a minimum percentage identity of 95%. Overlapping sequences were either joined to form a new consensus or one of them shortened to remove the overlap. This was run iteratively until there were no more segments to remove. RepeatMasker was run on each genome with this reduced repeat library, which was further refined by removing portions of each sequence in the library which only aligned to the genomes once. Finally, RepeatMasker was run on each assembly using the library from the secondary refinement.

Identification of structural variants

The assembled genomes of *C. elongatoides* and *C. tanaitica* were mapped to the *C. taenia* reference genome using minimap2 v2.24 (RRID: SCR_018550) [65] (parameters -ax asm10 --eqx). Homology between species was analysed using dot plots generated by D-Genies version 1.5.0 (RRID: SCR_018967) [66]. SyRI version 1.6.3 (RRID: SCR_023008) [67] was used to distinguish syntenic and rearranged blocks and to identify SVs (fusions, fissions, translocations, inversions, and duplications). SyRI was run independently for *C. elongatoides* and *C. tanaitica*; both analyses used the *C. taenia* genome assembly as reference. To meet the requirement of having the same number of chromosomes for all species (a requirement by SyRI identification software), Ch01A and Ch01B of *C. elongatoides* and *C. tanaitica* were combined using a 1 Kbp long spacer prior to the analyses. The coordinates of the identified structures were then transferred back to Ch01A and Ch01B.

Due to SyRI producing many short tandem events for translocations and duplications rather than one long rearrangement, all neighbouring blocks of length over 5 Kbp of the same structure type and orientation were merged to be considered a single rearrangement. This approach results in a more parsimonious set of changes in chromosomal structure.

Syntenic blocks and SVs were visualised using NGenomeSyn version 1.41 [68]. For visualization purposes, only structures spanning more than 5 Kbp were considered. Gene synteny analysis was produced using python JCVI package v1.4.16 (RRID: SCR_021641) [69]. Intersections of repeat annotations and indels were produced using Bedtools v2.31 (RRID: SCR_006646) [70]. The genomic positions of previously reported satellite DNA arrays (>5 Kbp) [71] were assigned to chromosome contigs using BLAST.

Mitotic and meiotic chromosome preparation

In order to visualise chromosomal pairing, adult *C. elongatoides*, *C. tanaitica,* and *C. taenia* males and females as well as *C. elongatoides* x *C. taenia* hybrid males were injected with 0.1% colchicine solution (1 mL/100 g of body weight). Mitotic and meiotic metaphase chromosome spreads were obtained from kidneys and testes according to previously

published protocols [28,71]. Briefly, kidneys and testes were removed, dissected in 0.075 M KCl to release cells and treated hypotonically for 30 min at room temperature. After centrifugation, cells were fixed in freshly prepared methanol: acetic acid (3:1) fixative and washed twice in a new portion of fixative. The fixed cell suspension was then dropped onto slides. Mitotic and meiotic metaphase chromosomes were initially stained with Giemsa to assess chromosomal number and morphology.

Single chromosome Oligo-FISH probe design and Chromosomal painting

Oligomers specific to chromosomes 5 (Ch05) and 20 (Ch20) as well as for the long (Ch01A) and short (Ch01B) arms of chromosome 1 were designed based on the *C. taenia* assembly using Chorus software [72]. Considering the different chromosome lengths, one set of 27,000 oligomers (45-mers) was designed to visualise the whole length of Ch20, while partial regions of chromosome scaffolds Ch01A, Ch01B, and Ch05 were targeted by designed sets of oligomers specific to ensure sufficient probe coverage (Supplementary Table S2). Chromosome regions with very low oligo densities were omitted in the final probe datasets (Supplementary Table S2), which are available upon request. The final probe sets were synthesised as myTAGs® Labelled Libraries (Daicel Arbor Bioscience) and directly used for FISH experiments.

Oligoprobes to Ch20 and Ch01A were labelled with biotin, and oligoprobes to Ch05 and Ch01B were labelled with digoxigenin. Prior to hybridisation, chromosome slides were incubated with 0.01% pepsin/0.01 M HCl at room temperature for 10 min and fixed with 2% paraformaldehyde for 10 min. For two-colour FISH we mixed oligoprobes to Ch05 and Ch20 or Ch01A and Ch01B (50 ng of each probe per slide) with 20 ul of hybridisation mixture (50% formamide, 10% dextran sulphate, 2× SSC, and 500 ng of salmon sperm DNA (Sigma-Aldrich). Probes were denatured at 86°C in the heating block for 10 min and then put on ice. Slides with mitotic or meiotic chromosomes were denatured in 75% formamide/2x SSC at 74°C for 5 min, dehydrated in an ice-cold series of ethanol (70%, 80%, 96%) and dried prior to denatured probe application. After hybridisation overnight at room temperature, slides were washed three times with 0.2x SSC for 5 min at 42°C and 2× SSC for 5 min at room temperature. The biotin and digoxigenin labelled probes were detected using streptavidin-AlexaFluor 488 (Invitrogen) and anti-digoxigenin-rhodamine (Invitrogen), respectively. Following three washings in 4× SSC with 0.1% Tween at 44°C for 5 min with shaking, the slides were dehydrated in an ethanol series (70%, 80% 96%), air dried, and mounted in Vectashield medium containing DAPI (1.5 mg/mL) (Vector).

Wide-field and fluorescence microscopy

Mitotic and meiotic chromosomes with chromosomal painting were inspected using Carl Zeiss Axio Imager.Z2 (RRID: SCR_018876) and Provis AX70 Olympus microscopes (RRID: SCR_020336) equipped with standard fluorescence filter sets. Microphotographs of chromosomes were captured by a CCD camera (DP30W Olympus) using Olympus Acquisition Software and CoolCube 1 using the MetaSystems platform for automatic search, capture and image processing. Microphotographs were finally adjusted and arranged in Adobe Photoshop,

CS6 software. Image processing was limited to the standard functions of the software, including contrast and brightness enhancement.

Sex Chromosome identification and validation through candidate loci PCR

Four pooled DNA samples for *C. elongatoides* and *C. taenia*, along with six individual *C. tanaitica* samples were trimmed using Trimmomatic (Illuminaclip 1:25:10, Slidingwindow 4:17, Trailing 10, Minlen 100) and aligned to their respective reference genomes using bwa v0.7.17 [38] for an average depth of 47x for the pooled data and 38x for the individual data for an average depth of 47x for the pooled data and 38x for the individual data. Pooled DNA samples were split according to sex and geographic origin of the samples, i.e., two rough geographic groupings split into two sexes resulting in the 4 pools for *C. elongatoides* (altogether 25 males and 45 females) and *C. taenia* (altogether 20 males and 32 females) respectively (see Figure 1 and Supplementary Table S1). SNPs were called from the aligned data using Gatk v4.2.3.0 (RRID: SCR_001876) [73]. Samtools v1.19.2 (RRID: SCR_002105) [39], Vcftools v0.1.16 (RRID: SCR_001235) [39] and Bedtools v2.31.1 (Quinlan & Hall, 2010) were used to calculate the depth across each genome assembly and the concentration of sex-specific SNPs.

Putative Y-chromosome specific regions were identified in the *C. elongatoides* and *C. taenia* genomes, where pooled female reads had zero depth and pooled male reads had at least 30% of their average genomic depth. Putative X-chromosome specific regions were identified in *C. elongatoides* where the pooled female reads had twice the depth as pooled male reads and within 20% of the average genomic depth. Primers were designed to these regions, which included sections of Ch01A in *C. elongatoides,* as well as Ch02 and Ch05 in *C. taenia*. In the case of *C. elongatoides*, where the identified Y-chromosome regions were larger and more numerous, PCR primers were designed with their entire range within the male specific regions. In *C. taenia*, due to a smaller relevant region, only one primer from each pair was designed within the male specific region, while the other primer was placed on the flanking regions. In total, we tested seven *C. elongatoides* X-specific primer sets, twelve *C. elongatoides* Y-specific primer sets, eighteen *C. taenia* Y-specific primer sets, and the autosomal *rps7* gene primer pair as a positive control ([74], Supplementary Table S3). To evaluate their efficacy and specificity, we conducted PCR reactions (see Supplementary Table S4 for conditions) with DNA from males and females of both species (see Supplementary Table S1 for details on the individuals used). Additionally, we tested *C. taenia* sex chromosome-specific markers on genomic DNA from six males and three females of *C. tanaitica* and did not detect any positive bands. Gel electrophoresis was performed after the PCR to confirm the amplification of single products of the expected size and to verify that the bands only appeared in expected individuals. Detailed information of three confirmed primer sets can be found in Supplementary Table S3.

Candidate Sex-determining genes

To identify potential candidate sex-determining genes in the three *Cobitis* species, we compiled a list of candidate actinopterygian master sex-determining genes based on the

literature (see Supplementary Table S5). The presence and genomic location of these genes were then determined using the gene annotation of each species and verified with Tblastn (see Supplementary Table S6).

Results

Genome assembly

The initial assemblies combined deep depth short read sequencing (*C. taenia*) and lower depth long ONT reads (all species) to get an N50 value of approximately 150 Kbp for all species. These assemblies were used to generate superscaffolds with Hi-C contact reads (see Supplementary Table S7 for comparison of assemblies before and after Hi-C interaction mapping). A total of 416 (*C. taenia),* 801 (*C. tanaitica*), and 988 million pairs *(C. elongatoides)* of Hi-C reads were employed to assemble each genome using the Juicer-3D-DNA pipeline (see methods). After filtering, a total of 109 million (*C. taenia)*, 427 million (*C. tanaitica*), and 433 million *(C. elongatoides)* unique contacts were used to assemble each species' genome (see Supplementary Table S8 and Supplementary Table S9).

Ultimately, we successfully generated chromosome-level assemblies for males of the three *Cobitis* species: *C. taenia* (n=24, where "n" is the number of chromosomes in a haploid set), *C. tanaitica* (n=25), and *C. elongatoides* (n=25); Table 1, Figure 2A) with N50 values greater than 40 Mbp, indicating high-quality and well-scaffolded genomes. The final genome sizes were 1.6 Gbp for *C. taenia*, 1.7 Gbp for *C. tanaitica*, and 1.8 Gbp for *C. elongatoides*. The majority of each genome, namely 85.8% in *C. taenia*, 82.2% in *C. tanaitica* and 68.4% in *C. elongatoides*, was organised into chromosome-level superscaffolds corresponding in number to the described diploid numbers (2n=48 in *C. taenia*, and 2n=50 in both *C. elongatoides* and *C. tanaitica*) [31].

Chromosome-level scaffolds were named based on their length in the *C. taenia* genome, from Ch01 (the largest) to Ch24 (the smallest) (Supplementary Table S10). This nomenclature does not correspond to previously published classifications based on chromosome morphology (e.g., [31,71]). The same naming convention and orientation were applied to the inferred homologous chromosomes in the other two species. In those species, the scaffolds representing the ancestral syntenic regions of the recently fused chromosome Ch01 of *C. taenia* were named Ch01A and Ch01B.

A/B compartments and topologically associated domains (TADs)

Comparison of Hi-C matrices revealed similar patterns of chromosomal interactions amongst the species (Figure 2). The detection of 3D structures, such as compartments and TADs, showed consistent patterns in the three species (Figure 2A), mirroring previous observations in other vertebrates [44,75]. The genome-wide distribution of A/B compartments was similar across taxa, with ≈50% of the genome was detected as A compartments ("open" chromatin), and no major differences were observed on compartment strength between species (Figure 2B). TADs exhibited the same trends. In the three species, TADs of ≈0.8 Mbp

(Supplementary Table S11) were defined with equal insulation capacity (Figure 2C, 2D). Overall, our results suggest a high level of chromatin structure conservation among *Cobitis* species.

Gene annotation

For *C. taenia* we identified 31,513 genes. BUSCO analysis using actinopterygii_odb10 (RRID: SCR_011980) (3,640 BUSCOs) showed 95.6% (C:93.3%; F:2.3%) coverage (BUSCO v3.0.2, ODB v10, hmmsearch v3.4). Both other genomes give slightly worse results, possibly due to fewer contigs placed in chromosome-level scaffolds, with *C. elongatoides* having 42,868 genes with a BUSCO coverage of 93.8% (C:88.6%; F:5.2%) and *C. tanaitica* having 40,250 genes and a BUSCO coverage of 95.0% (C:90.9%; F:4.1%). The number of genes per chromosome and the proportion of scaffolded versus unscaffolded parts of the genome are given in Supplementary Table S10.

Repetitive element annotation

Overall, approximately 54-55 % of the assembled *Cobitis* genomes were identified as being repetitive elements. This includes 9,928 repeat classes and 90 repeat families in *C. taenia*, 9,882 repeat classes and 90 repeat families in *C. elongatoides*, and 9,924 repeat classes and 91 repeat families in *C. tanaitica*, excluding simple, low complexity, and satellite repeats.

Most families are equally distributed (Figure 3), but several families are expanded only in one or two species. A Kimura distance-based copy divergence analysis demonstrated that the closely related *C. taenia* and *C. tanaitica* have very similar TE content with clear evidence of an on-going expansion in some families, while *C. elongatoides* differs from both with indications of a recent decline in TE activity (Figure S1).

Inference of homology and structural variants

After aligning the genomes, a clear one-to-one alignment pattern was observed along the entire length of 23 out of 24 chromosomes in *C. taenia* and 25 chromosomes in *C. tanaitica* and *C. elongatoides* (Figure 4A, 4B; Supplementary Figure S2, S3). In contrast, chromosome 1 (Ch01) of *C. taenia* aligned to two separate chromosomes (Ch01A and Ch01B) in both *C. tanaitica* and *C. elongatoides*.

The proportion of syntenic regions between homologous chromosomes ranged from 24 to 68% in the comparison of *C. tanaitica* to *C. taenia* and from 17 to 49% in the comparison of *C. elongatoides* to *C. taenia*. We identified a total of 35,633 (SVs) in the *C. taenia-C. tanaitica* comparison and 41,228 in the *C. taenia-C. elongatoides* comparison (Supplementary Table S12). Among the longest rearrangements, we detected 53 and 66 inversions spanning over 1 Mbp in the *C. taenia-C. tanaitica* and *C. taenia-C. elongatoides* comparisons, respectively. Other types of rearrangements, such as duplications and translocations, were

often scattered as adjacent shorter events, which could be better explained by a single larger event. To address this, we applied our parsimony merging procedure (see Methods), resulting in a total of 69 rearrangements in the *C. taenia-C. tanaitica* comparison, and 116 rearrangements in the *C. taenia-C. elongatoides* comparison, each spanning over 1 Mbp (Supplementary Figure S3, Supplementary Table S13). These extensive rearrangements include large translocations in Ch11 and Ch04 in *C. tanaitica*, with approximately one-third of the chromosome spanned by inverted translocations (Figure 4B).

Identified insertions and deletions spanned a total length of 7.6 Mbp and 2.8 Mbp in the *C. taenia-C. tanaitica* and *C. taenia-C. elongatoides* comparisons respectively. Of these indels, approximately 73% were composed of repeats in the *C. taenia* - *C. tanaitica* comparison and 71% in the *C. taenia-C. elongatoides* comparison. Among the most represented classes of repeats among the indels were DNA hAT-Ac elements (9% in *C. taenia-C. tanaitica*, 4.8% in *C. taenia-C. elongatoides*) and long terminal repeat (LTR) Gypsy elements (10.6% in *C. taenia-C. tanaitica*, 12.4% in *C. taenia-C. elongatoides*). Detected intrachromosomal rearrangements were confirmed using gene synteny analysis (Figure 4A).

Additionally, we observed several interchromosomal events, 12 in the *C. taenia* - *C. tanaitica* comparison, 50 in the *C. taenia-C. elongatoides* comparison and 100 in the *C. tanaitica-C. elongatoides* comparison. These interchromosomal events span on average 8 genes in *C. taenia-C. tanaitica*, 11 genes in *C. taenia-C. elongatoides* and approximately 9 genes in *C. tanaitica-C. elongatoides*. The longest detected rearrangements (spanning over 40 genes) from the *C. taenia-C. elongatoides* comparison are located on Ch05, Ch19, Ch22, and Ch23 in *C. elongatoides* and Ch02, Ch03, Ch10, and Ch22 in *C. taenia*. Interchromosomal rearrangements between these pairs of chromosomes are also the longest ones in the *C. tanaitica-C. elongatoides* comparison.

Validation of Chromosome Structures Through Chromosome Painting

Chromosome painting was performed using probes designed based on the genome assembly of *C. taenia*. These included probes covering the entire scaffold Ch20, a part of Ch05, as well as two regions of Ch01 corresponding to collinear parts of Ch01A and Ch01B of *C. tanaitica* and *C. elongatoides*. These paintings confirmed the accuracy of our genome assemblies across four selected linkage groups in all three sexual *Cobitis* species.

Specifically, the probe of Ch05 highlighted the distal part of the q-arm of a large submetacentric chromosome across all species, confirming the structural integrity and assembly accuracy of this chromosome scaffold. The probe of Ch20 labelled the q-arm of small subtelocentric chromosomes in *C. taenia* and *C. elongatoides*, suggesting a conserved structure in these species. Conversely, in *C. tanaitica*, the probe signal was observed in the q-arm of a small acrocentric chromosome (Figure 5), suggesting morphological differences. Finally, the application of probes for linkage groups Ch01A and Ch01B in *C. elongatoides*, when applied in *C. taenia*, showcased distinct hybridisation signals on the short and long arms of the largest metacentric chromosome with its centromeric region unstained and exhibiting only DAPI signal (Figure 5). This confirmed a fusion event unique to this species. In *C. tanaitica,* the Ch01A and Ch01B probes highlighted two pairs of subtelocentric chromosomes,

while in *C. elongatoides*, two pairs of submetacentric chromosomes, once again highlighting structural differences among the species.

Interestingly, even after the visualisation of Ch01A, we did not observe any difference in morphology and hybridisation signals between male and female mitotic chromosomes in all three studied sexual species, despite the possible role of Ch01A in sex determination of *C. elongatoides* and the identified divergence between Y- and X-specific sequences. This suggests that differentiation of the XY chromosomes is limited to nucleotide substitutions and other small rearrangements rather than large structural changes.

In diploid *C. elongatoides-taenia* hybrids (ET in Figure 5), chromosome painting verified the presence of orthologous Ch05 and Ch20 corresponding to those identified in *C. elongatoides* and *C. taenia*. Further, the Ch01A and Ch01B probes revealed the fused *C. taenia*'s chromosome Ch01 alongside the distinct submetacentric chromosomes from *C. elongatoides* (Figure 5).

Meiotic Chromosome Pairing in Hybrids and Pure Species

Chromosome painting with Ch05 and Ch20 probes applied to meiotic metaphase I spermatocyte spreads of all three parental species showed the presence of one larger bivalent corresponding to Ch05 homologs and a smaller bivalent corresponding to Ch20 homologs (Supplementary Figure S4). In addition, meiotic metaphase I of *C. taenia* showed both Ch01A and Ch01B hybridisation signals on the single largest bivalent, while *C. elongatoides* and *C. tanaitica* exhibited two distinct small bivalents corresponding to Ch01A and Ch01B paired homologs, corroborating the mitotic chromosome data (Supplementary Figure S4).

To understand how the pairing patterns proceed in interspecific hybrids, we applied these chromosome-specific probes to 526 meiotic metaphase I spermatocytes from two diploid ET hybrid males (*et1c4* and *et1c5* Supplementary Table S1) (Figure 6A-6C). These males were derived from crossing of *C. taenia* mothers and *C. elongatoides* fathers and were now confirmed to have inherited the Y gametolog of Ch01A from *C. elongatoides* via positive PCR amplification signal with the Y-linked primer pair and negative amplification for the X-linked primer pair (see Supplementary Table S3).

Despite differences in the total number of metaphases inspected for each individual and chromosome (see Figure 6D for exact counts), statistical analysis revealed significant differences in pairing success among the four investigated chromosomes. A generalised linear model (GLM) with a binomial error structure was used to test the effects of chromosome identity, individual, and their interaction on pairing success (response variable: paired vs. unpaired). The model showed that chromosome identity had a highly significant effect on pairing success ($p < 0.0001$), indicating consistent interchromosomal differences in pairing likelihood.

Post-hoc pairwise comparisons, adjusted for multiple testing using the Bonferroni correction, confirmed that Ch01B paired significantly more frequently than all other chromosomes ($p < 0.0001$ for all pairwise contrasts; Figure 6D). Ch05 and Ch20 bivalents were observed in only a small proportion of cells, with both chromosomes showing significantly

lower pairing likelihoods compared to Ch01B ($p < 0.0001$ for both comparisons). Ch01A, the putative Y gametolog derived from *C. elongatoides*, exhibited the lowest pairing rates overall, appearing primarily as univalents (Figure 6D).

While these interchromosomal differences were consistent across both individuals, significant interindividual differences were observed for Ch01B. A separate GLM for Ch01B revealed that its pairing success was significantly lower in one hybrid male (*et1c5*, Supplementary Table S1) compared to the other (*et1c4*; $p = 0.0006$). However, no significant interindividual differences were detected for the other chromosomes, suggesting that variability in pairing dynamics for Ch01B might reflect unique characteristics of this chromosome or its interaction with individual-specific factors.

Identification of sex chromosomes

Pooled genomic sequencing from 24 males and 45 females from *C. elongatoides* and 20 males and 32 females from *C. taenia* were used to calculate sex-specific depth and identify sex-associated SNPs across each respective reference genome. Due to the difficulty of getting *C. tanaitica* individuals, three males and three females were sequenced individually and used for the analysis.

No visible depth differences between sexes were observed in any chromosome in *C. taenia* and *C. tanaitica* (Figure 7A, 7B). By contrast, in *C. elongatoides*, our analysis provided a clear signal across the whole Ch01A scaffold, some of which had a depth in females equal to the average genomic depth and half the depth in males, consistent with an X chromosome. Other regions of Ch01A showed half the average genomic depth in males and low depth in females, consistent with a Y chromosome (Figure 7C). This intermixed pattern suggests that *C. elongatoides* has an X/Y system and that the assembled Ch01A scaffold represents a chimeric combination of these chromosomes' sequence.

Sex-specific SNPs were identified in all three species using the pooled Illumina datasets. They appeared concentrated in several potential sex-specific regions in *C. tanaitica*, most notably a 900 Kbp region on Chromosome 6 (Figure 7E) as well as once again highlighting the whole of Chromosome 1A in *C. elongatoides* (Figure 7F). As no notable concentration of sex-specific SNPs was observed in *C. taenia* (Figure 7D), we selected regions of its genome with zero depth in females and a depth greater than 30% of the average genomic depth in males (where the Y chromosome-specific regions are expected to have an average of 50% depth). The two regions with the highest density of such regions were located on Ch02 and Ch05. PCR primers were designed to these two chromosomes such that one of the two primer pairs was within one of these putative male specific regions. The sex-chromosome specificity of primers designed to Ch05 was successfully validated using an additional 11 sexed individuals not used in the Pool-Seq data, while the Ch02 primers failed to predict the sex of the tested samples (Supplementary Table S14).

Primers were also designed to amplify regions predicted to be sex chromosome-specific (either X- or Y-specific) in *C. elongatoides* to use for future sex identification of pure individuals or identification of sex chromosome presence in interspecific hybrids. The PCR primers and their amplification conditions were tested on the individuals that were not used in

the creation of the PoolSeq library. As a criterion for primer selection for further screening we considered that primers were (1) amplifying a single band in a sex-chromosome-specific manner (i.e., Y-marker in males only and X-marker in both sexes), and (2) they amplified in a species-specific manner, hence, not making any product in non-target species. Finally, three sets of primers were selected, reliably diagnosing Ch01A Y- and X- specific loci in *C. elongatoides* and Ch05 Y-specific loci in *C. taenia* (Supplementary Table S3). A primer set that exclusively amplified the *C. taenia* X locus was not found.

From the literature (Supplementary Table S5), 41 genes (including paralogs) were found which are master sex determination or key regulators of sex-determining pathways in actinopterygians. All of these genes were mapped to chromosome-level scaffolds except four in *C. tanaitica* and nine in *C. elongatoides*, which were found on unplaced scaffolds. Notably, among these genes, *paics* (the master sex determination genes in the blue tilapia - *Oreochromis aureus*) aligned to Ch01A, the putative sex chromosome, in *C. elongatoides* (Supplementary Table S6).

Additionally, we analysed the list of genes located on Ch01A to check for the presence of any additional genes, which were considered as linked to sex differentiation in various fish species (Supplementary Table S6). We detected the presence of four such genes (*bmp2b, gata4, gpatch2,* and *gopc*) which are known to play a key role in vertebrate sex determination/differentiation and thus are potential candidates for the sex determination master gene in *Cobitis elongatoides*. Interestingly, *gata4* and *gpatch2* are located in a highly differentiated region of the X chromosome, while *gopc* and *bmp2b* reside in a moderately diverged region. *paics* is in the least diverged part. All these genes are located on the same syntenic group (autosome 20) in *D. rerio*.

Discussion

Filling the taxonomical gap in chromosome level assemblies

Recent advances in long read sequencing and chromatin capture technologies have substantially improved the feasibility of chromosome-level genome assemblies for non-model organisms. However, the data are accumulating in a taxonomically and geographically biased manner, which significantly limits large-scale comparative genomic studies by underrepresenting or omitting entire deeply diverged clades. Among Cypriniformes, a diverse and economically significant order of Old-World freshwater fishes, the NCBI Genome database lists 306 genomes as of March 2025, including 89 genomes from species of the family Cyprinidae (which has around 1,780 species in total), while the family Cobitidae, which contains approximately 260 species across the entire Palearctic, is represented by only 5 species. By producing chromosome-level genomes for three species representing the Western Cobitidae lineage—which diverged from the nearest available Asian Misgurnini species during the Oligocene epoch (~30 Mya; [76])—this study addresses an important biotaxonomic and geographic gap.

It is also worth mentioning that modern methods can still fail in assembling genomic regions enriched in repetitive content (e.g., centromeres, telomeres), and structural variants, which can lead to fragmented scaffolds that are not placed in their respective chromosomes.

It is therefore desirable to validate resulting assemblies with other means, such as cytogenetics [77,78]. In this study, despite using the up-to-date technologies, we were still unable to confidently place 14-32% of sequences in each genome. Nevertheless, our assemblies contain the expected number of chromosomes in all three species, with an N50 range of 43-53 Mbp. The application of four chromosome-specific probes (Ch01A, Ch01B, Ch05, and Ch20), newly designed form our assemblies, further confirmed the contiguity of our assemblies, and detected some genomic features, most notably the fusion of two chromosomes (Ch01A and Ch01B) into a single chromosome (Ch01) in *C. taenia,* which is in line with previous cytogenetic evidence [79,80]. Finally, the comparison to published chromosome-level assemblies suggests a high level of conservation of syntenic groups within the Cobitoidea suborder as a whole, demonstrating the 25 elements in haploid state and very few interchromosomal rearrangements.

Additionally, our assemblies—supported by Pool-Seq and chromosome-specific probe design—revealed intricate links between the evolutionary phenomena of speciation, hybridisation, asexuality, and hybrid sterility. First, they enabled the detection of divergent sex chromosomes in these reproductively isolated, yet hybridising, species. Second, they facilitated the visualization of chromosomal incompatibilities in hybrids, providing mechanistic clues to their sterility.

Accumulation of structural variants and evolution of the repeatome

The comparison of our assemblies to published Cobitoidei genomes identified at least three independent cases of fusions of two chromosome elements. Namely, it concerns the genome assemblies of *Paramisgurnus dabryanus* (2n=48, [81]) and *Oreonectes platycephalus* (2n=48, [82]), where the fusions appear to involve homologs of *Cobitis* Ch14 and Ch22 in *P. dabryanus*, and homologs of *Cobitis* Ch21 and Ch22 in *O. platycephalus* - (Supplementary Table S15). A third such event, detected in *C. taenia* between elements Ch01A and Ch01B, suggests the independent involvement of different syntenic groups across cobitoid species.

Namely, while both Ch01A, Ch01B appear as small chromosomes in *C. elongatoides* and *C. tanaitica*, they are not acrocentric, but submetacentric in *C. elongatoides* and subtelocentric in *C. tanaitica*, with p-arms highlighted by a euchromatic probe in both species. This in turn suggests that it was not a standard Robertsonian fusion into Ch01 of *C. taenia,* but also some fine-scale structural rearrangements took place. This is in line with suggested inversions and translocations along both Ch01 - Ch01A and Ch01B (see and compare Figure 4A, 4B and Figure 5), but it suggests that such accumulation of structural variants may occur on short evolutionary timescales since *C. taenia* diverged from *C. tanaitica* recently, 0.5–1.5 Mya [25].

A striking feature of *Cobitis* genome evolution is the rapid accumulation of intrachromosomal structural rearrangements, particularly the numerous pericentric and paracentric inversions. The rate of this accumulation appears to be time dependent. We observed ~41,000 SVs between *C. elongatoides* and *C. taenia* (diverged ~10 Mya; ~1 SV per 250 generations), compared to ~36,000 SVs between *C. taenia* and *C. tanaitica* (diverged ~1 Mya; ~1 SV per 28 generations). This nearly tenfold higher short-term rate may reflect the

inclusion of transient polymorphisms that are later purged by selection over longer timescales [83]. Even the genomes of two recently diverged species, *C. taenia* and *C. tanaitica*, are characterized by over 50 large (over 1 Mb) inversions (Figure 4A; Supplementary Tables S12, S13), including pericentric ones on Ch01A and Ch01B. Cytogenetic data confirm this reshuffling, showing clear changes in centromere position (Figure 5). The distribution of inversions appears non-random across the karyotype: while some chromosomes (e.g., Ch15, Ch16, Ch21, Ch24) maintain relatively conserved synteny, others (e.g., Ch01b, Ch4, Ch10) exhibit extensive rearrangement (Figure 4; Supplementary Figure S3). This chromosomal reshuffling likely influences meiotic pairing and bivalent formation in hybrids, as discussed in a later section. In contrast, the number of large interchromosomal translocations remains low, limited to the single Ch01A/01B fusion in *C. taenia* described previously.

In general, inversions are structural mutations well known for suppressing recombination in heterozygous states [84]. Recent studies have demonstrated that inversions tend to become fixed more frequently in zones of overlap between species that are not fully reproductively isolated [85]. In these cases, inversions help maintain species integrity following secondary contact. Our data contribute to this body of knowledge and suggest that similar mechanisms may be at work in sympatric fish species.

As in other cypriniform fishes, *Cobitis* exhibits relatively high transposable element (TE) content and diversity, which likely explains its large genome size of ~1.7 Gbp. This is substantially larger than in related groups such as *Triplophysa* (~500-700 Mbp) and *Beaufortia* (~450 Mbp) [86–88]. Similar to other fish genomes, *Cobitis* species are poor in SINEs [89]. The genus appears to be undergoing an ongoing expansion of both Class I and Class II transposons, where the rate of active transposition surpasses the rate of inactivation. We found evidence of a recent transpositional burst involving DNA transposons (hAT), LTR retroelements (Gypsy), and long interspersed nuclear elements (LINEs) such as L2. The LTR content in *Cobitis* genomes (17–19%) is much higher than in other cyprinoids, including *D. rerio* (5%) and *Paramisgurnus dabryanus* (7.5%) [81,88]. These recently expanded TE families could be used in future studies to detect deregulation in hybrids and to identify species-specific chromosomes or chromosomal regions.

Chromosome Pairing in Hybrids: Insights into Hybrid Sterility and Asexuality

Understanding how divergence between orthologous chromosomes affects their meiotic behaviour in hybrids is central to explaining key evolutionary phenomena such as hybrid sterility and the formation of reproductive barriers. Theory predicts that individual chromosomes contribute unequally to meiotic success, yet directly testing this prediction has been difficult [7,8] because chromosome-specific pairing is rarely measurable in non-model systems. By combining our chromosome-level assemblies with newly developed chromosome-painting probes, we were able to quantify pairing of four orthologous chromosome pairs (Ch01A, Ch01B, Ch05, Ch20) in hybrid males. These chromosomes showed strikingly different bivalent formation rates (Figure 6D): for instance, Ch01B formed bivalents in about two-thirds of spermatocytes, whereas the Y-linked Ch01A paired in only 2 of >300 cells. Likewise, for Ch05 and Ch20, previous work shows that hybrid males typically form ~5 bivalents per cell [16], meaning that ~25 cells out of the 125 examined would be

expected to contain a given bivalent under random pairing. Instead, we observed far fewer, revealing strong, chromosome-specific biases in pairing success. We also detected interindividual differences in Ch01B pairing between the two hybrid males analysed, suggesting that both chromosome identity and individual background determine pairing propensity.

These results, the first of their kind in an asexually reproducing vertebrate, gain additional importance when viewed in light of how asexual genomes evolve. Asexual hybrids are known to accumulate loss of heterozygosity (LOH) through gene conversion or occasional recombination-like events [20–22,90] and [20] showed that such LOHs are distributed non-randomly across the genome, with biases toward regions of high expression, higher GC content, and specific functional categories. Many hybrid asexuals—including *Cobitis*—rely on PMER, which normally creates identical sister chromatids that can pair without requiring interactions between parental orthologs. Nevertheless, occasional orthologous pairing has been proposed as the mechanistic source of observed LOHs. Our discovery that orthologous chromosomes differ strongly in their ability to form bivalents in hybrid meiosis introduces an important, previously unexplored dimension: if only a subset of chromosomes readily engages in inter-ortholog pairing during PMER, then LOH should preferentially accumulate on these linkage groups, while chromosomes with very low pairing propensity may remain largely unaffected. This provides a new mechanistic hypothesis linking chromosome-specific pairing biases to the long-term genomic architecture of asexual lineages.

Hybridising species have nonhomologous genetic sex determination systems

The currently available Tree Of Sex database v.1 [91] lists 40 cypriniformes species for which sex determination has been investigated. However, the existence of genetic sex determination (GSD) has only been found in four species across the entire Cobitoidei suborder, namely multiple sex chromosomes $X_1X_1X_2X_2/X_1X_2Y$ in *C. striata* [92] and *C. rossomeridianalis* [93], XX/XY_1Y_2 in *Schistura fasciolata* [94] as well as ZZ/ZW in *Lepidocephalichthys guntea* [95]. Additionally, two recent papers [81,82] indicated the presence of sex chromosomes in two Asian loaches, an XX/XY system in *Oreonectes platycephalus* and a ZZ/ZW system in *Paramisgurnus dabryanus*, respectively.

Our study brings robust evidence for the presence of GSD in this group of fish, clearly identifying candidate genomic regions/chromosomes associated with sex in each species and validating these predictions with PCR primers in two of the species, which enables further comparative studies within this dynamically evolving field of research. Our study revealed dynamic sex-chromosome turnover within *Cobitis*, namely weak signal (*C. taenia*) or ambiguous signal (*C. tanaitica*) on two independent linkage groups, while relatively well differentiated XY chromosomes in linkage group Ch01A of *C. elongatoides*, which contains a gene previously associated with GSD in the blue tilapia and four additional genes known to regulate the sex determination cascade in vertebrates (Supplementary Table S6). This suggests a rapid turnover of sex chromosomes within the lineage which diverged less than 10 Mya (divergence of *C. elongatoides* from the other species) or even more recently, if we consider that *C. taenia* and C. *tanaitica* diverged in the last 2 Mya (Janko et al., 2018) and appear to have different sex chromosomes (Ch05 and Ch06 respectively).

The observed turnover of sex chromosomes among closely related *Cobitis* species aligns with the well-documented evolutionary plasticity of GSD systems in teleost fishes [96,97]. Moreover, the sex chromosomes identified in the other cobitoid species (XY in *Oreonectes platycephalus* and ZW in *Paramisgurnus dabryanus*) originated from different syntenic groups (Supplementary Table S15).

However, our finding that *C. elongatoides* utilises a GSD system (XY on Ch01A) that is non-homologous to the putative sex-determining regions in *C. tanaitica* (XY on Ch06) and *C. taenia* (XY on Ch05) adds a significant dimension to understanding the inherent link between interspecific hybridisation and the evolution of asexuality and polyploidy as hybridisation between *C. elongatoides* and the other two species consistently produces sterile males and fertile, clonally reproducing females. Such asymmetric patterns are not unique to *Cobitis* but rather have been consistently reported across diverse vertebrate taxa following hybridisation events and have long suggested a potential role for sex chromosomes in linking hybrid sterility and asexuality [18]. Interestingly, in most studied systems involving asexual hybrids, sex chromosomes remained undifferentiated, unknown, or poorly characterised, leaving this hypothesis largely speculative until now [18]. However, among those asexual systems with known sex chromosomes, the ZZ/ZW prevails over XX/XY [18].

While such asymmetries superficially align with Haldane's Rule, especially given current evidence for male heterogamety in loaches, the fertility of hybrid females is not maintained through typical meiotic repair mechanisms but arises via PMER, allowing bivalents to form between identical chromosomal copies. This bypasses meiotic pairing issues and restores fertility. Crucially, however, PMER is restricted to female hybrids, which subsequently reproduce clonally, while hybrid males remain sterile. Notably, transplantation experiments have demonstrated that PMER can be reactivated in spermatogonial cells of hybrid males if these cells develop in a female gonadal environment and transdifferentiate into oogonia that may subsequently give rise to unreduced oocytes [19]. While such a finding suggests that the ability to initiate asexual reproduction via PMER is primarily dictated by tissue-specific cues from the female gonadal environment, our discovery adds a new layer of complexity to this. It demonstrates that GSD systems in hybrids represent a combination of fundamentally different mechanisms inherited from parental species. Such an interplay between GSD and the female gonadal environment hints at a deeper integration between genetic triggers, epigenetic regulation, and cellular signalling pathways in enabling PMER. It raises a hypothesis that, while the female-specific gonadal environment acts as a permissive factor for PMER, the genetic sex determination system may function as an upstream regulatory mechanism, shaping how these cellular pathways are activated in hybrids. Our findings, therefore, highlight an exciting new research avenue into the genetic and molecular basis of PMER and the broader evolutionary consequences of sex chromosome turnover in hybrid systems.

Conclusions

Our study highlights how integrating chromosome-level genome assemblies, molecular cytogenetics, and meiotic analysis can illuminate the mechanisms underlying hybrid sterility and asexuality. We show that hybridising *Cobitis* species differ in their sex determination systems, accumulate extensive structural variants, and exhibit non-random,

chromosome-specific pairing affinities during male meiosis. Notably, the frequent mispairing of the *C. elongatoides*-originated Y chromosome Ch01A points to structural or regulatory incompatibilities as potential barriers to normal gametogenesis. Together, these findings underscore how genome divergence between parental species may shape reproductive outcomes in hybrids and pave the way for future research into the chromosomal basis of asexuality.

Additional Files

Supplementary Figure S1. The repeat landscape plot illustrates the transposable element (TE) accumulation history for the three *Cobitis* genomes (*C. taenia*, *C. tanaitica*, and *C. elongatoides*), based on Kimura distance-based copy divergence analyses. The sequence divergence (CpG adjusted Kimura substitution level) is shown on the x-axis while the percentage of the genome represented by each TE type is on the y-axis. Transposon type is indicated by the key on the right.

Supplementary Figure S2. Dotplot analysis of genomic homologies between *Cobitis elongatoides*, *C. taenia*, and *C. tanaitica*. Dotplots depict pairwise genomic comparisons between *C. elongatoides* (E), *C. taenia* (T), and *C. tanaitica* (N), illustrating sequence homology and structural variation. Each panel represents a pairwise alignment: (A) *C. elongatoides* vs. *C. taenia*, (B) *C. taenia* vs. *C. tanaitica* and (C) *C. elongatoides* vs. *C. tanaitica*. Diagonal lines indicate regions of synteny, while disruptions or scattered points suggest structural rearrangements such as inversions, translocations, or duplications. The density and continuity of dot patterns reflect the level of sequence similarity and collinearity between species**.**

Supplementary Figure S3. Synteny plots of homologous sequences, intrachromosomal rearrangements, and previously described major satellite sequence arrays in chromosomes of the three *Cobitis* species (from top to bottom *C. tanaitica*, *C. taenia* and *C. elongatoides*). Darker colour shades highlight the SyRI detected events while lighter shades show the results of parsimonious merging. Only blocks longer than 5 Kbp are shown.

Supplementary Figure S4. Chromosome painting of Ch05 (green) and Ch20 (red) (A-C) as well as Ch01A (green) and Ch01B (red) (D-E) on meiotic metaphases of *C. taenia* (A, D), *C. tanaitica* (B, E), *C. elongatoides* (C, F). Ch05 and Ch20 (a-c) indicate two bivalents in all species. Chromosome painting of Ch01A and Ch01B indicates one bivalent in *C. taenia* (D) while two bivalents in *C. tanaitica* (E) and *C. elongatoides* (F). Chromosomes are stained by DAPI (blue). Scale bar = 10 μm.

Supplementary Table S1. List of the specimens used in the study, including information on the species and sex of the individuals, the tissues taken from them, together with the purpose and technique of the study and where the specimens were collected.

Supplementary Table S2. Design of oligo probes for chromosome-specific FISH visualisation.

Supplementary Table S3. Primers used for PCR amplification and the number of tested individuals of *C. elongatoides* and *C. taenia*. A minus sign (-) indicates no detectable signal in electrophoresis after PCR amplification, while a plus sign (+) indicates the presence of a strong band.

Supplementary Table S4. PCR amplification conditions (valid for all primer combinations).

Supplementary Table S5. List of known master sex-determining genes (MSD), candidate MSD, or those connected with male- or female-developing pathway (sex-related) in actinopterygians. Genes missing from any *Cobitis* annotation file are orange marked.

Supplementary Table S6. Distribution of identified master sex-determination genes on chromosomes in three loach species among Actinopterygians. Confirmed master sex-determining genes are marked in bold.

Supplementary Table S7. Pre- and post-Hi-C assembly statistics.

Supplementary Table S8. Superscaffolds lengths (in bp).

Supplementary Table S9. Hi-C mapping quality stats.

Supplementary Table S10. Number of bases and annotated genes per chromosome.

Supplementary Table S11. Compartments and TADs metrics.

Supplementary Table S12. Counts of structural variants as detected by SyRI reported by different types and lengths.

Supplementary Table S13. Counts of intrachromosomal rearrangements after the filtering step leaving out the shortest (< 5 Kbp) variants and the merging step combining variants matching in their type, location and orientation.

Supplementary Table S14. List of all primers tested for PCR amplification and the number of tested individuals of *C. elongatoides* and *C. taenia*. Rows coloured in light green are showing primer pairs which gave expected PCR results on both species.

Supplementary Table S15. Chromosome homologies between cobitoid and a reference cyprinid (*Danio rerio*) species identified in available chromosome level genome assemblies. Sex chromosomes are marked with purple. Fused chromosomes are bold.

List of Abbreviations

Bwa – Burrows-Wheeler Aligner; ET – C. elongatoides-taenia hybrid; GLM – generalised linear model; GSD – genetic sex determination; LINE – long interspersed nuclear element; LTR – long terminal repeat; Mya – million years ago; ONT – Oxford Nanopore Technology; PMER – premeiotic endoreplication; SSC – saline-sodium citrate; SV – structural variant; TAD – topologically associated domain; TE – transposable element.

Ethics Approval

The Valid Animal Use Protocol was in force during the study at the Institute of Animal Physiology and Genetics, Liběchov, Czech Republic (No. CZ 02386). All institutional and national guidelines were covered by the "Valid Animal Use Protocol" No. CZ 02386 of the Laboratory of Fish genetics.

Acknowledgements

Authors are profoundly obliged to our greatest technicians, Š. Pelikanová, J. Machová and P. Šejnohová. We thank M. Schartl and M. Stöck for inspiring advice regarding the PoolSeq analysis and sex chromosome identification. Part of this work was carried out with the support of ELIXIR CZ Research Infrastructure (ID LM2023055, MEYS CR).

Author Contributions

K.J. and J.P. conceived and supervised the research. D.D., L.A., T.T., D.K., J.K., A.M., K.J., P.P., P.H. collected the samples and performed the experiments. S.A.S., D.D., V.T., Y.G., K.J., Z.H., M.K., A.R.-H., L.Á.-G., G.P., E.H., L.A., O.B., T.T., M.K.D., A.B., H.K. analysed data. S.A.S., D.D., V.T., K.J., J.P., Z.H., L.Á.-G., L.A., T.T., A.B., R.R., Y.G. interpreted the data and wrote the manuscript. All authors have read and approved the final manuscript.

Funding

The study was supported by the Czech Science Foundation Projects No. 21-25185S and No. 24-12217S. Institute of Animal Physiology and Genetics receives support from Institutional Research Concept, Grant/ Award Number: RVO67985904. S.A.S. was supported by the Charles University Research Centre program No. UNCE/24/SCI/006. V.T. was supported by the Marie Skłodowska-Curie Actions - COFUND project, which is co-funded by the European Union (MERIT - Grant Agreement No. 101081195). A.R.-H. is founded by the Spanish Ministry of Science and Innovation (PID2020-112557GB-I00 funded by AEI/10.13039/501100011033), the Agència de Gestió d’Ajuts Universitaris i de Recerca, AGAUR (2021SGR00122) and the Catalan Institution for Research and Advanced Studies (ICREA). L.A.-G. and G.P. were supported by FPI predoctoral fellowships from the Ministry of Economy, Industry, and Competitiveness (PRE-2018-083257 and PRE-C-2021-0083, respectively). Z.H. was supported by the Grant Agency of Charles University (grant number 314222) and SVV 260818/2025.

Data Availability

The raw genomic sequencing data for all analysed species have been deposited in the European Nucleotide Archive (ENA) under BioProject accession number PRJEB90107. Previously published transcriptomic data included RNAseq reported by Bartoš et al. (PRJEB92117)[23] and Janko et al. (PRJNA630963)[25]. All additional supporting data are available in the GigaDB [98].

Competing Interests

The authors declare that they have no competing interests.

Declaration of Generative AI and AI-Assisted Technologies in the Writing Process

During the preparation of this manuscript, the authors used ChatGPT to improve language style and correct grammatical errors. Following its use, the authors carefully reviewed and edited the content as necessary and took full responsibility for the final version of the publication.

References

1. Kochakpour N, Moens PB. Sex-specific crossover patterns in zebrafish (*Danio rerio*). *Heredity*. 2008. Doi: 10.1038/sj.hdy.6801091.
2. Lenormand T, Engelstädter J, Johnston SE, Wijnker E, Haag CR. Evolutionary mysteries in meiosis. *Philos Trans R Soc Lond B Biol Sci*. 2016. Doi: 10.1098/rstb.2016.0001.
3. Ortiz-Barrientos D, Engelstädter J, Rieseberg LH. Recombination rate evolution and the origin of species. *Trends Ecol Evol*. 2016. Doi: 10.1016/j.tree.2015.12.016.
4. Thompson MJ, Jiggins CD. Supergenes and their role in evolution. *Heredity*. 2014. Doi: 10.1038/hdy.2014.20.
5. Berdan EL, Aubier TG, Cozzolino S, Faria R, Feder JL, Giménez MD, et al. Structural variants and speciation: multiple processes at play. *Cold Spring Harb Perspect Biol*. 2024. Doi: 10.1101/cshperspect.a041446.
6. Zhang L, Reifová R, Halenková Z, Gompert Z. How important are structural variants for speciation? *Genes*. 2021. Doi: 10.3390/genes12071084.
7. Bhattacharyya T, Gregorova S, Mihola O, Anger M, Sebestova J, Denny P, et al. Mechanistic basis of infertility of mouse intersubspecific hybrids. *Proc Natl Acad Sci U S A*. 2013. Doi: 10.1073/pnas.1219126110.
8. Forejt J, Jansa P. Meiotic recognition of evolutionarily diverged homologs: chromosomal hybrid sterility revisited. *Mol Biol Evol*. 2023. Doi: 10.1093/molbev/msad083.
9. Gregorova S, Gergelits V, Chvatalova I, Bhattacharyya T, Valiskova B, Fotopulosova V, et al. Modulation of PRDM9-controlled meiotic chromosome asynapsis overrides hybrid sterility in mice. *eLife*. 2018. Doi: 10.7554/eLife.34282.
10. Janko K, Mikulíček P, Hobza R, Schlupp I. Sperm-dependent asexual species and their role in ecology and evolution. *Ecol Evol*. 2023. Doi: 10.1002/ece3.10522.

11. Stenberg P, Saura A. Cytology of asexual animals. Lost sex: the evolutionary biology of parthenogenesis. *Dordr*. 2009. Doi: 10.1007/978-90-481-2770-2_4.
12. Stenberg P, Saura A. Meiosis and its deviations in polyploid animals. *Cytogenet Genome Res*. 2013. Doi: 10.1159/000351731.
13. Moritz C, Brown WM, Densmore LD, Wright JW, Vyas D, Donnellan S, et al. Genetic diversity and the dynamics of hybrid parthenogenesis in *Cnemidophorus* (Teiidae) and *Heteronotia* (Gekkonidae). Evolution and ecology of unisexual vertebrates. Albany, NY: New York State Museum; 1989. p. 87–112.
14. Marta A, Tichopád T, Bartoš O, Klíma J, Shah MA, Bohlen VŠ, et al. Genetic and karyotype divergence between parents affect clonality and sterility in hybrids. *eLife*. 2023. Doi: 10.7554/eLife.88366.
15. Arai K, Fujimoto T. Genomic constitution and atypical reproduction in polyploid and unisexual lineages of the *Misgurnus* loach, a teleost fish. *Cytogenet Genome Res*. 2013. Doi: 10.1159/000353301.
16. Dedukh D, Majtánová Z, Marta A, Pšenička M, Kotusz J, Klíma J, et al. Parthenogenesis as a solution to hybrid sterility: the mechanistic basis of meiotic distortions in clonal and sterile hybrids. *Genetics*. 2020. Doi: 10.1534/genetics.119.302988.
17. Lutes AA, Neaves WB, Baumann DP, Wiegraebe W, Baumann P. Sister chromosome pairing maintains heterozygosity in parthenogenetic lizards. *Nature*. 2010. Doi: 10.1038/nature08818.
18. Stöck M, Dedukh D, Reifová R, Lamatsch DK, Starostová Z, Janko K. Sex chromosomes in meiotic, hemiclonal, clonal and polyploid hybrid vertebrates: along the 'extended speciation continuum'. *Philos Trans R Soc Lond B Biol Sci*. 2021. Doi: 10.1098/rstb.2020.0103.
19. Tichopád T, Franěk R, Doležálková-Kaštánková M, Dedukh D, Marta A, Halačka K, et al. Clonal gametogenesis is triggered by intrinsic stimuli in the hybrid's germ cells but is dependent on sex differentiation. *Biol Reprod*. 2022. Doi: 10.1093/biolre/ioac074.
20. Janko K, Bartoš O, Kočí J, Roslein J, Drdová EJ, Kotusz J, et al. Genome fractionation and loss of heterozygosity in hybrids and polyploids: mechanisms, consequences for selection, and link to gene function. *Mol Biol Evol*. 2021. Doi: 10.1093/molbev/msab249.
21. Jaron KS, Bast J, Nowell RW, Ranallo-Benavidez TR, Robinson-Rechavi M, Schwander T. Genomic features of parthenogenetic animals. *J Hered*. 2021. Doi: 10.1093/jhered/esaa031.
22. Warren WC, García-Pérez R, Xu S, Lampert KP, Chalopin D, Stöck M, et al. Clonal polymorphism and high heterozygosity in the celibate genome of the Amazon molly. *Nat Ecol Evol*. 2018. Doi: 10.1038/s41559-018-0473-y.
23. Bartoš O, Röslein J, Kotusz J, Paces J, Pekárik L, Petrtýl M, et al. The legacy of sexual ancestors in phenotypic variability, gene expression, and homoeolog regulation of asexual hybrids and polyploids. *Mol Biol Evol*. 2019. Doi: 10.1093/molbev/msz114.
24. Albertini E, Barcaccia G, Carman JG, Pupilli F. Did apomixis evolve from sex or was it the other way around? *J Exp Bot*. 2019. Doi: 10.1093/jxb/erz109.
25. Janko K, Pačes J, Wilkinson-Herbots H, Costa RJ, Roslein J, Drozd P, et al. Hybrid asexuality as a primary postzygotic barrier between nascent species: on the interconnection between asexuality, hybridization and speciation. *Mol Ecol*. 2018. Doi: 10.1111/mec.14377.
26. Murphy RW, Fu J, Macculloch RD, Darevsky IS, Kupriyanova LA. A fine line between sex and unisexuality: the phylogenetic constraints on parthenogenesis in lacertid lizards. *Zool J Linn Soc*. 2000. Doi: 10.1111/j.1096-3642.2000.tb02200.x.

27. Janko K, Kotusz J, De Gelas K, Šlechtová V, Opoldusová Z, Drozd P, et al. Dynamic formation of asexual diploid and polyploid lineages: multilocus analysis of *Cobitis* reveals the mechanisms maintaining the diversity of clones. *PLoS One*. 2012. Doi: 10.1371/journal.pone.0045384.
28. Majtánová Z, Choleva L, Symonová R, Ráb P, Kotusz J, Pekárik L, et al. Asexual reproduction does not apparently increase the rate of chromosomal evolution: karyotype stability in diploid and triploid clonal hybrid fish (*Cobitis*, Cypriniformes, Teleostei). *PLoS One*. 2016. Doi: 10.1371/journal.pone.0146872.
29. Janko K, Eisner J, Cigler P, Tichopád T. Unifying framework explaining how parental regulatory divergence can drive gene expression in hybrids and allopolyploids. *Nat Commun*. 2024. Doi: 10.1038/s41467-024-52546-5.
30. Sambrook J, Russell DW. Purification of nucleic acids by extraction with phenol:chloroform. *Cold Spring Harb Protoc*. 2006. Doi: 10.1101/pdb.prot4455.
31. Janko K, Flajšhans M, Choleva L, Bohlen J, Šlechtová V, Rábová M, et al. Diversity of European spined loaches (genus *Cobitis* L.): an update of the geographic distribution of the *Cobitis taenia* hybrid complex with a description of new molecular tools for species and hybrid determination. *J Fish Biol*. 2007. Doi: 10.1111/j.1095-8649.2007.01663.x.
32. Bolger AM, Lohse M, Usadel B. Trimmomatic: a flexible trimmer for Illumina sequence data. *Bioinformatics*. 2014; 30:2114–20. Doi: 10.1093/bioinformatics/btu170.
33. Rio DC, Ares M, Hannon GJ, Nilsen TW. Purification of RNA using TRIzol (TRI Reagent). *Cold Spring Harb Protoc*. 2010. Doi: 10.1101/pdb.prot5439.
34. Jackman SD, Vandervalk BP, Mohamadi H, Chu J, Yeo S, Hammond SA, et al. ABySS 2.0: resource-efficient assembly of large genomes using a Bloom filter. *Genome Res*. 2017. Doi: 10.1101/gr.214346.116.
35. Li D, Liu C-M, Luo R, Sadakane K, Lam T-W. MEGAHIT: an ultra-fast single-node solution for large and complex metagenomics assembly via succinct de Bruijn graph. *Bioinformatics*. 2015. Doi: 10.1093/bioinformatics/btv033.
36. Kolmogorov M, Yuan J, Lin Y, Pevzner PA. Assembly of long, error-prone reads using repeat graphs. *Nat Biotechnol*. 2019. Doi: 10.1038/s41587-019-0072-8.
37. Walker BJ, Abeel T, Shea T, Priest M, Abouelliel A, Sakthikumar S, et al. Pilon: an integrated tool for comprehensive microbial variant detection and genome assembly improvement. *PLoS One*. 2014. Doi: 10.1371/journal.pone.0112963.
38. Li H. Aligning sequence reads, clone sequences and assembly contigs with BWA-MEM. arXiv. 2013. Doi: 10.48550/arXiv.1303.3997.
39. Danecek P, Bonfield JK, Liddle J, Marshall J, Ohan V, Pollard MO, et al. Twelve years of SAMtools and BCFtools. *GigaScience*. 2021. Doi: 10.1093/gigascience/giab008.
40. Dudchenko O, Batra SS, Omer AD, Nyquist SK, Hoeger M, Durand NC, et al. De novo assembly of the *Aedes aegypti* genome using Hi-C yields chromosome-length scaffolds. *Science*. 2017. Doi: 10.1126/science.aal3327.
41. Bushnell B. BBMap: a fast, accurate, splice-aware aligner. 2014. Technical report. Lawrence Berkeley National Laboratory, Berkeley, CA, USA.
42. Wolff J, Rabbani L, Gilsbach R, Richard G, Manke T, Backofen R, et al. Galaxy HiCExplorer 3: a web server for reproducible Hi-C, capture Hi-C and single-cell Hi-C data analysis, quality control and visualization. *Nucleic Acids Res*. 2020. Doi: 10.1093/nar/gkaa220.
43. Kruse K, Hug CB, Vaquerizas JM. FAN-C: a feature-rich framework for the analysis and visualisation of chromosome conformation capture data. *Genome Biol*. 2020. Doi: 10.1186/s13059-020-02215-9.

44. Álvarez-González L, Burden F, Doddamani D, Malinverni R, Leach E, Marín-García C, et al. 3D chromatin remodelling in the germ line modulates genome evolutionary plasticity. *Nat Commun*. 2022. Doi: 10.1038/s41467-022-30296-6.
45. Flynn JM, Hubley R, Goubert C, Rosen J, Clark AG, Feschotte C, et al. RepeatModeler2 for automated genomic discovery of transposable element families. *Proc Natl Acad Sci U S A*. 2020. Doi: 10.1073/pnas.1921046117.
46. Bao Z, Eddy SR. Automated de novo identification of repeat sequence families in sequenced genomes. *Genome Res*. 2002. Doi: 10.1101/gr.88502.
47. Price AL, Jones NC, Pevzner PA. De novo identification of repeat families in large genomes. *Bioinformatics*. 2005. Doi: 10.1093/bioinformatics/bti1018.
48. Smit A, Hubley R, Green P. RepeatMasker Open-4.0. 2013–2015. [Computer software]. http://www.repeatmasker.org.
49. Stanke M, Morgenstern B. AUGUSTUS: a web server for gene prediction in eukaryotes that allows user-defined constraints. *Nucleic Acids Res*. 2005. Doi: 10.1093/nar/gki458.
50. Korf I. Gene finding in novel genomes. *BMC Bioinformatics*. 2004. Doi: 10.1186/1471-2105-5-59.
51. Dobin A, Davis CA, Schlesinger F, Drenkow J, Zaleski C, Jha S, et al. STAR: ultrafast universal RNA-seq aligner. *Bioinformatics*. 2013. Doi: 10.1093/bioinformatics/bts635.
52. Keller O, Kollmar M, Stanke M, Waack S. A novel hybrid gene prediction method employing protein multiple sequence alignments. *Bioinformatics*. 2011. Doi: 10.1093/bioinformatics/btr010.
53. Stanke M, Keller O, Gunduz I, Hayes A, Waack S, Morgenstern B. AUGUSTUS: ab initio prediction of alternative transcripts. *Nucleic Acids Res*. 2006. Doi: 10.1093/nar/gkl200.
54. Stanke M, Diekhans M, Baertsch R, Haussler D. Using native and syntenically mapped cDNA alignments to improve de novo gene finding. *Bioinformatics*. 2008. Doi: 10.1093/bioinformatics/btn013.
55. Campbell MS, Holt C, Moore B, Yandell M. Genome annotation and curation using MAKER and MAKER-P. *Curr Protoc Bioinformatics*. 2014. Doi: 10.1002/0471250953.bi0411s48.
56. The UniProt Consortium, Bateman A, Martin M-J, Orchard S, Magrane M, Adesina A, et al. UniProt: the universal protein knowledgebase in 2025. *Nucleic Acids Res*. 2025. Doi: 10.1093/nar/gkae1010.
57. Altschul SF, Gish W, Miller W, Myers EW, Lipman DJ. Basic local alignment search tool. *J Mol Biol*. 1990. Doi: 10.1016/S0022-2836(05)80360-2.
58. Chan PP, Lin BY, Mak AJ, Lowe TM. tRNAscan-SE 2.0: improved detection and functional classification of transfer RNA genes. *Nucleic Acids Res*. 2021. Doi: 10.1093/nar/gkab688.
59. Shumate A, Wong B, Pertea G, Pertea M. Improved transcriptome assembly using a hybrid of long and short reads with StringTie. *PLoS Comput Biol*. 2022. Doi: 10.1371/journal.pcbi.1009730.
60. Haas B. TransDecoder (version v5.7.1). 2023. [Computer software]. https://github.com/TransDecoder/TransDecoder.
61. Dainat J. NBISweden/AGAT: AGAT v1.4.1. 2024. [Computer software]. Zenodo. Doi: 10.5281/zenodo.13799920.
62. Kuhl H. HANNO: efficient high-throughput annotation of protein-coding genes in eukaryote genomes (version v0.4). 2024. [Computer software]. Zenodo. Doi: 10.5281/zenodo.11532370.
63. Kim D, Paggi JM, Park C, Bennett C, Salzberg SL. Graph-based genome alignment and genotyping with HISAT2 and HISAT-genotype. *Nat Biotechnol*. 2019. Doi: 10.1038/s41587-019-0201-4.

64. Storer J, Hubley R, Rosen J, Wheeler TJ, Smit AF. The Dfam community resource of transposable element families, sequence models, and genome annotations. *Mob DNA*. 2021. Doi: 10.1186/s13100-020-00230-y.
65. Li H. Minimap2: pairwise alignment for nucleotide sequences. *Bioinformatics*. 2018. Doi: 10.1093/bioinformatics/bty191.
66. Cabanettes F, Klopp C. D-GENIES: dot plot large genomes in an interactive, efficient and simple way. *PeerJ*. 2018. Doi: 10.7717/peerj.4958.
67. Goel M, Sun H, Jiao W-B, Schneeberger K. SyRI: finding genomic rearrangements and local sequence differences from whole-genome assemblies. *Genome Biol*. 2019. Doi: 10.1186/s13059-019-1911-0.
68. He W, Yang J, Jing Y, Xu L, Yu K, Fang X. NGenomeSyn: an easy-to-use and flexible tool for publication-ready visualization of syntenic relationships across multiple genomes. *Bioinformatics*. 2023. Doi: 10.1093/bioinformatics/btad121.
69. Tang H, Krishnakumar V, Zeng X, Xu Z, Taranto A, Lomas JS, et al. JCVI: a versatile toolkit for comparative genomics analysis. *iMeta*. 2024. Doi: 10.1002/imt2.211.
70. Quinlan AR. BEDTools: the Swiss-army tool for genome feature analysis. *Curr Protoc Bioinformatics*. 2014. Doi: 10.1002/0471250953.bi1112s47.
71. Marta A, Dedukh D, Bartoš O, Majtánová Z, Janko K. Cytogenetic characterization of seven novel satDNA markers in two species of spined loaches (*Cobitis*) and their clonal hybrids. *Genes (Basel)*. 2020. Doi: 10.3390/genes11060617.
72. Zhang T. Chorus2 (version v2.0.3). 2018. [Computer software]. https://github.com/zhangtaolab/Chorus2.
73. McKenna A, Hanna M, Banks E, Sivachenko A, Cibulskis K, Kernytsky A, et al. The genome analysis toolkit: a MapReduce framework for analyzing next-generation DNA sequencing data. *Genome Res*. 2010. Doi: 10.1101/gr.107524.110.
74. Chow S, Hazama K. Universal PCR primers for S7 ribosomal protein gene introns in fish. *Mol Ecol*. 1998. Doi: 10.1046/j.1365-294x.1998.00425.x.
75. Pérez-Rico YA, Barillot E, Shkumatava A. Demarcation of topologically associating domains is uncoupled from enriched CTCF binding in developing zebrafish. *iScience*. 2020. Doi: 10.1016/j.isci.2020.101046.
76. Perdices A, Bohlen J, Šlechtová V, Doadrio I. Molecular evidence for multiple origins of the European spined loaches (Teleostei, Cobitidae). *PLoS One*. 2016. Doi: 10.1371/journal.pone.0144628.
77. Kim J, Lee C, Ko BJ, Yoo DA, Won S, Phillippy AM, et al. False gene and chromosome losses in genome assemblies caused by GC content variation and repeats. *Genome Biol*. 2022. Doi: 10.1186/s13059-022-02765-0.
78. Vara C, Paytuví-Gallart A, Cuartero Y, Álvarez-González L, Marín-Gual L, Garcia F, et al. The impact of chromosomal fusions on 3D genome folding and recombination in the germ line. *Nat Commun*. 2021. Doi: 10.1038/s41467-021-23270-1.
79. Vasil'ev VP, Vasil'eva KD, Osinov AG. Evolution of a diploid–triploid–tetraploid complex in fishes of the genus *Cobitis* (Pisces, Cobitidae). Evolution and ecology of unisexual vertebrates. Albany, NY: University of the State of New York, State Education Department, New York State Museum; 1989. p. 153–69.
80. Ráb P, Rábová M, Bohlen J, Lusk S. Genetic differentiation of the two hybrid diploid–polyploid complexes of loaches, genus *Cobitis* (Cobitidae) involving *C. taenia*, *C. elongatoides* and *C. spp.* in the Czech Republic: karyotypes and cytogenetic diversity. *Folia Zool*. 2000; 49:55–66.

81. Zhang L, Zhang W, Cheng Y, Fang Y, Guan X, Gong A, et al. Chromosome-level genome assembly and annotation of the gynogenetic large-scale loach (*Paramisgurnus dabryanus*). *Sci Data*. 2025. Doi: 10.1038/s41597-025-04498-8.
82. Wang X, Wang D, Wang H, Dudgeon D, Reid K, Merilä J. Chromosome-level haplotype-resolved genome of the tropical loach (*Oreonectes platycephalus*). *Sci Data*. 2025. Doi: 10.1038/s41597-024-04301-0.
83. Ho V, Massey TE, King WD. Thymidylate synthase gene polymorphisms and markers of DNA methylation capacity. *Mol Genet Metab*. 2011. Doi: 10.1016/j.ymgme.2010.12.015.
84. Stevison LS, Hoehn KB, Noor MAF. Effects of inversions on within- and between-species recombination and divergence. *Genome Biol Evol*. 2011. Doi: 10.1093/gbe/evr081.
85. Hooper DM, Price TD. Chromosomal inversion differences correlate with range overlap in passerine birds. *Nat Ecol Evol*. 2017. Doi: 10.1038/s41559-017-0284-6.
86. He C, Zhang X, Wen Z, Shi Q, Song Z. A chromosome-scale reference genome assembly for *Triplophysa lixianensis*. *Sci Data*. 2024. Doi: 10.1038/s41597-024-04268-y.
87. Deng Y, Meng M, Fang J, Jiang H, Sun N, Lv W, et al. Genome of the butterfly hillstream loach provides insights into adaptations to torrential mountain stream life. *Mol Ecol Resour*. 2021. Doi: 10.1111/1755-0998.13400.
88. Shao F, Han M, Peng Z. Evolution and diversity of transposable elements in fish genomes. *Sci Rep*. 2019. Doi: 10.1038/s41598-019-51888-1.
89. Sotero-Caio CG, Platt RN, Suh A, Ray DA. Evolution and diversity of transposable elements in vertebrate genomes. *Genome Biol Evol*. 2017. Doi: 10.1093/gbe/evw264.
90. Tucker AE, Ackerman MS, Eads BD, Xu S, Lynch M. Population-genomic insights into the evolutionary origin and fate of obligately asexual *Daphnia pulex*. *Proc Natl Acad Sci U S A*. 2013. Doi: 10.1073/pnas.1313388110.
91. Jeffries D, Benvenuto C, Böhne A, Fraisse C, Garcia S, Jay P, et al. The Tree of Sex consortium: a global initiative for studying the evolution of reproduction in eukaryotes. *J Evol Biol*. 2025. Doi: 10.1093/jeb/voaf053.
92. Saitoh K. Multiple sex-chromosome system in a loach fish. *Cytogenet Genome Res*. 1989. Doi: 10.1159/000132840.
93. Vasil'eva ED, Vasil'ev VP. Sibling species in genus *Cobitis* (Cobitidae). *Cobitis rossomeridionalis sp. nova*. *J Ichthyol*. 1998; 38:580–90.
94. Sember A, Bohlen J, Šlechtová V, Altmanová M, Symonová R, Ráb P. Karyotype differentiation in 19 species of river loach fishes (Nemacheilidae, Teleostei): extensive variability associated with rDNA and heterochromatin distribution and its phylogenetic and ecological interpretation. *BMC Evol Biol*. 2015. Doi: 10.1186/s12862-015-0532-9.
95. Sharma OP, Tripathi NK. Female heterogamety in two teleostean fishes. *Cytologia*. 1988. Doi: 10.1508/cytologia.53.81.
96. Heule C, Salzburger W, Böhne A. Genetics of sexual development: an evolutionary playground for fish. *Genetics*. 2014. Doi: 10.1534/genetics.114.161158.
97. Mank JE, Avise JC. Evolutionary diversity and turnover of sex determination in teleost fishes. *Sex Dev*. 2009. Doi: 10.1159/000223071.
98. Schlebusch S A;Trifonov V;Halenková Z;Klianitskaya M;Dedukh D;Ruiz-Herrera A;Álvarez-González L;Pujol G;Hřibová E;Andjel L;Bartoš O;Pajer P;Tichopád T;Kulik D;Kotusz J;Doležálková M K;Böhne A;Marta A;Horna P;Reifová R;Guiguen Y;Kuhl H;Pačes J;Janko K.(2026):Supporting data for "Sex Chromosome Turnover and Structural Genome Divergence Shapes Meiotic Outcomes in Hybridising *Cobitis*".GigaScience database.https://doi.org/10.5524/102814

Figure captions

Figure 1. Map of European rivers indicating the distribution ranges of the three *Cobitis* species included in this study. *C. taenia* is in blue, *C. tanaitica* is in green and *C. elongatoides* is in yellow. Pie charts indicate the sample size and sex ratio of samples taken from each locality (males are indicated by the darker colour and females by the lighter one). The insert indicates a *C. taenia* female individual.

Figure 2. *Cobitis* genomes higher-order chromatin organization. (A) Genome-wide Hi-C contact maps. Contact maps represent 500 Kbp resolution Hi-C matrices obtained using the final assembly as a reference. For the three species clear interacting blocks corresponding to the expected number of chromosomes can be observed. (B) Boxplot depicting the 1st eigenvector distribution of the three species. Eigenvector values are used as a proxy to determine open (A compartments) and close (B compartments) chromatin regions. The similarities in the distribution between the three species indicate similar 3D organization (two-sided t test, ns $p > 0.05$). (C) Boxplot showing insulator score distribution on the three species. Insulator capacity is used to determine TADs strength. Like eigenvector distribution, similarities on the insulator score reflect the same patterns of chromatin folding in the three species (two-sided t test, ns $p > 0.05$). (D) Region-specific 500 Kbp heatmaps, 1st eigenvector and insulator score tracks in the three species. Similar tendencies can be clearly observed.

Figure 3. Repeatome in *Cobitis*. A comparison of relative distribution of individual TE families for each genome (coloured by species). Black bars show the absolute lengths of each family in *C. taenia*.

Figure 4. Syntenic and rearranged regions in *Cobitis* species. A) Gene synteny plot of three *Cobitis* species and *Paramisgurnus dabryanus* (from left to right *Cobitis tanaitica*, *C. taenia*, *C. elongatoides* and *P. dabryanus*). B) Synteny plots of homologous sequences and intrachromosomal rearrangements in four selected chromosomes (chromosome 1, chromosome 4, chromosome 5, chromosome 20) from the three species (from top to bottom *C. tanaitica, C. taenia,* and *C. elongatoides*). Darker colour shades highlight the SyRI detected events while lighter shades show the results of parsimonious merging. Only blocks longer than 5 Kbp are shown.

Figure 5. Chromosome painting of selected chromosomes. Ch01A (red) and Ch01B (green) (A-D) and Ch05 (red) and Ch20 (green) (E-H) are shown on mitotic metaphases of *C. taenia* (A, E), *C. tanaitica* (B, F), *C. elongatoides* (C, G), and diploid ET hybrid (D, H) males. Ch01A and Ch01B are located on different arms of the largest metacentric chromosome in *C. taenia* (A). In *C. tanaitica,* signals appeared on two pairs of subtelocentric chromosomes (B) and in *C. elongatoides*, they were located on two pairs of submetacentric chromosomes (C). Small submetacentric chromosome stained by Ch01A represents the sex chromosome of *C. elongatoides* (C). In diploid ET hybrid, both signals were detected on one metacentric chromosome of *C. taenia* (pointed by arrow) and two submetacentric chromosomes of *C. elongatoides* (D). Chromosome painting of Ch05 showed signals on the long arm of a large submetacentric chromosome across all species (E-G) and in diploid hybrid (H). Chromosome painting of Ch20 was detected in the q-arm of a subtelocentric chromosome in *C. taenia* (E) and *C. elongatoides* (G) and corresponding chromosomes in the diploid hybrid (H) but locates

in a q-arm of a subtelocentric chromosome in *C. tanaitica* (F). Chromosomes are stained by DAPI (blue). Scale bar = 10 µm.

Figure 6. Chromosome pairing in hybrid males. (A-C) Chromosome painting of Ch05 (green) and Ch20 (red) (A) as well as Ch01A (green) and Ch01B (red) (B, C) on meiotic metaphases of diploid hybrid males. Probes for Ch01A and Ch01B hybridised to the *C. taenia* chromosome and two small chromosomes of *C. elongatoides* (B, C). Chr01A of *C. elongatoides* usually existed as univalent (B, C). In some spermatocytes, *C. elongatoides* chromosome hybridising with probe for Chr01B showed pairing with homologous arm of Chr01B of *C. taenia* (B), while in other spermatocytes, there was no pairing between part of the Chr01B of *C. taenia* and chromosome Chr01B of *C. elongatoides* (C). Chromosomes are stained by DAPI (blue). Scale bar = 10 µm. (D) Mosaic plot showing pairing success of four chromosomes (Ch01A, Ch01B, Ch05, Ch20) in two diploid hybrid males. Each chromosome is represented by two adjacent bars (one per individual), with bar height normalised to 100% (brown bars represent the individual one and blue ones the individual two). The lower shaded section indicates the proportion of cells where the chromosome formed a bivalent, while the upper section represents univalents. Numbers within each section show absolute counts. Bar width reflects the total number of spermatocytes analysed per chromosome and individual. Ch01B exhibited the highest pairing frequency, whereas Ch05, Ch20, and especially Ch01A were mostly unpaired.

Figure 7. Signals of sex-linked differentiation across the three *Cobitis* genomes. Differences in depth between male and female individuals are shown on the top row (A-C), with values representing the log2 transformed depth in females divided by the depth in males. The concentrations of sex-specific SNPs are shown on the bottom row (D-F) with values normalised by the number of SNPs identified in that region. Each data point represents the total across a window of 200 Kbp of the genome, with consecutive windows starting 50 Kbp apart. *C. taenia* (A, D) and *C. elongatoides* (C, F) are created using pooled DNA from males and females while *C. tanaitica* (B, E) is created from 3 male and 3 female individuals. Arrows point to the most promising (if any) differentiated sex regions.

Table captions

Table 1. Final genome assembly statistics for *Cobitis taenia, C. tanaitica*, and *C. elongatoides.*

 Click here to access/download;Table;Table_1.xlsx

Table 1. Final genome assembly statistics for the three *Cobitis* species.

**Species**	***C. taenia***	***C. tanaitica***	***C. elongatoides***
Number of Contigs	7.2k	25.8k	28.9k
Cumulative Length (Gbp)	1.63	1.71	1.82
Largest Contig (Mbp)	99.6	86.8	78.1
N50 (Mbp)	53.2	51.3	43
N90 (Kbp)	206	25.7	21.3
L50	13	14	17
L90	238	2365	6549
Number of Chromosomes	24	25	25
Proportion of genome assembled into chromosomes	85.8%	82.2%	68.5%
GC content	40.1%	40.1%	40.5%
Complete BUSCO genes	94.6%	90.9%	88.6%
Fragmented BUSCO genes	1.8%	4.1%	5.2%
Missing BUSCO genes	3.6%	5.0%	6.2%

Figure 1

Click here to access/download;Figure;Figure_1.tiff

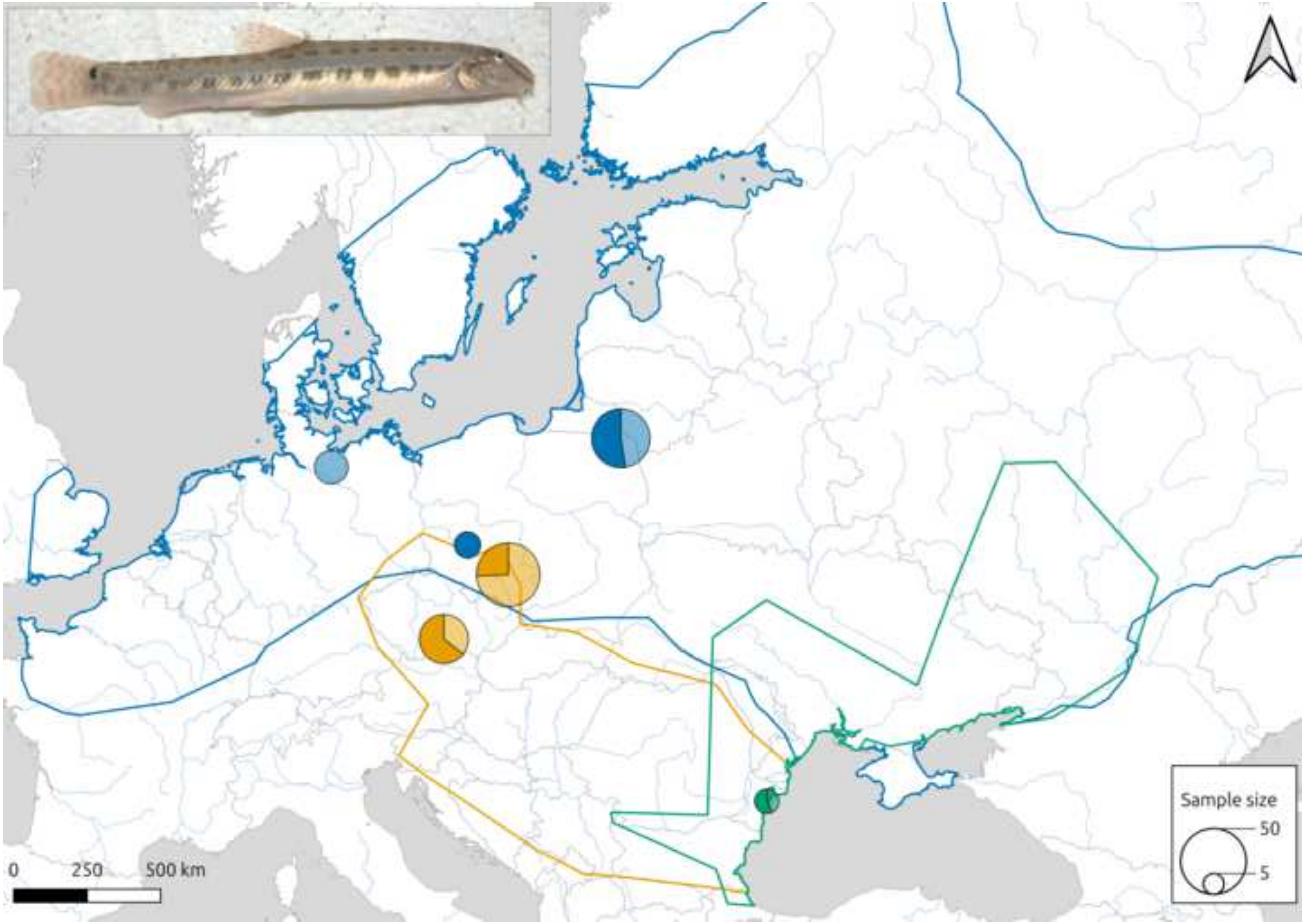

Figure 2

Click here to access/download;Figure;Figure_2.tiff

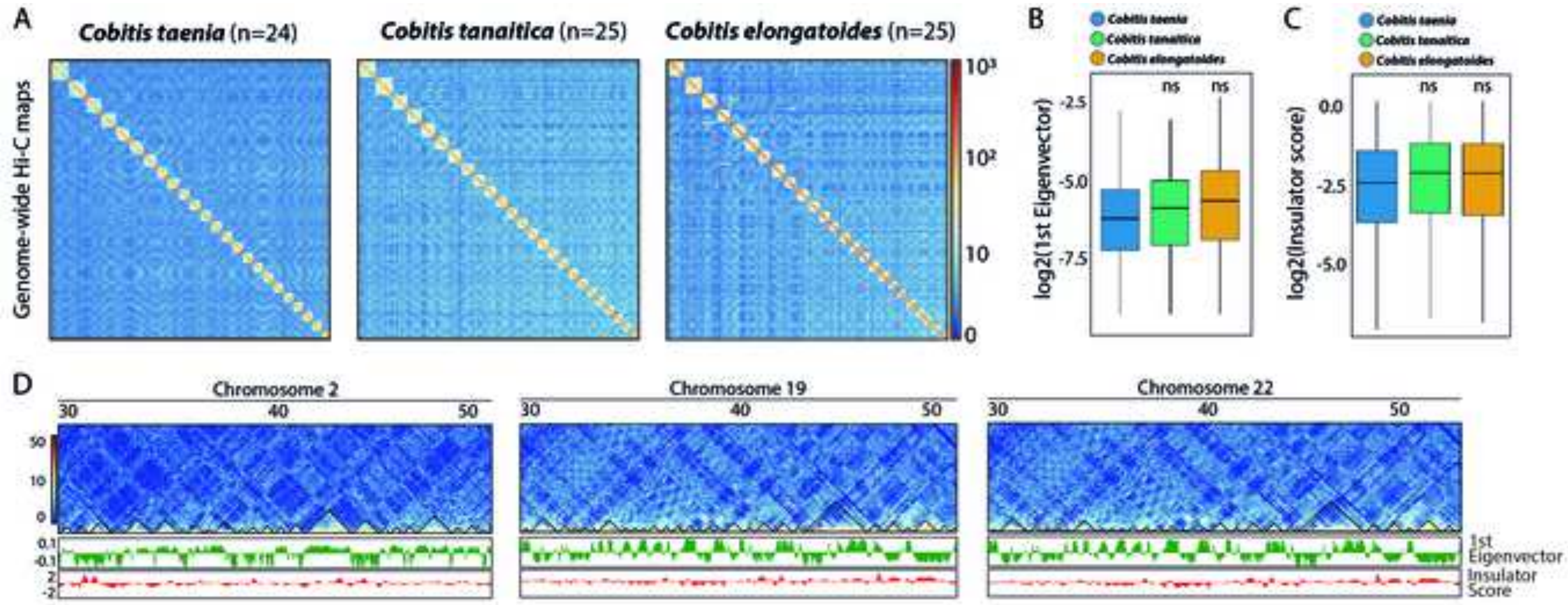

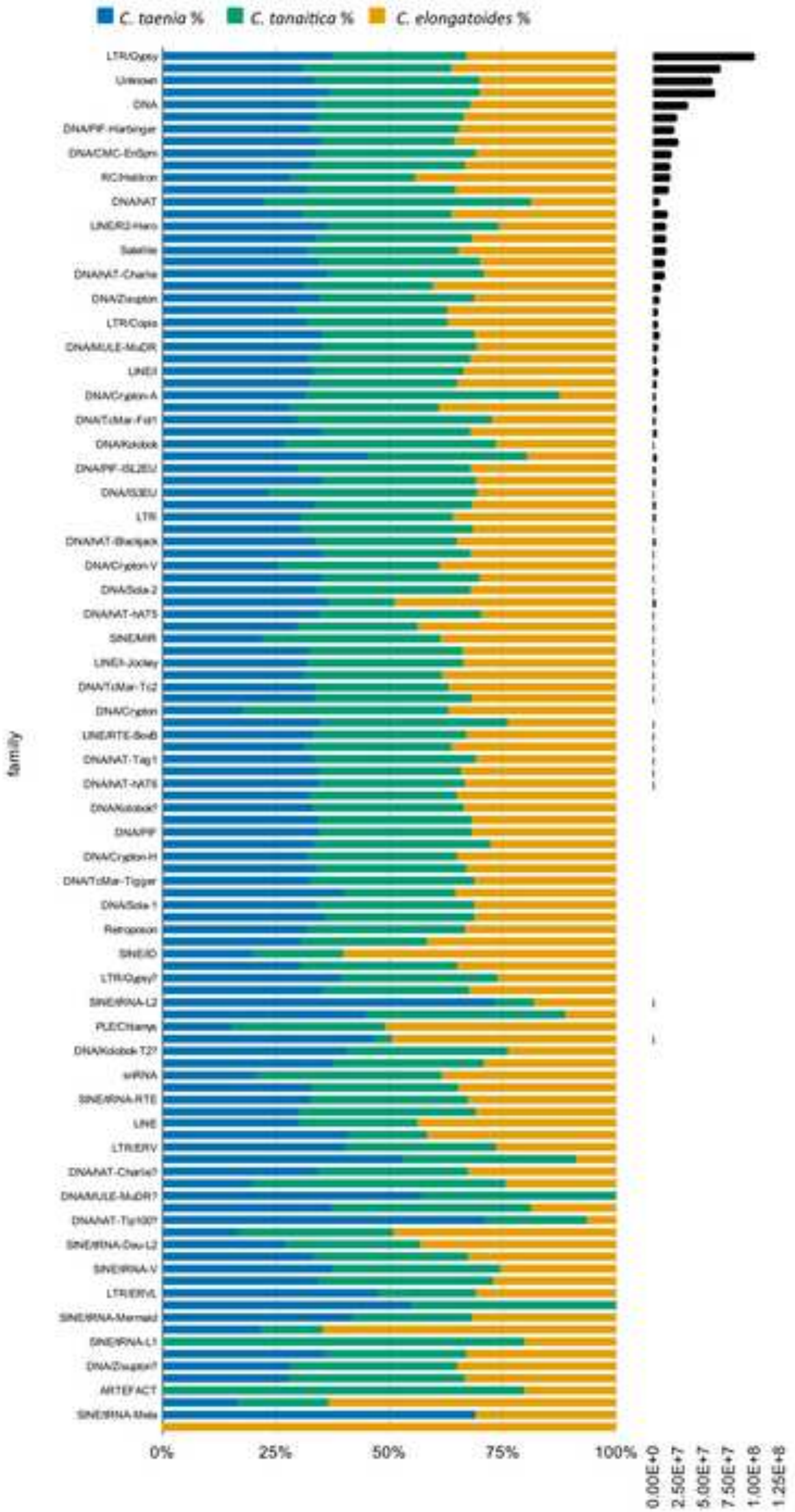
C. taenia %
C. tanaitica %
C. elongatoides %
family
LTR/Gypsy
Unknown
DNA
DNA/PIF-Harbinger
DNA/CMC-EnSpm
RC/Helitron
DNA/hAT
LINE/R2-Hero
Satellite
DNA/hAT-Charlie
DNA/Zisupton
LTR/Copia
DNA/MULE-MuDR
LINE/I
DNA/Crypton-A
DNA/TcMar-Fot1
DNA/Kolobok
DNA/PIF-ISL2EU
DNA/IS3EU
LTR
DNA/hAT-Blackjack
DNA/Crypton-V
DNA/Sola-2
DNA/hAT-hAT5
SINE/MIR
LINE/I-Jockey
DNA/TcMar-Tc2
DNA/Crypton
LINE/RTE-BovB
DNA/hAT-Tag1
DNA/hAT-hAT6
DNA/Kolobok?
DNA/PIF
DNA/Crypton-H
DNA/TcMar-Tigger
DNA/Sola-1
Retroposon
SINE/ID
LTR/Gypsy?
SINE/tRNA-L2
PLE/Chlamys
DNA/Kolobok-T2?
snRNA
SINE/tRNA-RTE
LINE
LTR/ERV
DNA/hAT-Charlie?
DNA/MULE-MuDR?
DNA/hAT-Tip100?
SINE/tRNA-Deu-L2
SINE/tRNA-V
LTR/ERVL
SINE/tRNA-Mermaid
SINE/tRNA-L1
DNA/Zisupton?
ARTEFACT
SINE/tRNA-Mela
0%
25%
50%
75%
100%
0.00E+0
2.50E+7
5.00E+7
7.50E+7
1.00E+8
1.25E+8

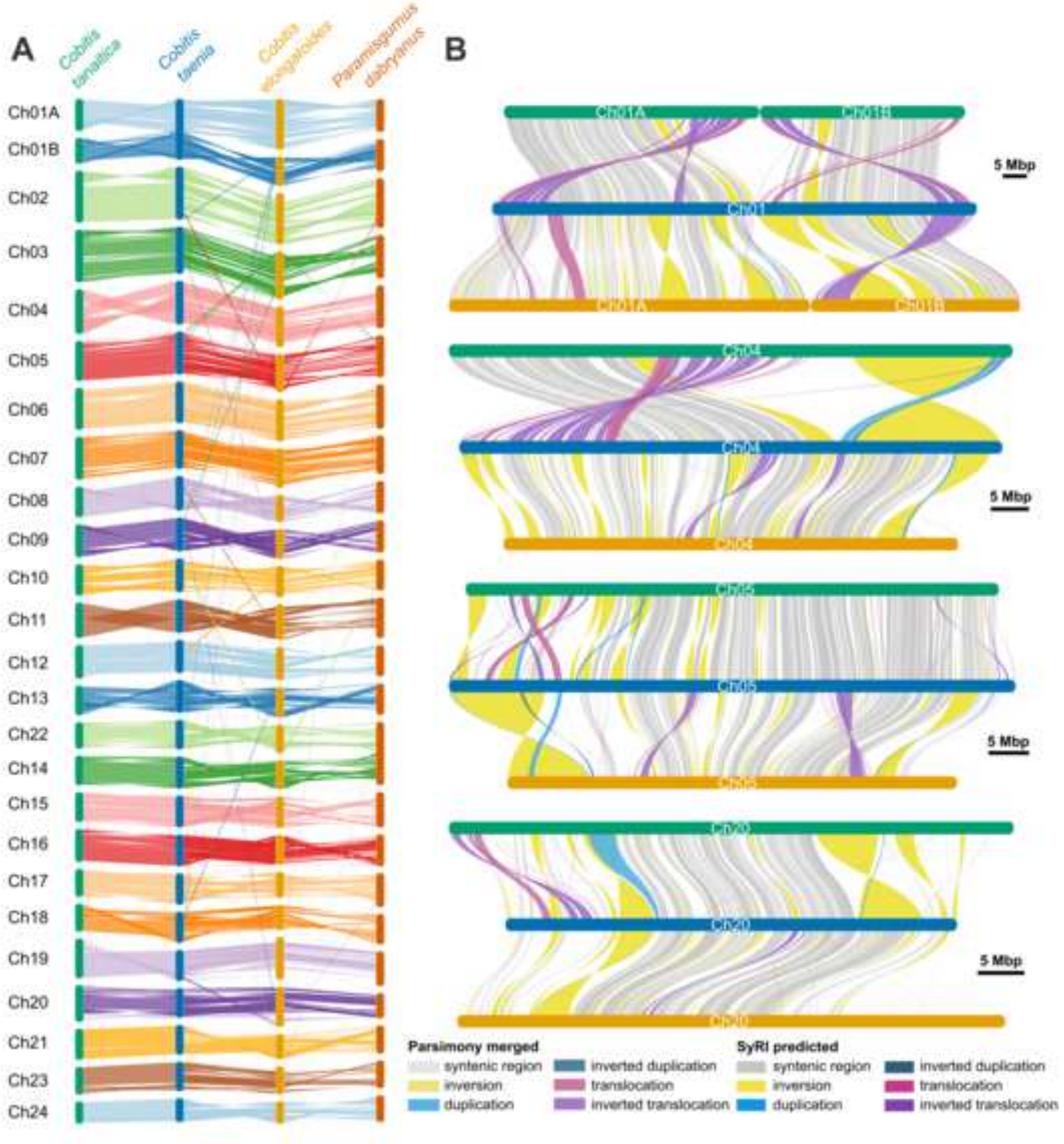

A
Cobitis tanaitica
Cobitis taenia
Cobitis elongatoides
Paramisgurnus dabryanus
Ch01A
Ch01B
Ch02
Ch03
Ch04
Ch05
Ch06
Ch07
Ch08
Ch09
Ch10
Ch11
Ch12
Ch13
Ch22
Ch14
Ch15
Ch16
Ch17
Ch18
Ch19
Ch20
Ch21
Ch23
Ch24
B
Ch01A
Ch01B
Ch01
5 Mbp
Ch04
Ch05
Ch20
Parsimony merged
syntenic region
inversion
duplication
inverted duplication
translocation
inverted translocation
SyRI predicted
syntenic region
inversion
duplication
inverted duplication
translocation
inverted translocation

Figure 5

Click here to access/download;Figure;Figure_5.tiff

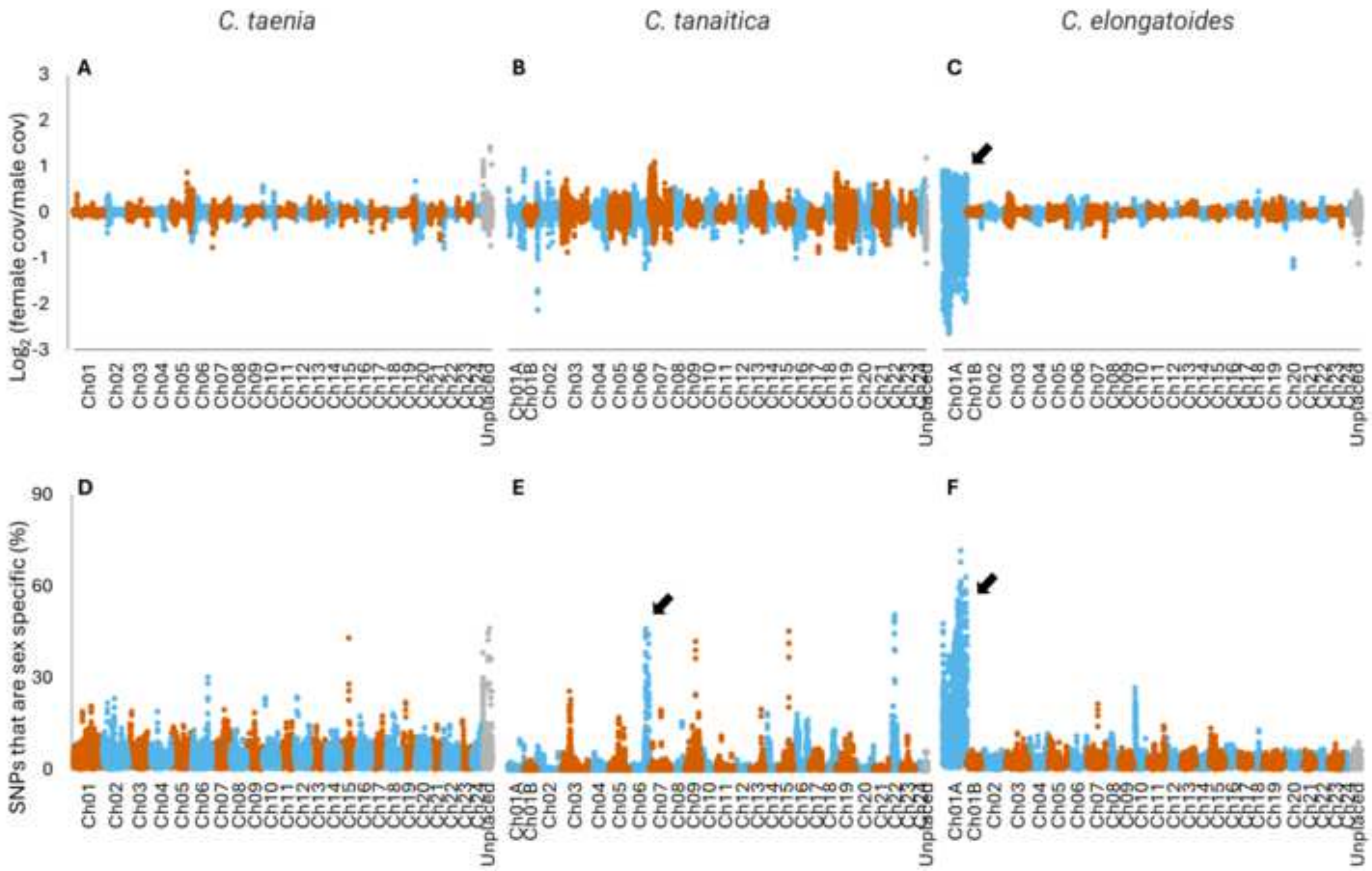

Figure 6

Click here to access/download;Figure;Figure_6.tif

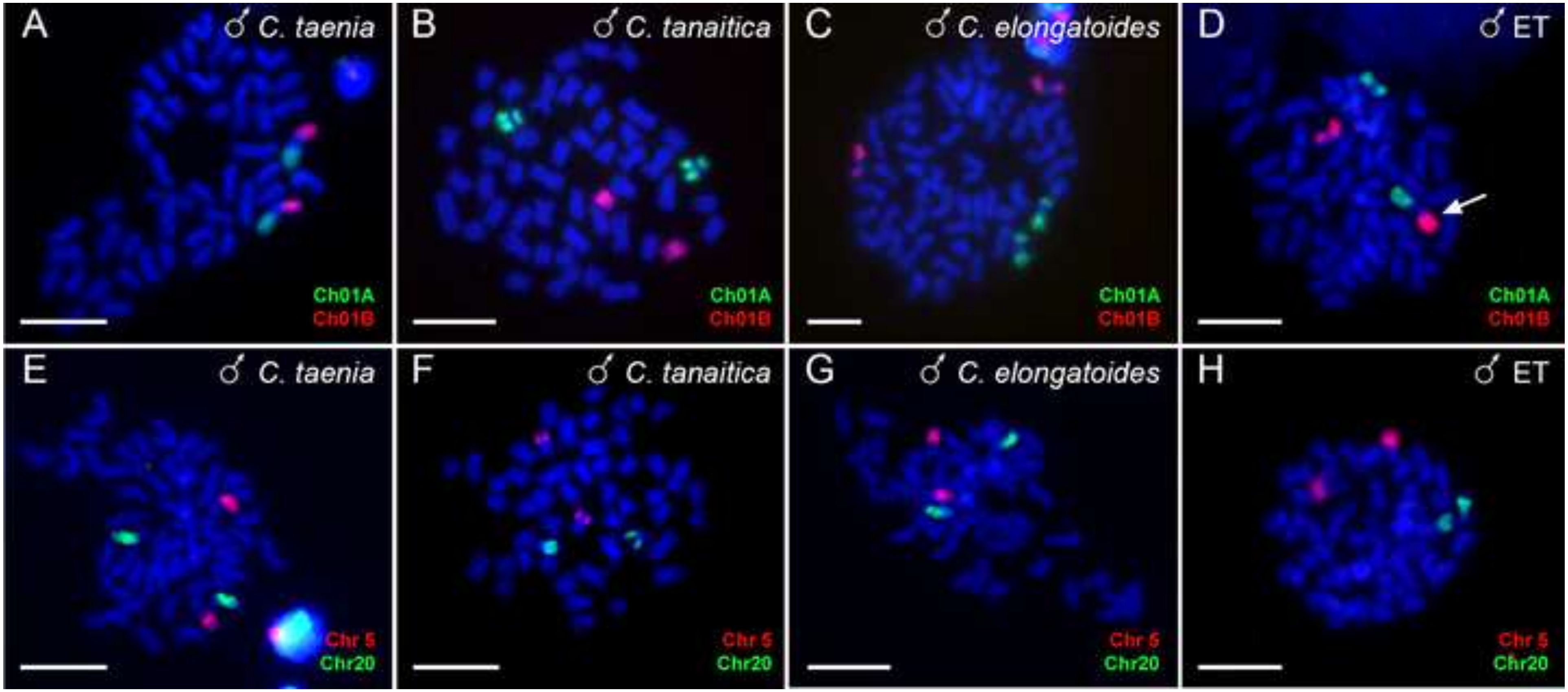

Figure 7 Click here to access/download;Figure;Figure_7.tif

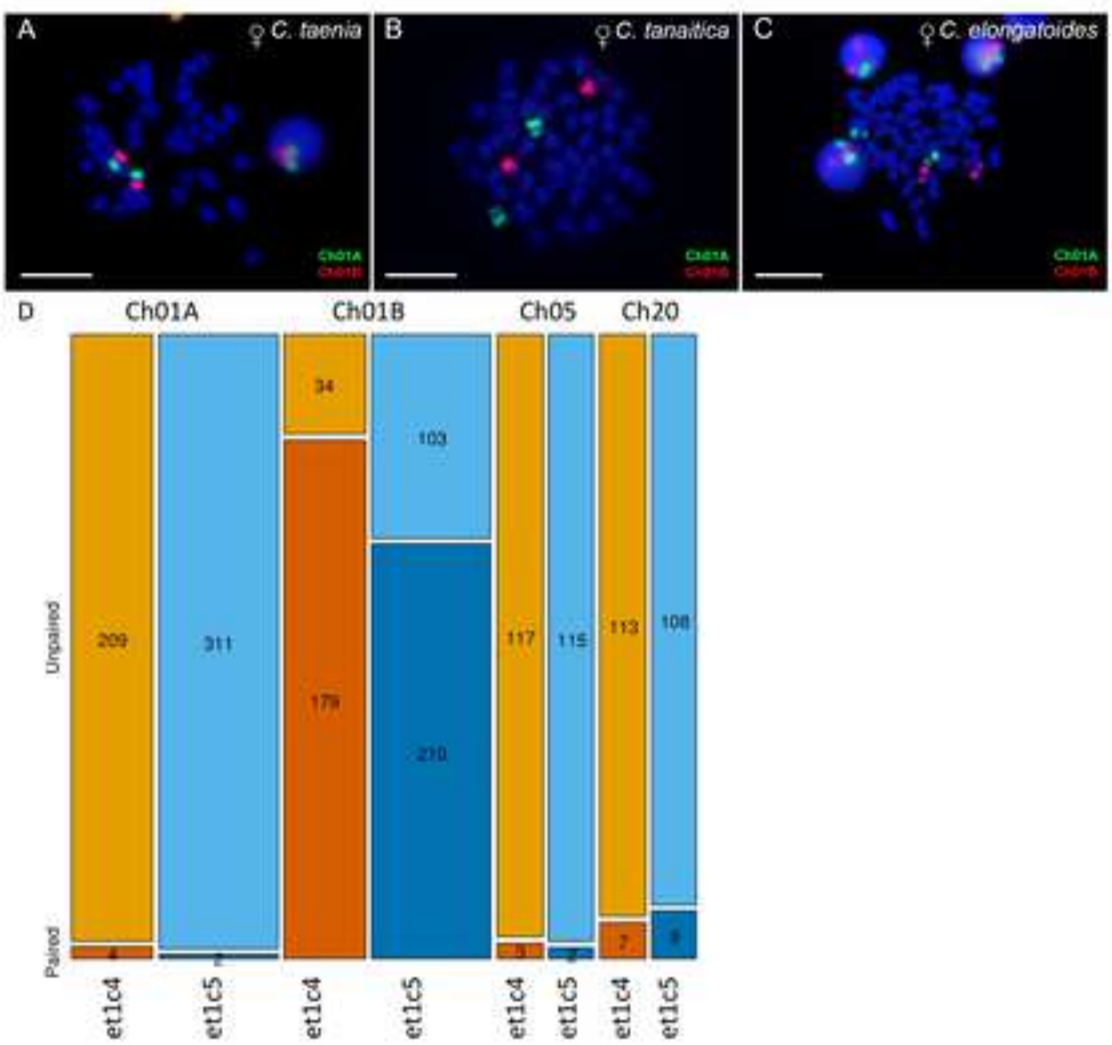

Supplementary Figure S1

Click here to access/download
Supplementary Material
Supplementary_Figure_S1.tiff

Supplementary Figure S2

Click here to access/download
Supplementary Material
Supplementary_Figure_S2.tiff

Supplementary Figure S3

Click here to access/download
Supplementary Material
Supplementary_Figure_S3_small.tiff

Supplementary Figure S4

Click here to access/download
Supplementary Material
Supplementary_Figure_S4.tif

Click here to access/download
Supplementary Material
Supplementary_Tables.xlsx
