# Supplementary material for: Sex chromosome turnover and structural genome divergence shape meiotic outcomes in hybridizing Cobitis
Source: Gigascience. 2026 Mar 24;15:giag031. doi: 10.1093/gigascience/giag031 (PMC13175044; doi:10.1093/gigascience/giag031)
Supplement: giag031_Authors_Response_To_Reviewer_Comments_original_submission [file giag031_authors_response_to_reviewer_comments_original_submission.pdf]

Author's Response To Reviewer Comments

Dear Editors and Reviewers,

We sincerely thank you for your thorough evaluation of our manuscript entitled “Sex Chromosome Turnover and Structural Genome Divergence Shapes Meiotic Outcomes in Hybridising Cobitis.” (GIGA-D-25-00241). We appreciate the constructive feedback and the recommendation for major revision. We have carefully considered all comments and revised the manuscript accordingly to address the concerns raised. Below, we provide a point-by-point response to each comment, detailing the changes implemented.

Reviewer #1 – General Comment
The authors assembled the genomes of three Cobitis species native to Eurasia in an attempt to investigate the effects of structural variants on hybrid meiotic failure. This is certainly an interesting topic given the advances in our abilities to study hybridization that have been enabled by modern genomic sequencing methods, and the evolutionary consequences of asexually-reproducing species that result from rare instances of these hybrid events.
Response:
We thank the reviewer for these appreciative words regarding the significance and relevance of our study.

Reviewer #1 – Comment 1:
The introduction of the manuscript is well-written and focused on the topic at hand. Language was mostly clear throughout the manuscript. However, the paper overall is very lengthy and would benefit from extensive revision. Personally, I think the assembly and annotation of the three genomes is worthy of being a paper (genome report) on its own. Extraction of this material into a separate manuscript would allow the authors to hone the remainder of the paper into a much more concise and focused manuscript.
Response:
We thank the reviewer for this suggestion and fully acknowledge the concern regarding manuscript length. Following the editor’s guidance that separation is not required, we have retained the genome assemblies and downstream analyses within a single integrated manuscript. This decision reflects the strong interdependence between the assemblies and cytogenetic validation: confirming scaffold contiguity was essential for interpreting meiotic pairing in hybrids, and the pairing analyses gain biological meaning only in the context of the assembled chromosomal architecture. For these reasons, we believe the study is best presented as a unified whole.
Nevertheless, we took the comment seriously and substantially streamlined the Introduction, Results, and Discussion to improve the focus and reduce redundancy. Given the extent of these revisions, we do not indicate specific line changes, but the overall manuscript is now considerably more concise.

Reviewer #1 – Comment 2:
Some aspects of the methods section related to genome assembly and annotation could be clarified and/or bolstered. Presentation of methods is mostly clear, but the description of genome annotation methods is a bit tough to follow. This procedure included many complicated steps and may benefit from a flow chart, even if included only as a supplemental figure.
Response:
We thank the reviewer for highlighting the need for clearer presentation of the genome assembly and annotation workflow. To address this, we have revised these sections for improved clarity, reordered several steps for better readability, and expanded descriptions where necessary. These changes are reflected on lines 218–308 of the revised manuscript. We did not include an additional figure with a schematic flowchart because our pipeline is quite standard and should be easily followable from the Methods section.

Reviewer #1 – Comment 3:
Several important quality control steps pertaining to genome assembly and DNA/RNA sequence processing were not mentioned. Authors do not report methods used for quality filtering or trimming. They do not report any process for removal of sequencing adapters. Additionally, they do not report screening of the genome assemblies for contamination from other species. These are critical steps in producing high-quality

genome assemblies that need to be addressed.
Response:
We thank the reviewer for emphasizing the importance of quality control in genome assembly. In response, we have added details on quality testing, filtering, and adapter trimming to the Methods section (lines 179 and 198;). No contamination filtering was performed, as preliminary assemblies showed a high proportion of reads mapping to the expected Cobitis sequences, indicating negligible contamination. Furthermore, all three genomes were consistent in length and content, and chromosome sizes correlated well with cytogenetic observations, supporting the absence of significant contamination.

Reviewer #1 – Comment 4:
Presentation of statistics describing genome assembly quality, contiguity, and completeness could be improved. Authors might want to take some inspiration from statistics required for reporting in genome reports published by other journals, such as G3 or Genome Biology and Evolution. Sequencing depth is not reported in any context for the initial assemblies. Only log-transformed values are available in a single figure. Throughout the manuscript, authors conflate sequencing coverage (the proportion of a genome or genomic region that has been sequenced) with sequencing depth (the number of times a base or genomic region has been sequenced).
Response:
We thank the reviewer for pointing out these important issues. We have updated the assembly statistics to include additional metrics such as Largest Contig, N90, L90, GC content, and BUSCO gene scores. We have also corrected terminology by replacing “coverage” with “depth” where appropriate and reported sequencing depth explicitly in the relevant sections (lines 218–238; lines 241 and 256; lines 403-404). These revisions align the manuscript more closely with reporting standards used in genome-focused journals.

Reviewer #1 – Comment 5:
For the sex-linked primers designed by the authors - I would recommend development of an internal positive control that would be expected to amplify in both sexes and be easily distinguishable from the sex-linked locus by size or fluorescent label. This allows the users to distinguish between failed PCRs and identification of the homogametic sex. This is especially important because the fish selected for marker development were collected from a relatively small portion of the species' distributions (Figure 1) so there could be population-specific differences that affect reliability of these markers for identifying sex. This is a problem I regularly encounter in my own work for wide-ranging species.
Response:
We appreciate the reviewer’s recommendation regarding inclusion of an internal positive control for the sex-linked PCR assay. In our study, all individuals were independently validated through standard genotyping prior to sex-marker testing: each specimen was successfully genotyped using a panel of microsatellite markers and a Sanger-sequenced nuclear locus (rps7 intron), all of which are PCR-based assays. Only individuals whose DNA consistently amplified in these established markers were included; any sample failing these tests was excluded because its genotype could not be verified. Thus, successful microsatellite and rps7 genotyping effectively served as internal confirmation of DNA quality and PCR suitability for all individuals analyzed. We have added this clarification to the Methods section (“RNA and DNA isolation and sequencing,” lines 175–178).

Reviewer #1 – Comment 6:
I was also surprised that the authors did not conduct a GWAS analysis. That seems to be a fairly typical analysis included in studies of this type to elucidate sex-linked SNPs. It would add to an already extensive manuscript; however, this could add an additional argument for splitting this manuscript in two. It would provide more space to include it in a more focused manuscript.
Response:
We thank the reviewer for this suggestion and agree that GWAS can be informative in studies of sex-linked loci. However, GWAS was not feasible in our case for methodological reasons. We employed a Pool-Seq design to reduce sequencing costs and maximize genome-wide coverage across species. Because Pool-Seq aggregates DNA from multiple individuals, it does not provide individual-level genotype data required for SNP–phenotype association tests such as GWAS. Instead, our approach leverages allele-frequency differences between male and female pools, which efficiently identifies sex-linked SNPs and chromosomal regions while minimizing sampling noise typical of small individual-based datasets. This strategy revealed

clear, species-specific sex-linked regions and turnover patterns that directly support our study's aims.
We acknowledge that an individual-based approach could, in principle, be applied to C. tanaitica, which was not sequenced using Pool-Seq. However, the number of available specimens was too limited to support a statistically meaningful GWAS. For this reason, we refrained from overinterpreting results for C. tanaitica, aside from noting its non-homologous sex-determination system relative to C. elongatoides.

Reviewer #1 – Comment 7:
The results section contains many statements that would be more appropriate in the Methods section, or could be deleted entirely because they are redundant with statements already present in the Methods section. Additionally, there are some sentences that are more appropriate for inclusion in the Discussion section because they are interpretive. I have included examples under the 'Minor comments' section of this review. Some of the material presented as results in the Supplementary tables is presented in a confusing manner, and appears to contain errors (see examples in 'Minor comments' section below).
Response:
We thank the reviewer for these helpful observations. We have revised the Results and Methods sections to remove redundancies, relocated interpretive statements to the Discussion, and corrected the issues identified in the Supplementary tables as per the minor comments. These changes improve clarity and ensure that each section adheres to its intended purpose.

Reviewer #1 – Comment 8:
The first several paragraphs of the Discussion section either repeat material already covered in the Results section, or go on tangents that are not directly related to the main purpose of the paper. However, some of it could be more appropriate to include in a genome report if the authors split the manuscript in two.
Response:
We thank the reviewer for this observation. The Discussion section has been thoroughly rewritten to remove redundancies, eliminate tangential content, and improve focus. These revisions affect the entire section; therefore, we do not provide specific line references.

Reviewer #1 – Comment 9:
Given the above issues, I find that the paper needs extensive editing and possibly more analytical work (if some of the methodological deficiencies were overlooked in the analysis phase as well as the writing phase of this project). It is unlikely this work could be accomplished in the normal window for a revision. Therefore, I regrettably suggest rejection of the manuscript.
Response:
We appreciate the reviewer's candid assessment and acknowledge the concerns raised. We have undertaken extensive revisions to address all major and minor comments, including clarifying methods, improving presentation of results, streamlining the manuscript, and correcting issues in supplementary materials. We hope that these substantial changes satisfactorily resolve the identified deficiencies and demonstrate the robustness of our analyses. We respectfully ask the reviewer to reconsider the manuscript in light of these improvements.

Reviewer #1 - Minor Comments
We thank the reviewer for the detailed minor comments, which helped improve clarity and accuracy throughout the manuscript. All suggested changes have been implemented as follows:
- Added missing version numbers for Hisat2, D-Genies, SyRI, and NGenomeSyn.
- Included additional assembly metrics (N90, L90) in Table 1.
- Corrected genome statistics and clarified the definition of "chromosome-level" assemblies; updated Supplementary Table S11 accordingly.
- Fixed terminology (e.g., replaced "coverage" with "depth"), removed redundancies, and relocated interpretive statements to the Discussion.
- Deleted repeated sentences and tangential content; streamlined Results and Discussion sections.
- Corrected typographical issues (e.g., "less" → "fewer," removed "loci's," removed redundant PMER definition).
- Removed novelty claim ("the first of their kind") as suggested.
- Standardized reference formatting and species name italicization across text, figures, and tables.
- Corrected numerical formatting in Table 2 and errors in Supplementary Tables S7–S11; added missing row descriptions.

• Updated figure captions and supplementary materials for clarity.
These revisions address all minor points raised by the reviewer.

Reviewer #2 – General Comment:
This study presents the first chromosome-level genome assemblies for three hybridising Cobitis species (C. elongatoides, C. taenia, and C. tanaitica) to investigate the genomic and cytogenetic basis of hybrid sterility and the transition to asexuality. They provide large amount of integrated data including genome scaffolding, male/female pooled sequencing (Pool-Seq), and molecular cytogenetics, and found extensive structural variation among homologous chromosomes of the three species, despite overall karyotype conservation. They further used population-level Pool-Seq analyses further revealed that each species possesses distinct, non-homologous sex chromosomes. Overall, the analyses are comprehensive and results are solid, which is suitable to this journal.
Response:
We sincerely thank the reviewer for these positive and encouraging comments regarding the scope, quality, and relevance of our study.

Reviewer #2 – Minor Comments
Comment 1: The Background is too long, with many short paragraphs; you can shorten it to 4–5 paragraphs.
Response:
Thank you for this comment, which also mirrors the suggestion of Reviewer #1. We have shortened and streamlined the Introduction to improve focus and readability.

Comment 2: Methods: there is no Ethics statement; please add it.
Response:
We added an Ethics statement in the dedicated section of the manuscript (lines 930-933).

Comment 3: Table 1 should be moved to supplementary files.
Response:
We moved Table 1 to the supplementary materials; it is now Supplementary Table S4.

Comment 4: Figure 4 is not easy to see.
Response:
Figure 4 has been improved for clarity and divided into two figures: the main figure is now Figure 3, and the additional detail is provided as Supplementary Figure S1.

We thank the reviewers and editors for their constructive feedback, which has significantly improved the manuscript. We believe the revised version addresses all concerns and strengthens the clarity, rigor, and impact of the study. We hope the manuscript is now suitable for publication and remain available for any further clarifications.

Sincerely,
Stephen Schlebusch, Vladimir Trifonov, Zuzana Halenková, Marharyta Klianitskaya, Dmitrij Dedukh, Aurora Ruiz-Herrera, Lucia Álvarez-González, Gala Pujol, Eva Hřibová, Lucija Andjel, Oldřich Bartoš, Petr Pajer, Tomáš Tichopád, Daniel Kulik, Jan Kotusz, Marie Kaštánková Doležálková, Astrid Böhne, Anatolie Marta, Patrik Horna, Radka Reifová, Yann Guiguen, Heiner Kuhl, Jan Pačes, and Karel Janko

Cookie Preference Center

We use cookies which are necessary to make our site work. We may also use additional cookies to analyze, improve and personalize our content and your digital experience. For more information, see our Cookie Policy.

You may choose not to allow some types of cookies. However, blocking some types may impact your experience of our site and the services we are able to offer. See the different category headings below to find out more or change your settings.

Allow all

Manage Consent Preferences

Strictly Necessary Cookies

Always active

These cookies are necessary for the website to function and cannot be switched off in our systems. They are usually only set in response to actions made by you which amount to a request for services, such as setting your privacy preferences, logging in or filling in forms. You can set your browser to block or alert you about these cookies, but some parts of the site will not then work.

Functional Cookies

These cookies enable the website to provide enhanced functionality and personalisation. They may be set by us or by third party providers whose services we have added to our pages. If you do not allow these cookies then some or all of these services may not function properly.

Performance Cookies

These cookies allow us to count visits and traffic sources so we can measure and improve the performance of our site. They help us to know which pages are the most and least popular and see how visitors move around the site.

Targeting Cookies

These cookies may be set through our site by our advertising partners. They may be used by those companies to build a profile of your interests and show you relevant adverts on other sites. If you do not allow these cookies, you will experience less targeted advertising.

Cookie List

Clear

Apply Cancel
Consent Leg.Interest

Confirm my choices

Power
