# Supplementary material for: Sex chromosome turnover and structural genome divergence shape meiotic outcomes in hybridizing Cobitis
Source: Gigascience. 2026 Mar 24;15:giag031. doi: 10.1093/gigascience/giag031 (PMC13175044; doi:10.1093/gigascience/giag031)
Supplement: giag031_Reviewer_1_Report_original_submission — Reviewer 1 -- 8/26/2025 [file giag031_reviewer_1_report_original_submission.pdf]

Reviewer Report

Title: Sex Chromosome Turnover and Structural Genome Divergence Shapes Meiotic Outcomes in Hybridising Cobitis

Version: Original Submission **Date:** 8/26/2025

Reviewer name: Reviewer 1

Reviewer Comments to Author:

The authors assembled the genomes of three Cobitis species native to Eurasia in an attempt to investigate the effects of structural variants on hybrid meiotic failure. This is certainly an interesting topic given the advances in our abilities to study hybridization that have been enabled by modern genomic sequencing methods, and the evolutionary consequences of asexually-reproducing species that result from rare instances of these hybrid events.

Major comments:

The introduction of the manuscript is well-written and focused on the topic at hand. Language was mostly clear throughout the manuscript. However, the paper overall is very lengthy and would benefit from extensive revision. Personally, I think the assembly and annotation of the three genomes is worthy of being a paper (genome report) on its own. Extraction of this material into a separate manuscript would allow the authors to hone the remainder of the paper into a much more concise and focused manuscript.

Some aspects of the methods section related to genome assembly and annotation could be clarified and/or bolstered. Presentation of methods is mostly clear, but the description of genome annotation methods is a bit tough to follow. This procedure included many complicated steps and may benefit from a flow chart, even if included only as a supplemental figure.

Several important quality control steps pertaining to genome assembly and DNA/RNA sequence processing were not mentioned. Authors do not report methods used for quality filtering or trimming. They do not report any process for removal of sequencing adapters. Additionally, they do not report screening of the genome assemblies for contamination from other species. These are critical steps in producing high-quality genome assemblies that need to be addressed.

Presentation of statistics describing genome assembly quality, contiguity, and completeness could be improved. Authors might want to take some inspiration from statistics required for reporting in genome reports published by other journals, such as G3 or Genome Biology and Evolution. Sequencing depth is not reported in any context for the initial assemblies. Only log-transformed values are available in a single figure. Throughout the manuscript, authors conflate sequencing coverage (the proportion of a genome or genomic region that has been sequenced) with sequencing depth (the number of times a base or genomic region has been sequenced).

For the sex-linked primers designed by the authors - I would recommend development of an internal positive control that would be expected to amplify in both sexes and be easily distinguishable from the sex-linked locus by size or fluorescent label. This allows the users to distinguish between failed PCRs and identification of the homogametic sex. This is especially important because the fish selected for marker

development were collected from a relatively small portion of the species' distributions (Figure 1) so there could be population-specific differences that affect reliability of these markers for identifying sex. This is a problem I regularly encounter in my own work for wide-ranging species.

I was also surprised that the authors did not conduct a GWAS analysis. That seems to be a fairly typical analysis included in studies of this type to elucidate sex-linked SNPs. It would add to an already extensive manuscript; however, this could add an additional argument for splitting this manuscript in two. It would provide more space to include it in a more focused manuscript.

The results section contains many statements that would be more appropriate in the Methods section, or could be deleted entirely because they are redundant with statements already present in the Methods section. Additionally, there are some sentences that are more appropriate for inclusion in the Discussion section because they are interpretive. I have included examples under the 'Minor comments' section of this review. Some of the material presented as results in the Supplementary tables is presented in a confusing manner, and appears to contain errors (see examples in 'Minor comments' section below).

The first several paragraphs of the Discussion section either repeat material already covered in the Results section, or go on tangents that are not directly related to the main purpose of the paper. However, some of it could be more appropriate to include in a genome report if the authors split the manuscript in two.

Given the above issues, I find that the paper needs extensive editing and possibly more analytical work (if some of the methodological deficiencies were overlooked in the analysis phase as well as the writing phase of this project). It is unlikely this work could be accomplished in the normal window for a revision. Therefore, I regrettably suggest rejection of the manuscript.

Finally, I have no meaningful experience with FISH probes or chromosomal painting so unfortunately, I can't provide much comment on those portions of the paper.

Minor comments:

Line 291: please provide specific version number for Hisat2

Line 319: version numbers for D-Genies and SyRI missing

Line 331: version number for NGenomeSyn missing

Line 439-440: Authors provide N50 values, but the paper would benefit from providing some additional metrics, such as N90 and L90, to help readers gauge the contiguity of these genomes.

Line 442 - 443: I'm having a hard time understanding how the authors are calling these 'chromosome-level' assemblies when nearly a third (>30%) of the genome of two species (C. tanaitica and C. elongatoides) could not be assembled into chromosomal scaffolds.

Line 457 - 458: Either the term 'topologically associated domains' is missing, or the authors need to remove the parentheses from around TADs if it was defined earlier in the manuscript.

Line 470: change 'less' to 'fewer'

Line 483 - 486: The statements that observed patterns of repeat families 'suggest' something are interpretive and should be moved to the discussion.

Line 499 - 500: This sentence repeats content of the methods section. I suggest deleting it.

Line 540 - 564: If I am understanding correctly, the discussion of 'coverage' here would be more accurately described as 'depth' since the authors seem to be talking about average sequencing depth in different areas of the genome. Furthermore, authors never provide untransformed measures of

sequencing depth in any context (the initial genome assemblies, pool-seq data, re-sequenced individuals, etc.). Therefore, it is difficult to determine if the differences being discussed here are derived from data with enough statistical power to measure differences in sequencing depth between male and female fish.
Lines 614 - 619: This could be explored with GWAS
Lines 635 - 641: Much of this paragraph is a description of methods and belongs in the Methods section.
Lines 664 - 667: Much of this is interpretive - more appropriate for the discussion.
Lines 700 - 711: This paragraph has little or no relevance to the main topic of this paper (hybrid meiotic failure).
Line 745: remove "loci's"
Line 813 - 815: PMER was already defined earlier in the paper.
Line 854: I suggest removal of "the first of their kind in an asexually reproducing vertebrate," because such statements rarely age well, and the concept behind the paper is interesting enough to stand on its own without pointing out the novelty of it being the 'first' time it was detected.
References section: Capitalization of article titles varies from one reference to the next. Scientific names are sometimes italicized; other times they are not.
Table 2: 'L50' and 'Number of Chromosomes' are always going to be integers. Why are there two significant digits to the right of the decimal point?
Supplementary Figure S2: 'Cobitis' should be italicized.
Supplementary Table S7: This table presents pre- and post-HiC values in a confusing manner that is nonsensical and probably erroneous. For example, the N50 values seem problematic. How do you have a 154 Kbp pre-HiC N50 contig value for C. elongatoides, but a 154 Mbp post-HiC N50 contig value for the same species? This is longer than the longest reported chromosome for any species (C. taenia) in Supplementary Table S8 (99 Mbp).
Supplementary Table S10: I don't know what the percentages in line 33 refer to?

Methods

Are the methods appropriate to the aims of the study, are they well described, and are necessary controls included? Choose an item.

Conclusions

Are the conclusions adequately supported by the data shown? Choose an item.

Reporting Standards

Does the manuscript adhere to the journal's guidelines on [minimum standards of reporting?]() Choose an item.

Choose an item.

Statistics

Are you able to assess all statistics in the manuscript, including the appropriateness of statistical tests used? Choose an item.

Quality of Written English

Please indicate the quality of language in the manuscript: Choose an item.

Declaration of Competing Interests

Please complete a declaration of competing interests, considering the following questions:

- Have you in the past five years received reimbursements, fees, funding, or salary from an organisation that may in any way gain or lose financially from the publication of this manuscript, either now or in the future?
- Do you hold any stocks or shares in an organisation that may in any way gain or lose financially from the publication of this manuscript, either now or in the future?
- Do you hold or are you currently applying for any patents relating to the content of the manuscript?
- Have you received reimbursements, fees, funding, or salary from an organization that holds or has applied for patents relating to the content of the manuscript?
- Do you have any other financial competing interests?
- Do you have any non-financial competing interests in relation to this paper?

If you can answer no to all of the above, write 'I declare that I have no competing interests' below. If your reply is yes to any, please give details below.

I declare that I have no competing interests

I agree to the open peer review policy of the journal. I understand that my name will be included on my report to the authors and, if the manuscript is accepted for publication, my named report including any attachments I upload will be posted on the website along with the authors' responses. I agree for my report to be made available under an Open Access Creative Commons CC-BY license (http://creativecommons.org/licenses/by/4.0/). I understand that any comments which I do not wish to be included in my named report can be included as confidential comments to the editors, which will not be published.

Choose an item.

To further support our reviewers, we have joined with Publons, where you can gain additional credit to further highlight your hard work (see: https://publons.com/journal/530/gigascience). On publication of this paper, your review will be automatically added to Publons, you can then choose whether or not to claim your Publons credit. I understand this statement.

Yes Choose an item.
